# Elucidating assembly and function of VirB8 cell wall subunits refines the DNA translocation model in Gram-positive T4SSs

**DOI:** 10.1126/sciadv.adq5975

**Published:** 2025-01-22

**Authors:** Robine Maffo-Woulefack, Abbas Mohamad Ali, Haifa Laroussi, Julien Cappèle, Felipe Romero-Saavedra, Nancy Ramia, Emilie Robert, Sandrine Mathiot, Nicolas Soler, Yvonne Roussel, Rémi Fronzes, Johannes Huebner, Claude Didierjean, Frédérique Favier, Nathalie Leblond-Bourget, Badreddine Douzi

**Affiliations:** ^1^ Université de Lorraine, INRAE, DynAMic, F-54000 Nancy, France.; ^2^ Université de Lorraine, CNRS, CRM2, F-54000 Nancy, France.; ^3^ Division of Pediatric Infectious Diseases, Dr. von Hauner Children’s Hospital, Ludwig-Maximillians University, Munich, Germany.; ^4^ Institut Européen de Chimie et Biologie, University of Bordeaux, Pessac, France.; ^5^ CNRS UMR 5234 Microbiologie Fondamentale et Pathogénicité, Bordeaux, France.

## Abstract

Bacterial type IV secretion systems (T4SSs) are widespread nanomachines specialized in the transport across the cell envelope of various types of molecules including mobile genetic elements during conjugation. Despite their prevalence in Gram-positive bacteria, including relevant pathogens, their assembly and functioning remain unknown. This study addresses these gaps by investigating VirB8 proteins, known to be central components of conjugative T4SSs in Gram-positive bacteria. However, the functional packing and precise role of VirB8 in T4SSs biology remain undefined. Our findings elucidate the nature of VirB8 proteins as cell wall components, where they multimerize and exhibit a conserved assembly pattern, distinct from VirB8 in Gram-negative bacteria. We also demonstrate that VirB8 proteins interact with other T4SS subunits and DNA, indicating their pivotal role in the building of the DNA translocation channel across the cell wall. We lastly propose a distinct architecture for conjugative T4SSs in Gram-positive bacteria compared to their Gram-negative counterparts, possibly attributed to the differences in the cell wall structure.

## Teaser

Structure-function exploration of VirB8-like subunits paves the way to better understand T4SSs in Gram-positive bacteria.

## INTRODUCTION

Conjugation is an important mechanism of lateral gene transfer in prokaryotes where DNA is transferred from a cell to another in a contact-dependent process (*1*). It plays a pivotal role in bacterial genome plasticity, by enabling rapid and extensive spread of conjugative mobile genetic elements, such as plasmids and integrative and conjugative elements (ICEs) (*2*, *3*). These mobile elements are recognized as the main vectors for disseminating antimicrobial resistance leading to the emergence of multidrug-resistant pathogens, a major concern in public health (*4*). A comprehensive and detailed understanding of the conjugative process is essential for designing prevention and therapeutic approaches aimed at curtailing the spread of antimicrobial resistance.

From a molecular perspective, bacterial conjugation involves a specialized multiprotein complex belonging to the family of type IV secretion systems (T4SSs) (*5*). The structure and function of conjugative systems from Gram-negative bacteria have been studied extensively (*5*–*11*). They have been divided into two groups: minimal and expanded systems (*5*). Following the nomenclature used for the canonical VirB-D4 system found in *Agrobacterium tumefaciens*, minimal systems comprise 12 core subunits denoted as VirB1 to VirB11 and VirD4, the coupling protein. In contrast, expanded systems contain many more proteins requiring up to 25 subunits for proper function and assembly (*11*). Recent advances in structural biology have provided a detailed picture of the global architecture of both Gram-negative minimal and expanded T4SSs (*11*–*18*). Both systems exhibit an overall cylindrical structure spanning the entire bacterial envelope, comprising three main complexes: the outer membrane core complex (OMCC) and the inner membrane complex (IMC), interconnected by a stalk (*12*). Moreover, these systems produce additional surface-exposed organelles, such as adhesins and pili, which play an important role in facilitating contact between donor and recipient conjugating cells.

Combining these structural data with functional characterizations, it has been proposed that DNA transfer through T4SSs is a two-step process whereby the DNA of the conjugative element is processed by the relaxosome and then transferred to the T4SS channel, likely mediated by the coupling protein, although the precise mechanisms are still not well understood. Along its translocation route, the DNA is propelled from the IMC to the OMCC which serves as the external gate through which the pilus and/or the DNA are expelled (*5*). The mechanisms underlying the DNA passage into the target cell are still unclear.

In contrast to Gram-negative systems, conjugative T4SSs from Gram-positive bacteria are poorly characterized despite their widespread occurrence (*2*). The vast majority of known T4SSs in Gram-positive bacteria are encoded by conjugative mobile elements (conjugative plasmids and ICEs) (*19*, *20*). These T4SSs have a set of conserved subunits thought to be functionally homologous to VirB1, VirB3, VirB4, VirB6, and VirB8, commonly found in the IMC of minimal T4SSs from Gram-negative bacteria (*9*). No proteins homologous to those recovered in the OMCC or the pilus of Gram-negative stems have been found associated to these systems. Instead, they include accessory proteins specific to Gram-positive bacteria, such as surface exposed adhesins (*2*, *21*). These substantial differences in subunit composition suggest that T4SSs from Gram-positive bacteria assemble into a channel-like structure distinct in architecture from that described in Gram-negative systems (*9*).

In this study, we focused on the study of VirB8 proteins from Gram-positive T4SSs, known as VirB8-like. This family of proteins represents widely conserved subunits in these systems, playing essential role in T4SSs assembly and function (*22*–*25*). The main model of our study is OrfG, the sole VirB8-like protein of the conjugative and integrative element ICE*St3* found in the Gram-positive bacterium *Streptococcus thermophilus*. We also present data on the VirB8-like protein Orf13, a component of Tn*916*, an ICE widespread in *Enterococcus faecalis* and other firmicutes. Through a combination of in vitro and in vivo experiments, we thoroughly characterized VirB8-like proteins in terms of topology, assembly, and interaction networks. Our data confirm that VirB8-like proteins are predominantly extracellular components, assembling into a conserved pattern, which serves as a building block of the DNA translocation channel. On the basis of these findings, we proposed a model of Gram-positive T4SSs outlining the similarities and the peculiarities in architecture compared to Gram-negative systems. In addition, we discussed the potential structural adaptation of Gram-positive T4SSs in response to variations in envelope composition.

## RESULTS

### VirB8-like proteins are highly diverse in Gram-positive T4SSs

**Fig. 1.**
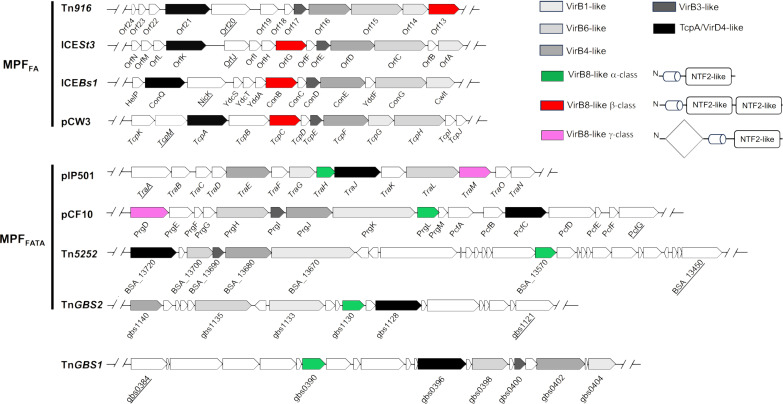
Genetic organization of conjugative T4SSs from Gram-positive bacteria. Elements are classified in MPF_FA_ and MPF_FATA_ types as described in (*20*). Genes encoding putative T4SS proteins are highlighted by different shades of gray and, in the case of VirB8-like proteins, by colored arrows (red for VirB8-like from β-class, green for α-class, and pink for γ-classe). Their domain composition is shown in the figure where cylinders represent transmembrane domains (TMDs), rectangles NTF2-like domains, and the diamond a domain of unknown function. The proteins corresponding to the relaxases are underlined.

The VirB8 protein family is proposed to be a central component of conjugative T4SSs in both Gram-negative and Gram-positive bacteria (*22*–*26*). In contrast to VirB8 proteins from Gram-negative systems, which contain a single NTF2-like domain—the hallmark of VirB8 protein family—VirB8-like proteins from Gram-positive systems exhibit greater diversity in terms of domain composition. They are categorized into α-, β-, and γ-classes (Fig. 1) (*24*, *25*). To assess the distribution of these proteins, we conducted a detailed study of T4SS clusters found in mobile conjugative elements from Gram-positive bacteria. Our dataset comprised conjugative T4SSs from the two main phylogenetic clades MPF_FA_ and MPF_FATA_. Here, MPF denotes mating pore formation with MPF_FA_ representing MPF from Firmicutes (F) and Actinobacteria (A) and MPF_FATA_ including MPF from Firmicutes, Actinobacteria, Tenericutes (T), and Archaea (A) (*20*). As illustrated in Fig. 1, VirB8-like proteins were identified in both MPF_FA_ and MPF_FATA_. In addition, our results revealed that the MPF_FA_ was the sole clade where VirB8-like proteins from the β-class were present. Furthermore, we observed that every element from the MPF_FATA_ encodes a VirB8-like protein from the α-class, either independently or in conjunction with a VirB8-like from the γ-class (Fig. 1). A gene encoding a VirB8-like protein from the α-class is found within Tn*GBS1*, the unique representative element from a third clade of T4SS, apart from MPF_FA_ and MPF_FATA_ indicating that VirB8-like proteins are found in all T4SSs clades (Fig. 1).

### OrfG is a membrane protein that exposes its C-terminal region on the bacterial surface

#### Topology analysis

**Fig. 2.**
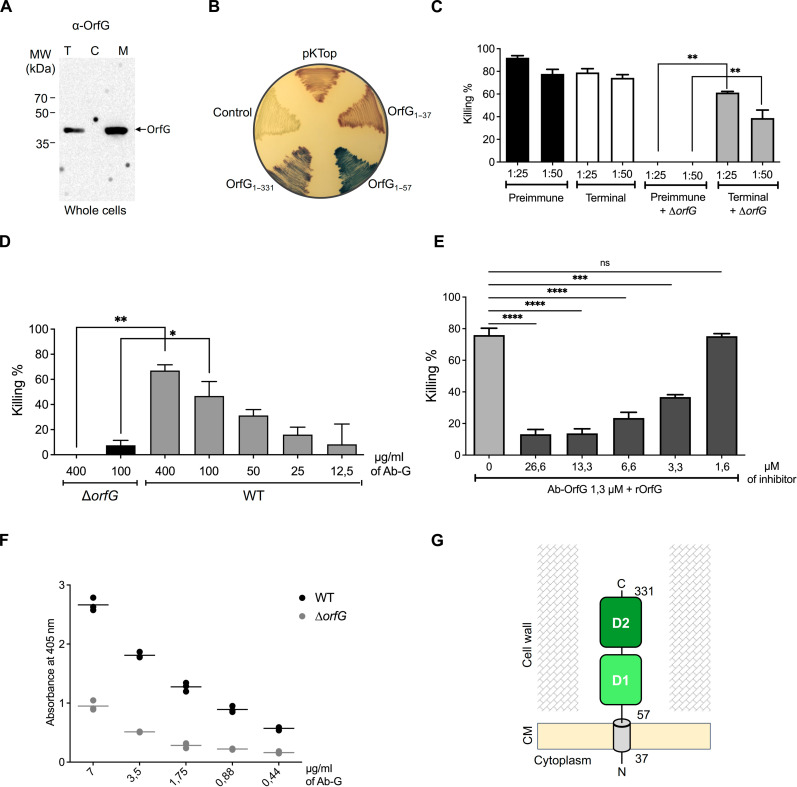
Topology analysis of OrfG. (**A**) Immunoblotting of the total (T), cytoplasmic (C), and membrane (M) fractions of the WT strain using specific polyclonal antibodies directed against OrfG (α-OrfG). The molecular mass markers (kilodaltons) are indicated on the left. MW, molecular weight. (**B**) Full-length OrfG or its truncated versions were fused in frame with the PhoA-LacZ dual reporter in the pKTOP vector. *E. coli* DH5α transformants containing a control vector (pET28) or expressing the different *phoA-lacZ* fusions were plated on lysogeny broth (LB) agar plates supplemented with the chromogenic substrates Salmon-Gal for LacZ and X-Pho for PhoA. Red coloration of colonies indicates the cytoplasmic localization of PhoA-LacZ, whereas blue coloration indicates a periplasmic localization. (**C**) OPA results using immune sera are represented by plotting sera dilutions in the *x* axis and the effectiveness of killing (in percent) in the *y* axis for each dilution. Three types of sera were used: the preimmune sera in black correspond to the sera collected before immunization, the terminal sera in white correspond to the sera collected after immunization, and the preimmune + Δ*G* sera (in gray) is the preimmune sera incubated with Δ*orfG* strain to remove the nonspecific antibodies. (**D**) OPA results using purified anti-OrfG antibodies (Ab-G) performed on Δ*orfG* or WT strains. Ab-G was purified from the terminal sera using a purification kit (Hook agarose coupling kit from G-Biosciences). OPA results are represented by plotting the concentration of the antibody (Ab-G) on the *x* axis, and the effectiveness of opsonophagocytic killing represented by gray color for the WT strain and black color for the Δ*orfG* strain on the *y* axis. (**E**) Inhibition of opsonophagocytic killing using Ab-G mixed with purified recombinant OrfG_Ext_. Ab-G at a concentration yielding opsonic killing activities ranging from 40 to 60% against WT strain were incubated with different amounts of the inhibitor (OrfG_Ext_). Opsonophagocytic killing observed in the absence of OrfG_Ext_, shown in light gray, was used as an internal control for OPA experiments. OPA performed in the presence of Ab-G preincubated with increasing concentrations of OrfG_Ext_ is represented in dark gray. The final inhibitor concentration is shown below the *x* axis, as well as the antibody concentration used for each set of samples. For OPA in (C), statistical analysis was made between OPA using preimmune and terminal immune sera at the same concentration. For OPA using purified Ab-G (E), statistical analysis was made by comparing the OPA results obtained with preincubation with the inhibitor to that OPA without preincubation with the inhibitor. (**F**) Whole bacterial ELISA. The binding of different concentrations of Ab-G on WT or Δ*orfG* strains was quantified using ELISA. The increasing concentrations of antibodies shown in the *x* axis were tested on each strain. The absorbance values shown on the *y* axis represent the difference in absorbance between the conditions with antibody to that without (blank) for each strain tested. The antibody was plated in a threefold serial dilution for each concentration, and each dot indicates a repeat. The horizontal lines represent the average of the points at each concentration for the WT (black dots) and Δ*orfG* (gray dots) strains. (**G**) Schematic representation of the topology of OrfG in WT strain where N and C indicate the N- and C-terminal ends. Bars and whiskers denote mean values and SEM; not significant (ns) (*P* ≥ 0.05), *P <0.05, **P < 0.01, ***P < 0.001, and ****P < 0.0001.

ICE*St3* belonging to the MPF_FA_ clade encodes a single VirB8-like protein named OrfG. Bioinformatic analysis using constrained consensus TOPology prediction (CCTOP) (*27*) predicted that OrfG consists of a single transmembrane α helix (residues 37 to 57) followed by a large soluble domain (OrfG_64–331_). This soluble region comprises two NTF2-like domains OrfG_64–204_ and OrfG_223–331_ (referred to hereafter as D1 and D2, respectively). This domain composition assigns OrfG as a member of the β-class (Fig. 1) (*24*, *28*). To experimentally investigate the predicted topology of OrfG, we initially examined its cellular localization in *S. thermophilus*. Cell fractionation was carried out on whole-cell lysates of the wild-type (WT) strain (for more details, see Materials and Methods). Using polyclonal antibodies raised against OrfG (Ab-G), Western blot analysis of the total, cytoplasmic, and membrane fractions demonstrated the presence of OrfG in both the total and the membrane fractions, consistent with its transmembrane prediction (Fig. 2A). To provide additional evidence for the membrane localization of OrfG, we examined the cellular localization of the OrfGΔTM variant [OrfG lacking its predicted transmembrane domain (TMD)]. As depicted in fig. S1A, OrfGΔTM was detected exclusively in the total and the cytoplasmic fractions.

Next, the dual reporter Pho-Lac system (*29*) was used to assess the orientation of OrfG relative to the cytoplasmic membrane of *Escherichia coli*. To this end, we cloned into pKTop the full-length *orfG* (*orfG*_1–331_) and two truncated versions, *orfG*_1–63_ and *orfG*_1–37_, encoding the N-terminal extension including or excluding the predicted TMD, respectively. As reported in Fig. 2B, only colonies producing the OrfG variants with the TMD exhibited a blue color indicating their periplasmic localization (Fig. 2B). This observation demonstrates that the TMD enables the export of the OrfG C-terminal domain to the periplasmic space of *E. coli.* This finding suggests that in *S. thermophilus*, OrfG likely adopts a similar orientation, exhibiting an in-out topology with its soluble region positioned on the extracellular side of the cytoplasmic membrane.

#### Examination of OrfG surface accessibility

The topology of OrfG deduced from the dual reporter PhoA-LacZ in *E. coli* prompted us to examine the precise location of the OrfG C-terminal region within the cell wall of *S. thermophilus*. In the following sections, the C-terminal soluble region of OrfG is denoted OrfG_Ext_ (“Ext” for extracellular which corresponds to OrfG_64–331_). We sought to determine whether OrfG_Ext_ was exposed on the bacterial surface or whether it was engulfed within the peptidoglycan layers of *S. thermophilus*. Thus, to examine the surface accessibility of OrfG in *S. thermophilus*, we used the opsonophagocytic assay (OPA) and enzyme-linked immunosorbent assay (ELISA). Initially, terminal sera raised against OrfG_Ext_ were compared with the preimmune sera in different OPAs against the WT strain. As shown in Fig. 2C, both sera exhibited high killing activities against the WT strain at the two tested concentrations. This is likely due to nonspecific antibodies present in the rabbit sera before immunizations, as previously reported for other bacteria (*30*). To confirm this hypothesis, we prepared a preabsorbed (“cleaned”) sera by preincubating terminal and preimmune sera with the strain ∆*orfG* (for more details, see Materials and Methods). The WT strain was killed only by the cleaned terminal sera not by the absorbed preimmune sera, indicating that the killing is mediated by the antibodies produced against OrfG_Ext_ (Fig. 2C). To confirm these results, OPA experiments were conducted using affinity-purified Ab-G. As expected, opsonophagocytic experiments exhibited high killing activities correlated with antibody concentration (Fig. 2D). Subsequently, opsonophagocytic inhibition experiments [opsonophagocytic inhibition assay (OPIA)] were conducted to determine whether cell death was caused by antibodies directed against the OrfG located on the surface of *S. thermophilus*. In these experiments, Ab-G were preincubated with purified OrfG_Ext_ (considered hereafter as the inhibitor) before being added to the OPA mixture. As depicted in Fig. 2E, preincubation of Ab-G with OrfG_Ext_ significantly inhibited opsonic killing. This effect was directly proportional to the amount of inhibitor, and killing was observed at the lowest inhibitor concentration. The findings of both OPA and OPIA were further confirmed through whole-cell ELISA assays performed on WT or ∆*orfG* strains (Fig. 2F). Together these results demonstrate that OrfG exposes its C-terminal soluble region (i.e., OrfG_Ext_) on the surface of *S. thermophilus* rather than being embedded in the cell wall matrix (Fig. 2G).

### Involvement of D1, D2, and TMDs in OrfG oligomerization

#### Analysis of OrfG multimerization using BACTH

**Fig. 3.**
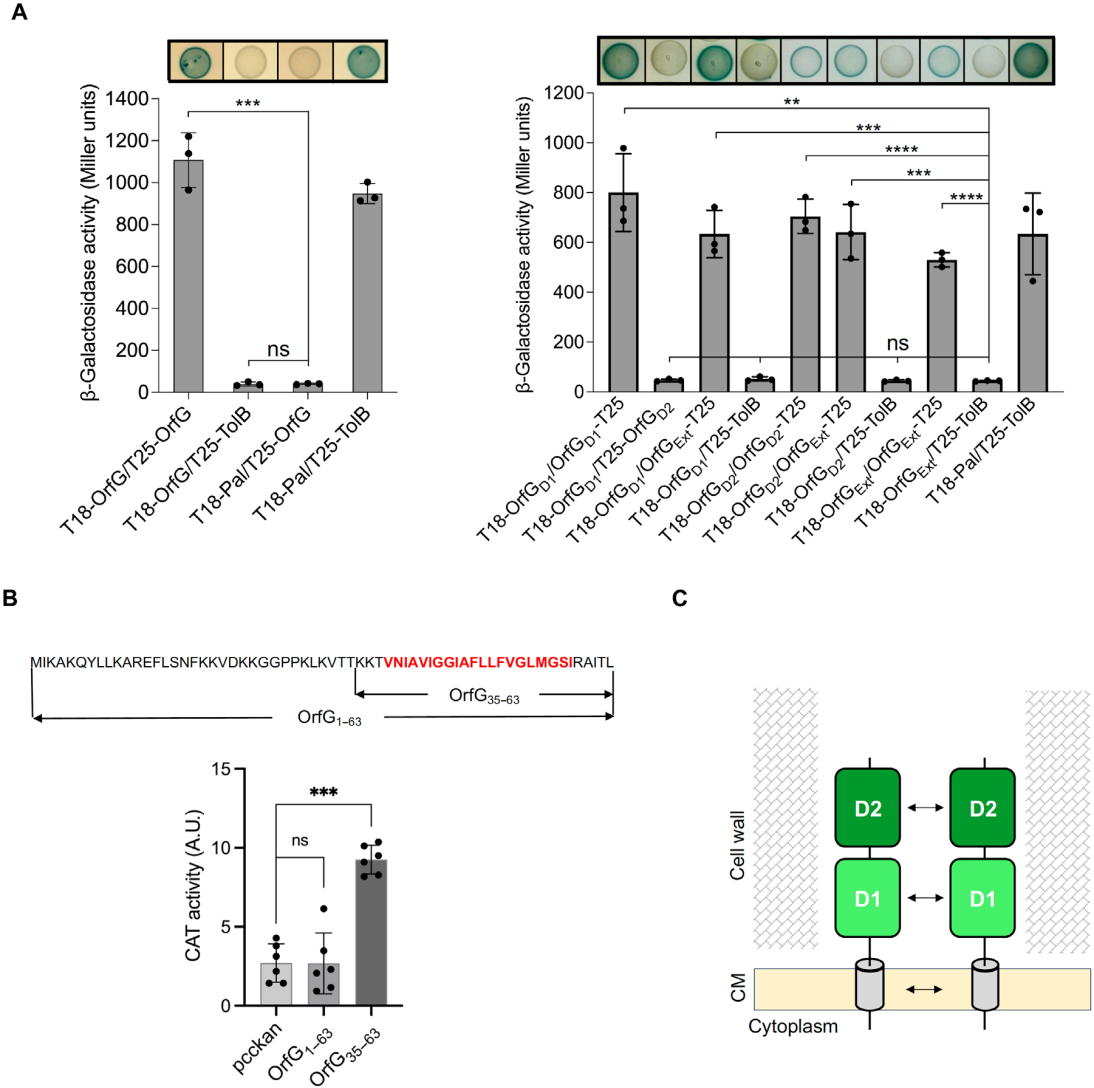
Role of D1, D2, and TMDs in OrfG multimerization. (**A**) Vectors encoding the full-length OrfG or its domains fused to the N or C terminus of T18 or T25 domains were used for BACTH assays. Interactions were assessed through β-galactosidase activity and phenotypic assay using LB plates supplemented with the chromogenic substrate X-Gal. Positive interactions were easily identified by blue colonies of the transformed strains. OrfG_D1_, OrfG_D2_, and OrfG_Ext_ represent the regions of OrfG corresponding to OrfG_64–215_, OrfG_223–331_, and OrfG_64–331_, respectively. The original plate displaying all tested interactions is shown in fig. S1B. (**B**) TOXCAT analysis of OrfG multimerization via its TMD. Top: Graphical representation of OrfG variants used for TOXCAT assay. The OrfG_1–63_ variant corresponds to the whole N-terminal region including a short cytoplasmic extension and the TM α helix (residues 38 to 58 in red). Bottom: Quantitative CAT activity of NT326 reporter cells producing ToxR-X-MalE fusions [X corresponds to the region between ToxR and MalE encoded by the empty vector (pccKAN) or encoding for OrfG_1-63_ or OrfG_35–63_]. Bars and whiskers denote mean values and SEM; not significant (*P* ≥ 0.05), *P < 0 .05, **P < 0.01, ***P < 0.001, and ****P < 0.0001. (**C**) Schematic representation indicating the different interacting domains of OrfG. CM: cytoplasmic membrane. A.U., arbitrary units.

The precise role of VirB8-like proteins in T4SS biogenesis and function remains somewhat elusive. Nonetheless, it has been demonstrated that VirB8-like multimerization was important for T4SS function (*22*). In a previous study, using various in vitro approaches, we showed that OrfG_Ext_, as well as its both individual D1 and D2 subdomains, could assemble into multimers in solution despite predominantly existing in a monomeric form (*28*). To investigate the self-interaction of OrfG and to identify the key domains involved in its multimerization, we used a bacterial two-hybrid (BACTH) assay based on the reconstitution of the complementary T18 and T25 domains of the *Bordetella pertussis* adenylate cyclase (*31*). BACTH data showed that full-length OrfG and OrfG_Ext_ multimerize in vivo (Fig. 3A, left, and fig. S1, B and C). We also demonstrated that D1 and D2 self-interact, but no cross-interaction could be detected between them (Fig. 3A, right, and fig. S1, B and C).

#### Examining OrfG TMD self-interaction using TOXCAT

To assess the role of OrfG TMD in OrfG oligomerization, we used the TOXCAT assay, a two-hybrid genetic tool developed for monitoring homotypic interactions between TMDs. This was achieved by using chloramphenicol disk assays or by measuring the chloramphenicol acetyltransferase (CAT) activity (for more details, see Materials and Methods) (*32*). Referring to the proposed topology of OrfG (Fig. 2G), two truncated variants were generated, OrfG_1–63_ and OrfG_35–63_ (Fig. 3B, top). The shortest variant OrfG_35–63_, containing only the predicted TMD, exhibited the highest CAT activity (Fig. 3B, bottom). The absence of CAT activity for OrfG_1–63_ variant may be attributed to the presence of predicted secondary structures at its N-terminal end (fig. S1D), which could confer rigidity and hinder ToxR dimerization. Combining the results from BACTH and TOXCAT assays, we demonstrate that OrfG multimerization involves its D1, D2, and TMDs (Fig. 3C).

### The crystal structure of OrfG_Ext_ reveals a trimeric arrangement

#### The trimeric assembly observed in the crystal

**Fig. 4.**
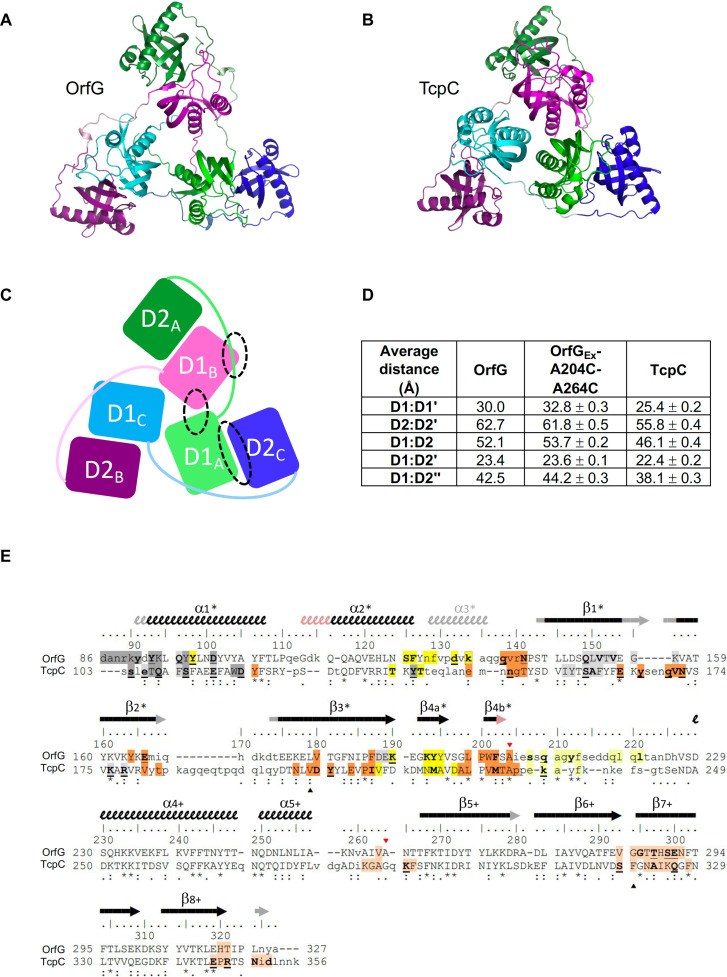
Structural comparison of OrfG and TcpC. (**A** and **B**) Cartoon views of the trimers of OrfG_Ext_ (this work, PDB entry 7pkw) and TcpC (3ub1), respectively. (**C**) Schematic representation of the trimeric organization of OrfG_Ext_ and the way one monomer interacts with the two others. (**D**) Table of distances observed between domains in the trimers of OrfG_Ext_, OrfG_Ext_-A204C-A264C, and TcpC. D1 and D2 are for the domains of the same monomer, a prime is used for the domain of the neighboring monomer, and a double prime is used for the third monomer. For instance, D1:D2′ represents D1_A_:D2_C_, D1_B_:D2_A_, or D1_C_:D2_B_ for OrfG and equivalent pairs in TcpC. (**E**) Structure-based sequence alignment of OrfG_Ext_ and TcpC generated by mTM-align and refined manually. Upper/lowercase letters distinguish residues that are or are not structurally superimposed. Numbering above the sequences corresponds to OrfG sequence. Secondary structures are represented by arrows (β strands) and squiggles (α helices), in black when shared by OrfG and TcpC, in light orange when only present in OrfG, and in gray when only present in TcpC. Their names are followed by a star (or a cross) when they belong to the domain D1 (or D2, respectively). Residues found at a distance of less than 4 Å in trimeric assemblies are highlighted: Gray corresponds to the D1:D1′ interface (dark gray for D1, light gray in the neighboring D1′), yellow is for the interactions between the linker L_12_ (light yellow) that connects D1 to D2 and the domain D1′ (dark yellow), and orange corresponds to the interface formed between D1 (dark orange) and D2′ (light orange). Residues that form hydrogen bonds are shown in bold type and are underlined if their side chain is involved. Interactions mediated through water molecules were not considered. The triangles above/below the sequences correspond to residues that were mutated to cysteines to trap the trimers (red: OrfG, black: TcpC).

We determined the crystallographic structure of OrfG_Ext_ at a resolution of 1.83 Å (Fig. 4A and table S1). As anticipated, it exhibits two NTF2-like domains D1 and D2, connected by a long-extended linker. In domain D1, the region between residues 92 and 204 forms two successive α helices followed by a long loop (instead of a third α helix typically observed in VirB8 and VirB8-like proteins) and then a highly curved antiparallel β sheet composed of four strands wrapped around the first α helix. The domain D2 (residues 228 to 321) adopts a similar fold to domain D1 [root mean square deviation (RMSD) = 2.57 Å for 78 aligned residues] but with a shorter α helix 2 and shorter loops between the strands and once again lacks a third α helix before entering the β sheet (Fig. 4A). A notable feature common to these two domains is the high proportion of aromatic residues in their core, predominantly phenylalanines and tyrosines (D1: four Phe + four Tyr; D2: seven Phe + two Tyr), at the interface between the helix 1 and the β sheet. In D1, additional tyrosines are located on the outer side of the helix and at its entrance, exposed to the solvent. This represents a total of seven tyrosines between residues Y91 and Y106, i.e., more than 40% tyrosines in this 16-residue sequence.

In the crystal, OrfG_Ext_ forms a complex consisting of three intertwined protomers related by a threefold axis (Fig. 4A). The six NTF2-like domains are arranged in an almost flat triangle, with three domains D1 in the center and three domains D2 at the vertices. The notable feature of this arrangement is that each domain D1 not only faces the others (distance from center to center: 30 Å) but interacts with the domain D2 of the neighboring monomer (at 24 Å) instead of its own one positioned far away (52 Å), thanks to the long linker that stretches to wrap around the domain D1 of that neighbor (Fig. 4D).

By searching the Protein Data Bank (PDB) for homologous structures using DALI (*33*), we identified TcpC, the VirB8-like from pCW3 (Fig. 1) (PDB entry 3ub1) as the best and only model that resembled OrfG across the entire length of the solved structure (Fig. 4B). The structural neighbors that ranked with lower scores corresponded to single domains. D1 exhibits similarity to other VirB8 and VirB8-like domains, as we previously described (*28*). However, D2 appears more original, as its structural neighbors, other than TcpC, comprise a diverse list of proteins or enzymes found in organisms of various kingdoms.

#### OrfG and TcpC share the same trimeric assembly

To date, TcpC is the only known structure that resembles OrfG for both D1 and D2. Notably, this similarity also extends to the quaternary structure, as TcpC forms trimers characterized by the same interwined arrangement as OrfG (Fig. 4, A and B). The overall superimposition of the trimers yields an RMSD of 3.4 Å for 555 aligned Cα atoms. However, this value is relatively poor due to the tighter fit of TcpC around the threefold axis compared to OrfG. All interdomain distances are at least 10% smaller in TcpC (Fig. 4D and table S2), except for the distance between domain D1 and its closest neighbor D2 from another monomer (hereafter named D1:D2′, where the prime symbol denotes the adjacent protomer in the trimeric complex), which remains almost constant: 22.5 Å in TcpC and 23.8 Å in OrfG. Similarly, the TM score, a better criterion than RMSD for comparing structures (*34*), underscores the significance of the interaction D1:D2′ (table S2). Analysis using PDBePISA (*35*) highlights the distinctiveness of this D1:D2′ pair. The D1 domains show minimal interaction (273 Å^2^ in OrfG, 444 Å^2^ in TcpC) and do not significantly contribute to the trimer stability, while the D1:D2′ interface represents a sufficient contribution to maintain the assembly (910 Å^2^ in OrfG, 965 Å^2^ in TcpC). Porter and colleagues (*23*) extensively reviewed this interface in TcpC. In OrfG, the same region is implicated, albeit with minimal amino acid conservation (Fig. 4, C and E). This observation, initially disappointing, may instead provide additional evidence for the existence of these trimeric forms, which persist despite the low sequence identity between the OrfG and TcpC soluble domains (12%) (Fig. 4E). In the trimeric assembly of OrfG and TcpC, the D1:D1' and D1:D2' domain associations are unique to these two proteins, not being found in other VirB8-like proteins [except for the D1:D1′ in TraM from pIP501 (*24*)], nor in any VirB8 from Gram-negative systems (fig. S2, A and B). The variation in domain associations observed in a wide variety of VirB8-like/VirB8 assemblies appears to be a distinctive property of this family of proteins (*22*).

### OrfG forms trimers in vitro and in vivo

#### Interrogation of trimer formation using site-specific cysteine incorporation

**Fig. 5.**
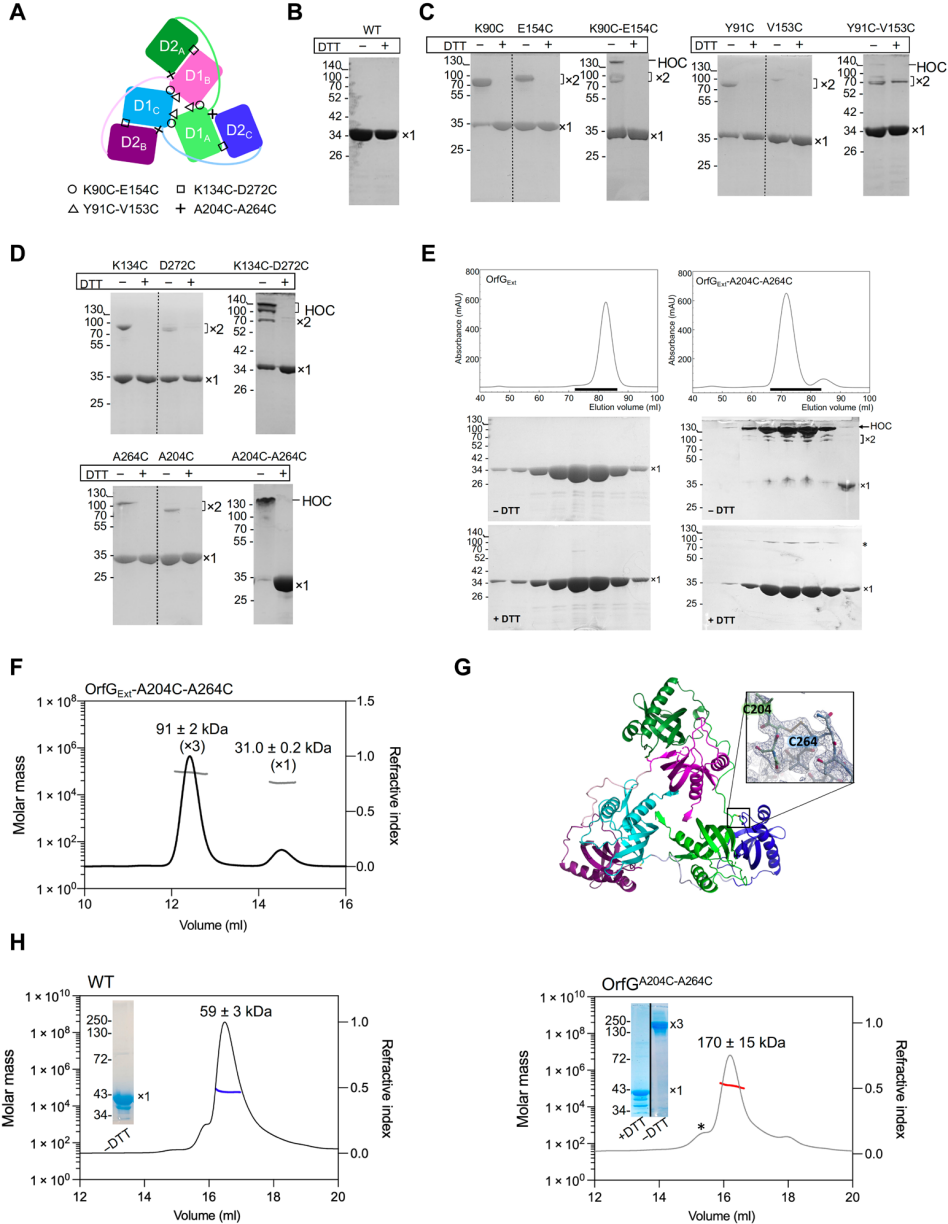
OrfG_Ext_ and the full-length OrfG both form trimers in vitro. (**A**) Schematic representation of the OrfG_Ext_ trimer. Target residues for cysteine cross-linking are highlighted in circle, square, triangle, and plus symbols. (**B**) SDS-PAGE analysis of purified OrfG_Ext_ (WT) in the absence or presence of DTT. (**C** and **D**) SDS-PAGE analysis of OrfG_Ext_ variants harboring simple and double-cysteine mutations in the absence or presence of DTT. The monomeric (×1), dimeric (×2), and the high-order complexes (HOC) observed are indicated on the right of each gel. The intensity of the cross-linked dimers varies in terms of yield and migration profiles. The cross-linking yield varies on the basis of the cysteine positions, while the disparity in migration profile is a common behavior for cysteine variants (*59*, *60*). (**E**) SEC of purified OrfG_Ext_ and OrfG_Ext_-A204C-A264C. Black lines indicate the fractions analyzed by SDS-PAGE in the absence or presence of DTT. Monomeric (×1), dimeric (×2), and the HOC observed in OrfG_Ext_ or OrfG_Ext_-A204C-A264C are indicated on the right of each gel. The asterisk indicates a minor band corresponding to the OrfG_Ext_-A204C-A264C cross-linked complex resistant to the DTT treatment. mAU, milli-absorbance unit. (**F**) SEC-MALS analysis of OrfG_Ext_-A204C-A264C. The elution profile (black line) is shown with the molecular weight calculated by MALS (gray lines). The elution volume (milliliters) is plotted on the first *y* axis, and the refractive index is plotted on the second *y* axis. (**G**) Cartoon presentation of the crystal structure of OrfG_Ext_-A204C-A264C. Each monomer is presented with a distinct color. The disulfide bridges are shown as sticks, and the insert details the model in the region of Cys204_A_-Cys264_C_, with the corresponding 2Fo-Fc electron density map contoured at 1.2σ. (**H**) Analysis of purified full-length OrfG (WT) and the A204C-A264C variant (OrfG^A204C-A264C^) using SEC-MALS. Fractions picked from the center of each elution peak were analyzed in the absence or presence of DTT as indicated in each inset. The average molecular weights are shown on the top of each peak. The calculated molecular weights included the theoretical molecular weight of full-length OrfG (39 or 117 kDa which corresponds respectively to the theoretical molecular weights of OrfG monomer and trimer) and the unknown molecular weight of OGNG micelles (reported to have a variable molecular weight). The SEC profile of OrfG^A204C-A264C^ shows a minor early peak eluted between 15 and 15.5 ml (indicated by an asterisk) with an average molecular weight of 398 ± 77 kDa. All molecular weights indicated at the top of each peak represent the average molecular weight and SD from three independent measurements (except the monomeric form of OrfG_Ext_-A204C-A264C which was observed only once in three independent experiments).

Both OrfG and TcpC were observed predominantly as monomers in solution (*23*, *28*). However, their crystal structures revealed a similar trimeric arrangement (Fig. 4, A and B). This finding led us to hypothesize that trimers represent the functional packing of these proteins. We postulate that trimers do assemble in solution but are not sufficiently stable to be detected. To confirm this hypothesis, we used a cysteine cross-linking approach to investigate the ability of OrfG_Ext_ to form trimers in vitro. We introduced cysteines to replace facing residues at the interface D1:D1′ (K90C-E154C and Y91C-V153C) or at the interface D1:D2′ (K134C-D272C and A204C-A264C) (Fig. 5A). All OrfG_Ext_ variants with single or double-cysteine substitutions were purified, and their cross-linking products were analyzed in the presence and absence of the reducing agent dithiothreitol (DTT). In contrast to the WT OrfG_Ext_ which migrated as a monomer (Fig. 5B), all variants harboring single-cysteine mutations formed homodimers in the absence of DTT (Fig. 5, C and D). On the basis of the arrangement of OrfG protomers in the trimeric complex, we believe that these cross-linked dimers correspond to artifactually formed complexes. Notably, under nonreducing conditions, variants with double-cysteine mutations, particularly OrfG_Ext_-A204C-A264C, assemble into high-order complexes (HOC) (>100 kDa), possibly corresponding to trimeric form (theoretical molecular weight of 93 kDa) (Fig. 5D). When we further investigated purified variants OrfG_Ext_-A204C-A264C, OrfG_Ext_-A204C, and OrfG_Ext_-A264C by size exclusion chromatography (SEC) coupled to multiangle light scattering (MALS), we found that OrfG_Ext_-A204-A264C assembles into a larger complex compared to the cysteine single variants and assemble into trimers (Fig. 5, E and F, and fig. S3, A and B). To confirm that the trimeric complex obtained using cysteine cross-linking conserved the same structural arrangement as OrfG_Ext_, we solved the structure of OrfG_Ext_-A204C-A264C at 2.6-Å resolution. As depicted in Fig. 5G, OrfG_Ext_-A204C-A264C shares all the characteristics of the WT structure. Despite various measurements carried out to characterize the trimers and comparing them, we observed little adaptation to the constraints brought by the disulfide bridges and/or the change of the crystallization conditions, the overall arrangement remains unchanged, as anticipated (Fig. 5G and tables S1 and S2).

#### Full-length OrfG forms trimers

Both full-length OrfG and the variant harboring the double-cysteine substitutions A204C/A264C (OrfG-A204C-A264C) were overexpressed and extracted from *E. coli* membranes in the presence of detergents. SEC-MALS analysis conducted on both purified proteins revealed that OrfG-A204C-A264C mainly assembles into trimers (Fig. 5H, right), in contrast to the full-length OrfG, found as monomers (Fig. 5H, left).

#### In vivo existence and functionality of full-length OrfG trimers

**Fig. 6.**
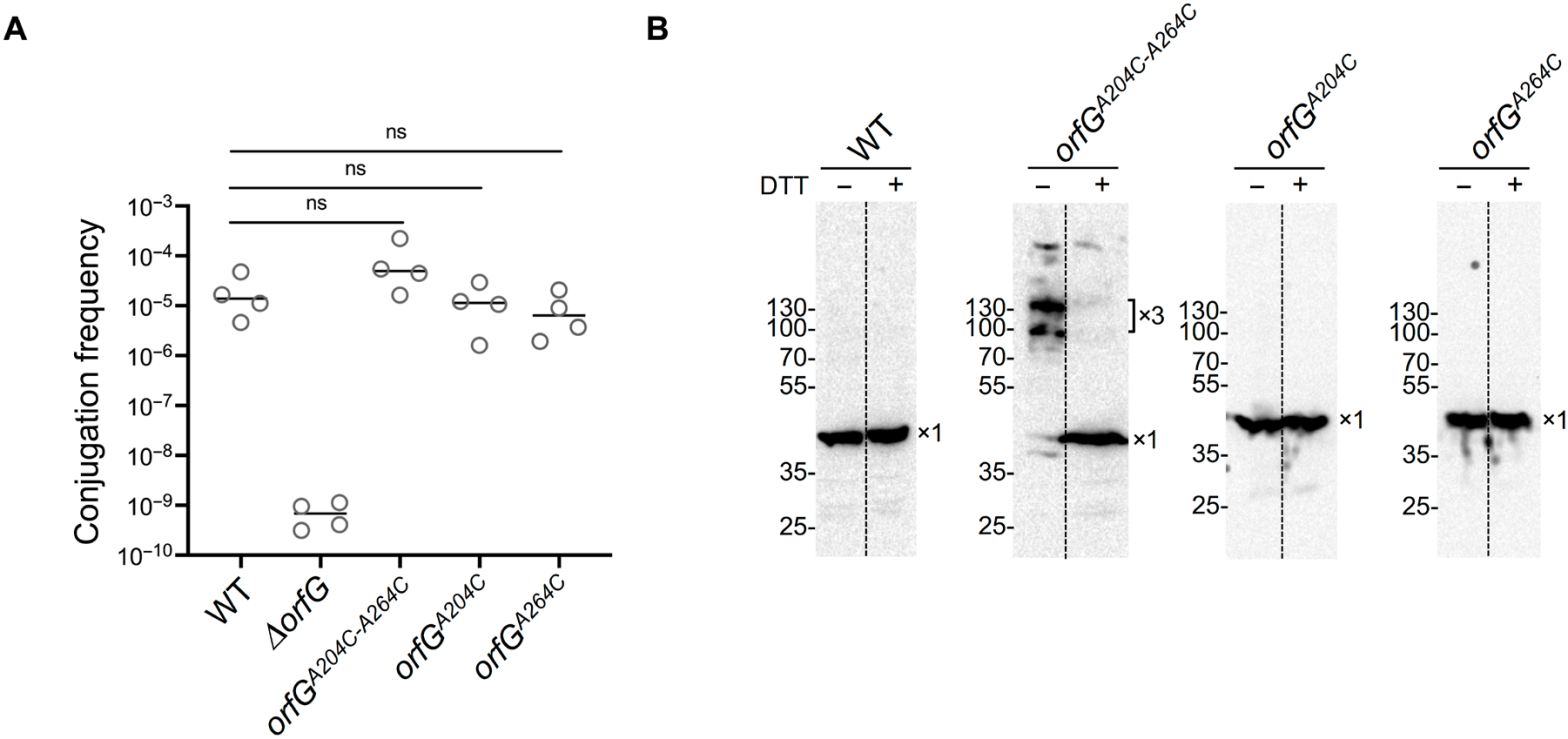
In vivo cysteine cross-linking and functionality of OrfG cysteine variants. (**A**) Conjugation frequencies of the WT strain and ∆*orfG* strain as well as the strains producing OrfG-A204C-A264C (*orfG^A204C-A264C^*), OrfG-A204C (*orfG^A204C^*), and OrfG-A264C (*orfG^A264C^*). Transfer frequency is expressed as the number of transconjugants per donor cells. The mean of four biological replicates is shown. (**B**) Polyclonal Ab-G was used for the immunoblotting of membrane fractions separated from whole-cell lysate of cells producing the WT, *orfG^A204C-A264C^*, *orfG^A204C^*, and *orfG^A264C^*. The membrane fractions were analyzed in the absence or presence of DTT. Western blot analysis of *orfG^A204C-A264C^* variant shows the presence of two bands with an apparent molecular weights of 130 kDa (upper band) and 100 kDa (lower band). The upper band has a molecular weight slightly higher than the theoretical molecular weight of OrfG trimer (120 kDa). This aberrant migration is consistent with observations of cysteine cross-linked proteins particularly membrane proteins, reported in previous studies (*61*–*63*). The nature of the second band remains unclear. Since no dimers were observed in the OrfG^A204C^ or OrfG^A264C^, we suggest that this lower band likely represents a more compact trimeric form of OrfG^A204C-A264C^ variant. Bands corresponding to monomeric (×1) and trimeric (×3) OrfG are indicated on the right. Bars denote mean values.

To gain further insights into the assembly mechanism of OrfG, we extended our investigations to the in vivo environment. First, we tested the functional requirement of OrfG for the conjugative transfer of ICE*St3*. Mating experiments conducted with the Δ*orfG* strain revealed that OrfG was essential for ICE*St3* conjugation and likely for the T4SS function as well (Fig. 6A). To test the potential of OrfG to function as trimer in a biological context, a *orfG* knockout mutant strain producing a full-length OrfG harboring the dual cysteine substitutions (A204C and A264C) was generated and subsequently examined for the presence of multimeric complexes. Mating experiments demonstrated that the strain producing the dual cysteine mutant conjugates at frequencies comparable to that of the WT strain (Fig. 6A). Cell fractionation followed by Western blot analysis of the total, the cytoplasmic and the membrane fractions, confirmed the production of full-length OrfG-A204C-A264C variant and its proper localization within the membrane fractions (fig. S4). To assess the multimeric state of OrfG-A204C-A264C, the membrane fractions were subjected to Western blot analyses in the absence or presence of DTT (Fig. 6B). In the absence of DTT, the OrfG-A204C-A2064C membrane sample displayed high molecular weight complexes including one of approximatively 130 kDa, consistent with the expected size of OrfG trimers (40 kDa × 3 = 120 kDa). Next, we confirmed that this OrfG trimeric complex resulted from the cross-linking of the inserted artificial cysteines by observing its dissociation upon treatment with DTT. Further confirmation was provided through the analysis of the strain producing OrfG with the single-cysteine substitutions A204C or A264C. In addition to their ability to complement the Δ*orfG* strain in conjugation assays, both single-cysteine variants were shown to be correctly localized in bacterial membranes and did not display cross-linked complexes in the absence of DTT (Fig. 6, A and B, and fig. S4).

### Despite their high sequence diversity, VirB8-like proteins exhibit a common assembly mode

#### Diversity of VirB8-like proteins within MPF_FA_ clade

**Fig. 7.**
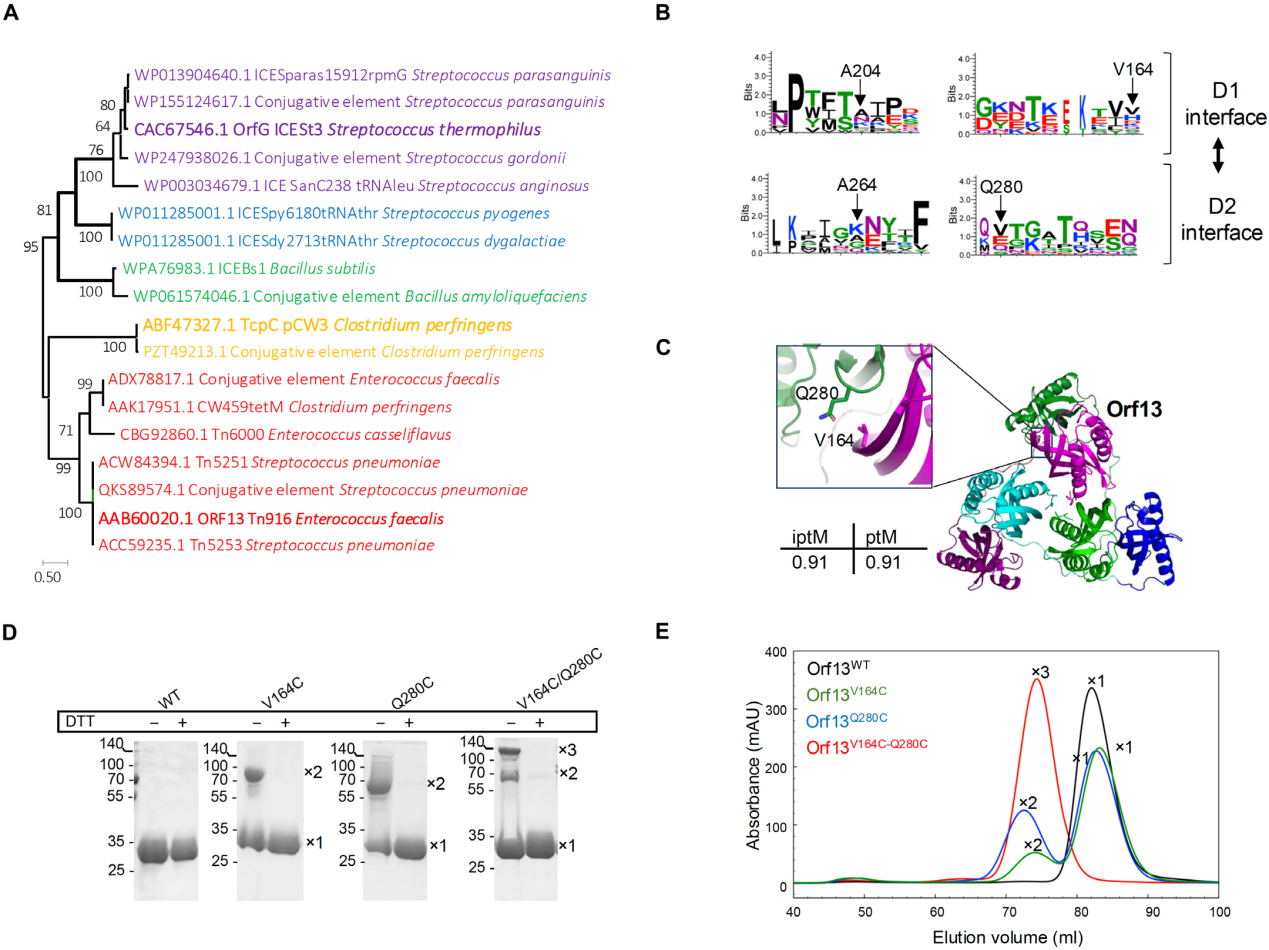
The trimeric assembly of VirB8-like proteins is conserved among the β-class. (**A**) Phylogenetic analysis of VirB8-like proteins belonging to the β-class. The tree was built with MEGA11 using maximum likelihood. The branch support of the groupings was estimated using bootstrap (100 replicates). The five distinct families of VirB8-like proteins from the β-class are presented in different colors. (**B**) Sequence comparisons from the trimeric binding interfaces D1:D2′ were presented by sequence logos using WebLogo 3 (*64*). The positions of the residues A204/A264 of OrfG and V164/Q280 of Orf13 used to design the cysteine variants were illustrated on the sequence logos. (**C**) Cartoon presentation of the AlphaFold 3 model of Orf13. Each monomer is presented with a distinct color. A zoom-in on the D1:D2′ interface on the AlphaFold 3 model of Orf13 highlights the residues V164 and Q280 targeted by cysteine mutation. Confidence scores, including the predicted TM score (pTM) and interface pTM score (ipTM) provided by AlphaFold multimer for the top ranked model, are listed alongside Orf13 model. (**D**) SDS-PAGE analysis of purified Orf13 (WT) and Orf13 cysteine variants harboring simple and double-cysteine mutations in the absence or presence of DTT. The monomeric (×1), dimeric (×2), and trimeric (×3) forms observed are indicated on the right of each gel. (**E**) SEC of purified Orf13^WT^ and its cysteine variants. The elution volume (milliliters) is plotted on the first *y* axis, and the ultraviolet absorbance (mAU) is plotted on the second *y* axis. The monomeric (×1), dimeric (×2), and trimeric (x3) forms identified by SEC-MALS are indicated on each peak.

Despite a notable divergence in their amino acid sequences, both OrfG from ICE*St3* and TcpC from pCW3 adopt a trimeric structure, suggesting that trimeric assembly would be common among VirB8-like proteins from β-class. To evaluate their sequences diversity, we used Tcoffee (*36*) to align sequences of a large set of VirB8-like proteins from MPF_FA_ found in Gram-positive plasmids and ICEs. Among the 18 analyzed sequences, a high degree of diversity was observed, enabling us to classify the VirB8-like proteins into five distinct families. Each family contains members sharing a minimum of 40% sequence identity (fig. S5). Subsequently, we generated a phylogenetic tree to depict the relationships between these distinct families (Fig. 7A). OrfG and TcpC were affiliated to two distinct families, reflecting their significant evolutionary divergence. This sequence diversity also extends to the contact interfaces involved in trimeric assembly of OrfG and TcpC (Fig. 7B and fig. S6A) and other VirB8-like proteins as suggested by their AlphaFold 3 (*37*) models (fig. S6B). Despite the fine analysis of the binding interfaces, we could not identify a specific pattern that could indicate how the trimer formation occurs.

#### Trimeric assembly of the Tn916 VirB8-like protein

To explore the ubiquity of the trimeric assembly within VirB8-like proteins from β-class, our investigation was focused on Orf13, the VirB8-like component of Tn*916* (Fig. 1). Our selection was motivated by several reasons: (i) Tn*916* is the canonical element of the Tn*916*/ICE*Bs1*/ICE*St3* superfamily, widespread in Gram-positive bacteria; (ii) Orf13 is predicted to be the unique VirB8-like protein encoded by the Tn*916* conjugation module (*38*); and (iii) it presents a low sequence identity with OrfG (20%) (fig. S6A). The three-dimensional model of the Orf13 soluble domain (residues 71 to 304, named hereafter Orf13^WT^) generated by AlphaFold 3 (*37*) proposes that Orf13^WT^ comprises two NTF2-like domains, D1 (residues 71 to 184) and D2 (residues 209 to 304) adopting the same arrangement as observed in the crystal structures of OrfG and TcpC (Fig. 7C). To provide experimental data on the trimeric assembly of Orf13, we performed cysteine substitutions followed by disulfide cross-linking analysis. We selected two facing residues at the interface D1:D2′ (V164 and Q280) for the substitution to cysteines (Fig. 7C). Single- and double-cysteine variants of the Orf13^WT^ were produced and analyzed by SDS–polyacrylamide gel electrophoresis (SDS-PAGE) in the absence or in the presence of DTT. The detection of high molecular weight complexes (above 100 kDa) in the Orf13-V164-Q280C variant (Fig. 7D) absent in both single variants corroborates the hypothesis of a trimeric assembly of Orf13. Furthermore, a trimeric complex was also detected in SEC-MALS assays for the double-cysteine variant V164C-Q280C, while Orf13^WT^ as well as the two single-cysteine variants displayed monomeric or dimeric forms (Fig. 7E and fig. S7). These results support the proposed model in which Orf13 from Tn*916* adopts a trimeric structure, an arrangement conserved among VirB8-like proteins from β-class, despite their substantial sequence diversity.

### Mapping of OrfG interaction network within predicted T4SS subunits

**Fig. 8.**
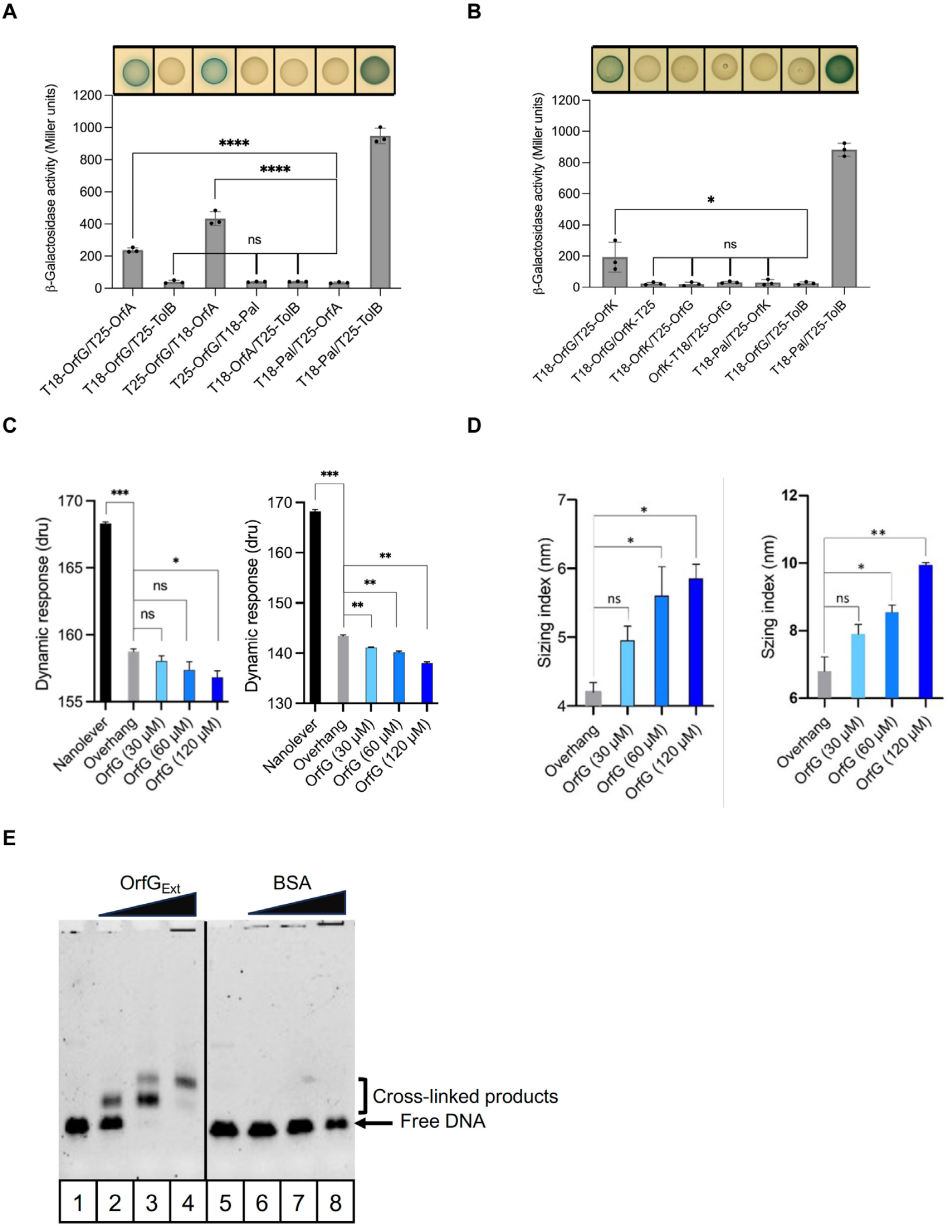
Charting the interaction network of OrfG. (**A**) Identification of OrfG-OrfA and (**B**) OrfG-OrfK interactions using BACTH. Colonies producing the hybrid proteins from the LB plates supplemented with X-Gal and their corresponding β-galactosidase activities are shown. (**C**) Conformation and (**D**) sizing analysis of OrfG binding to DNA [with ssDNA (in left) and dsDNA (in right)]. The results are represented by plotting the used analyte [purified recombined OrfG_Ext_ (OrfG) or controls (Nanolevers or/and Overhang)] in the *x* axis and the dynamic response unit (dru) and sizing index in the *y* axis. Statistical significance was performed by the *t* test using GraphPad Prism (nonparametric test, followed by the impaired test). Bars and whiskers denote mean values and SEM; not significant (*P* ≥ 0.05), *P < 0.05, **P < 0.01, ***P < 0.001, and ****P < 0.0001. Statistical analysis was made between Nanolever (black) and Overhang (gray) for conformational analysis in (C) and then between Overhang to that with the presence of increasing concentration of OrfG_Ext_ (blue degraded) for both of conformational (C) and sizing analysis (D). (**E**) In vitro chemical cross-link of OrfG_Ext_ to ssDNA. Electrophoretic mobility shift assay (EMSA) analysis of 1 μM ssDNA* (listed in table S4) with 2.5% of PFA in the presence of increasing quantities of purified OrfG_Ext_ and the BSA (negative control). The quantities of OrfG_Ext_ and BSA used for each reaction are 0 μg for columns 1 and 5, 0.2 μg for columns 2 and 6, 0.4 μg for columns 3 and 7, and 0.6 μg for columns 4 and 8. Free DNA and identified complex stabilized by PFA cross-linking are indicated at the left of the gel. BSA, bovine serum albumin. Bars and whiskers denote mean values and SEM; not significant (*P* ≥ 0.05), *P < 0.05, **P < 0.01, ***P < 0.001, and ****P < 0.0001.

The conjugation module of ICE*St3* carries genes encoding for 14 proteins named OrfA to OrfN. Six of them are consistently found within MPF_FA_ clade and encode for OrfA, OrfC, OrfD, OrfE, OrfF, and OrfK proteins (Fig. 1). Our in silico analysis using CCTOP (*27*) revealed that OrfA, OrfC, OrfE, OrfF, and OrfK are predicted to be transmembrane proteins. To search for potential proteins interacting with OrfG, we used the BACTH technique, which was adapted to examine interactions between membrane proteins. OrfG was found to interact with OrfA and OrfK, which was the predicted peptidoglycan hydrolase (VirB1-like) (Fig. 8A) and the coupling protein of the system (Fig. 8B), respectively. However, no interaction could be detected with the remaining proteins (fig. S8).

### Exploring the interaction between OrfG and DNA

To understand more precisely the role of OrfG in the conjugation process, particularly its involvement in DNA translocation, we studied the interaction of OrfG with ICE*St3* DNA using the SwithSENSE technology (*39*). The interaction was monitored with single-strand DNA (ssDNA) or double-strand (dsDNA) fragments referred here as Overhangs (fig. S9A and table S4). Addition of OrfG at increasing concentrations onto the Nanolever functionalized with Overhangs DNA sequences showed that the presence of OrfG significantly reduced the dynamic response. This indicates that OrfG contributes to the reduction of the movement of the Nanolever fixed on the sensor surface, indicating a direct binding of OrfG to both Overhangs DNA sequences. These results were comforted by quantifying the dynamic response and the apparent hydrodynamic diameter of the Nanolever (sizing index) alone or in the presence of OrfG. As depicted in Fig. 8 (C and D), both the dynamic response and the sizing index showed increased values upon the injection of OrfG compared to the Nanolever alone. Similar results were obtained with ssDNA (fig. S9B) and dsDNA (fig. S9C) fragments not derived from ICE*St3*, suggesting a nonspecific nature of the interaction between OrfG and DNA.

To capture potential complexes occurring between OrfG and DNA, in vitro cross-linking assays were conducted with paraformaldehyde (PFA). By using the identical DNA fragments used in the switchSENSE experiments, we observed that increasing concentration of OrfG led to the formation of PFA-stabilized OrfG-DNA complexes, in contrast to the negative control using bovine serum albumin (BSA; Fig. 8E). Together, these findings suggest that OrfG would bind DNA in a nonspecific and transition manner.

## DISCUSSION

In contrast to numerous structural data obtained for conjugative T4SSs from Gram-negative bacteria (*12*, *15*, *16*, *18*), no such structures have been obtained for Gram-positive systems, hampering our understanding of their assembly and functioning. In Gram-positive bacteria, VirB8-like proteins are recognized to be essential for the conjugation process and act as core components of T4SSs (*22*–*25*). Nevertheless, their precise role in T4SSs biology remains fully unknown. This study focuses on elucidating how VirB8-like proteins assemble and how they participate in the DNA transfer process.

VirB8-like proteins belong to the VirB8 family, well-characterized in Gram-negative T4SSs (*26*). VirB8 are small proteins comprising a short N-terminal cytoplasmic segment, a TMD, and a single periplasmic domain adopting an NTF2-like globular fold (*26*). They all belong to the α-class proposed by Goessweiner-Mohr and colleagues (*24*, *25*). In Gram-positive T4SSs, VirB8-like proteins exhibit much greater diversity in terms of domain composition. They are categorized into three classes, the α-class includes members resembling to VirB8s from Gram-negative systems and two additional classes called β and γ (*40*). VirB8-like proteins of the β-class have a single TMD followed by two NTF2-like domains, while those of the γ-class comprise two globular domains, including one NTF2-like domain, separated by a TMD. On the basis of phylogenetic analysis of key conjugation proteins, bacterial T4SSs have been divided into eight clades, two of them have been found in Gram-positive bacteria, MPF_FA_ and MPF_FATA_ (*20*). We observed that VirB8-like classes were distributed accordingly in the different clades, with the β-class associated to MPF_FA_ and the α- and γ-classes to MPF_FATA_ (Fig. 1). This suggests that VirB8-like proteins would evolve with the specific T4SS apparatus they are associated with.

Since our working protein model OrfG, encoded by ICE*St3*, belongs to the β-class, we focused our research on this class of proteins. Cellular topology analysis revealed that OrfG was associated with the membrane fraction, consistent with its transmembrane prediction. More unexpectedly, findings from OPAs indicate that OrfG may not be restricted to the periplasmic space of *S. thermophilus* cell wall but rather extends outward onto the bacterial surface which is likely dependent on the action of the peptidoglycan hydrolase associated to the system. This topology was also observed for other VirB8-like proteins, PrgL from pCF10 (*22*) and TraM from pIP501 (*24*) which belong to α- and γ-classes, respectively. This topology appears to be a common feature of VirB8-like and VirB8 proteins, as they extend their NTF2-like domains outward from the cytoplasmic membrane. However, VirB8-like and VirB8 proteins differ in their cellular localization, with VirB8-like proteins being predominantly extracellular components, while VirB8 from Gram-negative systems are typically periplasmic.

Next, we examined the self-assembly of OrfG using BACTH and TOXCAT techniques. We revealed that OrfG multimerizes through all its subdomains encompassing D1, D2, and its TMD. These findings align with previous studies reporting the multimerization of VirB8-like proteins, anticipating that OrfG would function as a multimer in vivo (*22*). To better understand the structural organization of OrfG, we determined its crystal structure and found that it adopts a trimeric packing. We observed that OrfG forms trimers in *S. thermophilus*, supporting ICE*St3* conjugative transfer. This discovery led us to ask whether this is true for other MPF_FA_ systems. Our analysis of sequence conservation within the conserved proteins from MPF_FA_ clade indicates that the sequence diversity extends across all proteins including VirB8-like proteins (fig. S10). The consistent observation of the trimeric assembly in phylogenetic distant VirB8-like proteins, TcpC from pCW3 (*23*) and Orf13 from Tn*916* (this work) (Fig. 8A), indicates that these trimers undoubtedly constitute the fundamental biological unit of these proteins. It is worth mentioning that despite belonging to the other MPF_FATA_ clade, the NTF2-like domain of TraM from pIP510 forms trimers in its crystal structure (*24*). Although TraM is the sole characterized protein from the γ-class, this finding suggests that trimeric assembly of VirB8-like proteins is more widespread in Gram-positive T4SSs.

To improve our understanding of the role of OrfG in the assembly of the T4SS encoded by ICE*St3*, we explored its interaction with other transfer proteins, OrfA, OrfC, OrfD, OrfE, OrfF, and OrfK (Fig. 1). Expectedly, we observed interaction of OrfG with OrfA and OrfK, the predicted VirB1-like and the coupling protein of the system, respectively. These results are consistent with previous reports of interactions between VirB8-like/VirB1-like and VirB8-like/coupling protein in T4SSs from pCW3 and pIP501 and more recently from pCF10 (*23*, *41*, *42*). However, unlike these systems, we did not detect any interaction between OrfG and the VirB6-like protein OrfC or with the VirB4-like protein OrfD. This may be due to inherent limitations of the BACTH methodology where, for example, fusion proteins might obstruct interaction sites. Alternatively, it is plausible that interactions between OrfG and remaining transfer proteins either do not exist in our system or are too transient to be detected with BACTH.

Because the conjugation process implies the transport of DNA through the T4SS apparatus, we explored whether OrfG was able to interact with DNA. The interaction between DNA and T4SS subunits was principally investigated in vivo using TraIP approach (*43*, *44*). In Gram-negative systems, this approach enabled identification of T4SS subunits capable of making contact with DNA during the conjugation process (*43*). It also defined the subunits of the tunnel through which DNA traverses the T4SS apparatus. In our study, switchSENSE assays, confirmed by chemical cross-linking, allowed us to detect a direct interaction between OrfG and ICE*St3* DNA. This interaction appears to be nonspecific and transient, consistent with the operational mode of T4SSs, which is believed to facilitate a smooth and rapid passage of DNA during the conjugation process (*5*). This finding is of considerable importance for understanding the role of VirB8-like proteins in the functioning of Gram-positive T4SSs, as it suggests that VirB8-like proteins could potentially be an integral part of the DNA translocation tunnel.

**Fig. 9.**
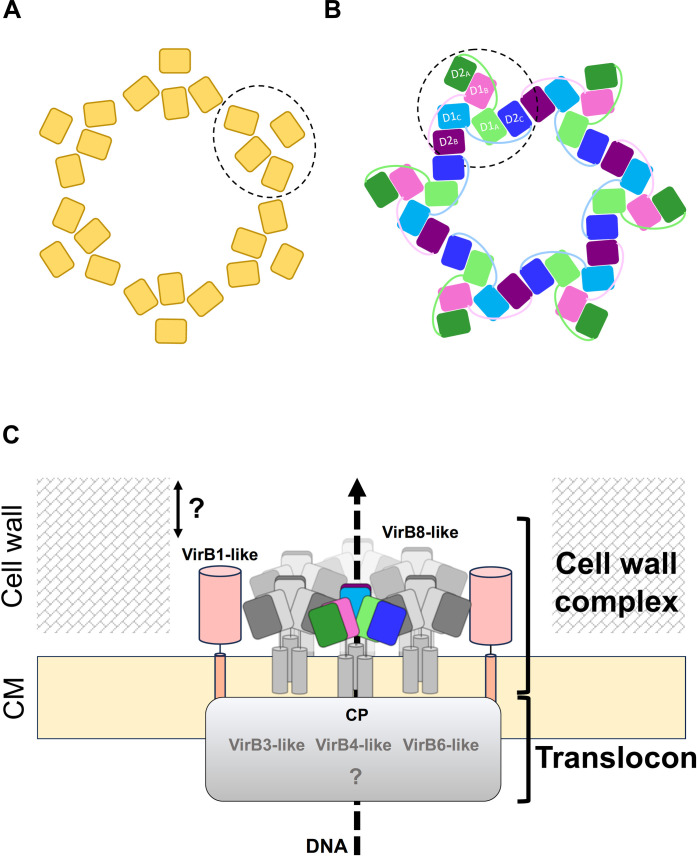
Model of conjugative T4SSs in Gram-positive bacteria. (**A**) Top view depicting the structural arrangement of NTF2-like domains of VirB8 proteins as found in the T4SS of R388 (*12*). (**B**) Proposed structural organization of NTF2-like domains of VirB8-like proteins in Gram-positive T4SSs. This assembly is likely facilitated by interactions between the peripheral D2 domains. The proposed functional units (tetramers for VirB8 or trimers for VirB8-like proteins) are surrounded by dashed lines. (**C**) Assembly model of conjugative T4SSs in Gram-positive bacteria. VirB8-like subunits form trimers anchored to the cytoplasmic membrane (CM). NTF2-like domains of VirB8-like proteins protrude in the cell wall and are surface-exposed. Trimers of VirB8-like subunits assemble via lateral interactions, forming highly ordered structures resembling those described in Gram-negative systems. VirB8-like proteins interact with VirB1-like proteins, collectively forming the cell wall complex which spans the cell wall and form the DNA translocation channel. The double arrow indicates the uncertainty (?) regarding the positioning of VirB1-like and VirB8-like components relative to the cell wall width. VirB8-like subunits contribute to the association between the cell wall complex and the translocon. The translocon comprises the CP, VirB3-like, VirB4-like, VirB6-like, and additional nonidentified subunits (?). The proposed DNA translocation pathway is indicated by a dashed arrow.

In the context of Gram-negative bacteria, the recent T4SS structure has uncovered that VirB8 subunits assemble into tetrameric subunits (*12*, *45*). These tetramers are packed within the T4SS to form a cylindrical arrangement, which creates arches surrounding the central T4SS subcomplex known as the stalk. These data indicate that despite the substantial difference in their structural arrangement (Fig. 9A), VirB8 family proteins from phylogenetically distant organisms act as functional units, trimers for VirB8-like proteins from Gram-positive bacteria, and tetramers for Gram-negative bacteria.

In the crystal structure of OrfG, the trimers display no discernible interactions between them that could suggest a higher-order assembly. Nonetheless, as indicated in a previous study (*28*) and supported by BACTH data in this study, we showed a tendency of D2 domain of OrfG to multimerize. Considering that the D2 domain occupies a peripheral position within the trimeric structure, its multimerization could potentially facilitate the assembly of a higher-ordered structure. We hypothesize that the peripheral assembly of VirB8-like trimers could form higher-ordered complexes with a cylindrical architecture in the cell wall, as observed in Gram-negative systems (Fig. 9B) (*12*).

Previously, a concept of minimal T4SS in Gram-positive bacteria was proposed (*9*). This model suggests that T4SSs form a channel across the bacterial envelope, comprising two functional modules: the translocon and the cell wall spanning complex (*9*). The translocon shares similarities with the IMC of Gram-negative bacteria regarding its composition. However, the exact composition and architecture of the cell wall complex remains unclear. This complex is anticipated to form a channel that facilitates the DNA passage across the thick cell wall. The data acquired in this study, along with previous findings gathered from other systems, allowed us to propose an assembly model of T4SSs in Gram-positive bacteria belonging to MPF_FA_ clade. This model not only experimentally supports the previously proposed concept of cell wall complex but also provides important insights (Fig. 9C).

In this model, we propose that (i) VirB8-like proteins assemble into trimers, serving as their fundamental biological unit; (ii) through interaction between there peripheral domains, VirB8-like trimers likely form cylindrical structures (Fig. 9B). These structures protrude into the cell wall and are accessible at the cell surface. The surface accessibility of VirB8-like is certainly facilitated by the action of the VirB1-like, the peptidoglycan hydrolase of the system, which is believed to create breaches in the cell wall to help the settling up of T4SS apparatus (*46*, *47*). (iii) The interaction between VirB8-like and VirB1-like subunits reported here, as well as in other systems, suggests that both proteins contribute to the assembly of the cell wall complex (*23*, *47*). (iv) The cell wall complex will serve as a conduit for the transferred DNA, likely across the cylindrical structure composed by VirB8-like subunits, and (v) VirB8-like subunits will bridge the cell wall complex to the translocon anchored in the cytoplasmic membrane through their interaction with the coupling protein, the VirB6-like, as well as the VirB4-like subunits (*23*, *48*).

Although speculative, this model is consistent with the proposed phylogenetic trajectory of conjugative T4SSs from Gram-positive bacteria, which are thought to be acquired from Gram-negative bacteria and evolved to adapt to their hosts (*20*). The more complex structures of VirB8-like proteins underscore this evolutionary adaptation, potentially to respond to the difference in structure and composition of the wall in Gram-positive versus Gram-negative bacteria. Our model also establishes a valuable framework for future research aimed at understanding the biology of conjugative T4SSs in Gram-positive bacteria. It also raises important questions: How is DNA transferred to the recipient cell, particularly in the absence of associated pili in Gram-positive systems? We also do not explain how subunits of the T4SS can extend through the cell wall and reach the cell surface. This likely involves a complex and dynamic coordination of T4SS subunits potentially requiring significant structural arrangements. Moreover, the presence of additional partners is likely necessary to establish contact with the recipient cell and to facilitate DNA translocation through conjugation tunnel. Further exploration of these aspects is needed to deepen our understanding of bacterial conjugation in Gram-positive bacteria.

## MATERIALS AND METHODS

### Bacterial strains and plasmids

Bacterial strains, plasmids, and DNA oligonucleotides are listed in tables S3 and S4.

### Culture conditions

*E. coli* strains were grown in lysogeny broth (LB) media supplemented with adequate antibiotic [ampicillin (100 μg ml^−1^), kanamycin (50 μg ml^−1^), or chloramphenicol (34 μg ml^−1^)]. *S. thermophilus* LMG18311 (WT and its derivatives; table S3) were routinely grown at 42°C in M17 broth supplemented with 0.5% lactose (LM17) and adequate antibiotic [chloramphenicol (5 μg ml^−1^) for donor strains or erythromycin (5 μg ml^−1^) for recipient strains].

### Plasmid construction

Polymerase chain reactions (PCRs) were performed using a Bio-Rad T100 thermal cycler and the Phusion High-fidelity DNA Polymerase (Thermo Fisher Scientific). *S. thermophilus* WT harboring ICE*St3* genomic DNA was used as a template for PCR amplifications. Custom oligonucleotides (Eurogentec) used for cloning procedures are listed in table S4. The procedures for constructing plasmids for protein expression were described in (*28*).

### Plasmid construction procedures

#### Knockout mutant and complementation

The 1024- to 943-bp upstream (Up) and downstream (Dn) regions of *orfG* gene on ICE*St3* genome were amplified and spliced using PCR overlap. The resulting product was inserted using classical restriction ligation procedure into pGhost9 (using Not I and Apa I enzymes) leading to pGhost9::*Up-Dn*. This construct was used to generate Δ*orfG* strain (knockout mutant of *orfG*). Protein complementation in *S. thermophilus* was performed by the insertion of *orfG* derivatives (*orfG^A204C^*, *orfG^A264C^*, and *orfG^A204C-A264C^*) at its native genetic background using the pGhost9 as a shuttle vector. First, we cloned the 1024 Up region of *orfG* in the pET28a^+^ vector using Xho I and Sac I sites. The corresponding vector was used to insert the 943 Dn region of *orfG* using Nhe I and Xba I sites leading to the pET28::*Up-Dn* construct. Next, OrfG variants coding sequences were inserted into pET28::*Up-Dn* using Sac I and Nhe I sites. Both sites were added to the native sequence by respecting the presence of *orfG* and *orfF* ribosome binding site regions. The final constructs *Up-orfG^A204C^-Dn*, *Up-orfG^A264C^-Dn*, and *Up-orfG^A204-A264C^-Dn* were subcloned into pGhost9 using Not I and Apa I enzymes.

#### Topology analysis

The pKTop vector was used to insert the encoding sequences of OrfG full-length or OrfG variants OrfG_2–37_, OrfG_2–57_, and OrfG_2–331_ in frame with the dual PhoA-LacZ reporter. The regions encoding the different OrfG variants were inserted in pKTOP using the PCR restriction-free (RF) cloning method. Briefly, the reamplified inserts which contain overlapping regions with the destination vector were used as oligonucleotides to amplify the entire destination vector leading to the insertion of desired inserts at a specific position. After DpnI treatment, DH5α cells were transformed by the PCR products, and two colonies were picked randomly for plasmid miniprep and sequencing.

#### TOXCAT plasmid construction

The pccKAN vector was provided L. Dieter. The different regions encoding for OrfG_1−63_ and OrfG_35−63_ were PCR-amplified and inserted into the pccKAN vector using Nhe I and Bam HI enzymes.

#### BACTH plasmid construction

To construct the BACTH chimeras, the sequences encoding for *OrfG* and its derivatives, as well as *OrfA*, *OrfC*, *OrfD*, *OrfE*, *OrfF*, and *OrfK*, were PCR-amplified using corresponding primers (table S2) and cloned into pKT25, pKTN25, pUT18C, and pUT18 using Pst I and Sma I enzymes.

### BACTH assay

Competent DHT1 cells were cotransformed with 30 ng each of the two recombinant plasmids encoding the fusions of T18 and T25 domains of the adenylate cyclase. From 1 ml of the transformed bacteria in LB media, 250 μl was plated on MacConkey, or LB agar plates supplemented with 5-bromo-4-chloro-3-indolyl-β-D-galactopyranoside (X-Gal) (40 μg ml^−1^), ampicillin (100 μg ml^−1^), kanamycin (50 μg ml^−1^), and 0.5 mM isopropyl-β-d-thiogalactopyranoside (IPTG) were added to MacConkey and LB agar plates. Next, plates were incubated at 30°C for 36 hours. Positive controls (Pal-18 and TolB-25) were included in each assay. From each analyzed interaction, three colonies were picked and inoculated into 600 μl of cultures in LB containing ampicillin (100 μg ml^−1^), kanamycin (50 μg ml^−1^), and 0.5 mM IPTG and grown overnight at 30°C under shaking. From each culture, 8 μl was spotted on LB agar plates supplemented with X-Gal (50 μg ml^−1^), ampicillin (100 μg ml^−1^), kanamycin (50 μg ml^−1^), and 0.5 mM IPTG. Plates were incubated at 30°C for 36 hours. β-Galactosidase activity was measured as described in (*31*).

### Topology analysis using PhoA-LacZ

*E. coli* DH5α cells expressing the different *phoA-lacZ* fusions or control vectors (pET28) were plated on LB plates supplemented with X-Pho (120 μg ml^−1^; 5-bromo-4-chloro-3-indolyl phosphate disodium salt), Salmon-Gal (100 μg ml^−1^; 6-chloro-3-indolyl-β-d-galactopyranoside), 0.5 mM IPTG, and kanamycin (50 μg ml^−1^). The plates were incubated overnight at 37°C, and the coloration was scored.

### TOXCAT assays

TOXCAT assay uses of a chimeric construct where the sequence encoding the TMD of interest is fused to *toxR*, a transcriptional regulator that acts as a dimer. The self-association of the TMD leads to ToxR dimerization which in turn activates the reporter gene encoding for the CAT and confers the resistance to chloramphenicol. The TOXCAT assays were performed according to the procedure described in (*49*) with minor modifications. *E. coli* NT326 strain was transformed with pccKAN or pccKAN harboring sequences encoding for ToxR-X-MalE fusions (X indicates OrfG N-terminal variants) and plated on M9 minimal agar medium supplemented with 0.4% glucose or 0.4% maltose as sole carbon source, ampicillin (100 μg ml^−1^), and streptomycin (50 μg ml^−1^) (fig. S1E). Plates were incubated for 48 hours at 37°C, and only colonies obtained from plates supplemented with maltose were kept for further analysis. To perform the disk diffusion assay, 36 μl of chloramphenicol (90 μg ml^−1^) was added to a nitrocellulose 6-mm filter disk (0.2-μm pore size from Millipore) disposed on a sterile empty petri dish. After drying, disks were deposited at the center of the LB agar plate supplemented with ampicillin (100 μg ml^−1^) and incubated for 3 hours at 37°C before removing the disk. Next, 2 ml of *E. coli* NT326 cultures producing TOXCAT fusions was spread, and plates were incubated ON at 37°C. Diameters of halos resulting from the resistance to chloramphenicol were measured and recorded. The recorded values represent the results of a single experiment from a set of triplicates, each time conducted with three distinct colonies. For CAT activity measurement, from 10 ml of cultures of NT326 strain producing TOXCAT fusions, 3 ml at OD_600_ (optical density at 600 nm) = 0.5 was pelleted and conserved at −20°C. Following a 20-min thawing period at room temperature, the pellet was collected using 0.5 ml of lysis buffer [25 mM tris-HCl and 2 mM EDTA (pH 8.0)] and subsequently disrupted through sonication. The supernatant was then collected by centrifugation for 10 min at 12,000*g*. Fifteen microliters of the supernatant was mixed with 220 μl of reaction buffer [0.1 mM acetyl-CoA, 5,5′-dithiobis-(2-nitrobenzoic acid) (0.4 mg ml^−1^), and 100 mM tris-HCl (pH 7.8)] on a 96-well microplate, and the absorbance was acquired at λ = 412 and 550 nm at 10-s intervals for 4 min to measure the background rate of the acetyl-CoA hydrolysis. Then, 15 μl of 2.5 mM chloramphenicol was added to each reaction, and the absorbance was recorded for 10 min as described previously. The slope of each recorded assay was converted to CAT activity units.

### Site-directed mutagenesis

Cysteine point mutations were inserted into OrfG_Ext_ by RF cloning using two complementary oligonucleotides harboring the desired mutation. In cases where we failed to insert mutations using RF cloning, we used an alternative strategy: The regions surrounding the mutation are amplified with F1/R1 and F2/R2 oligonucleotides. The R1/F2 oligos have an overlapping region that also contains the mutation. The PCR products are spliced using the F1/R2 oligos, and the resulting product is inserted into the pET28 vector by a restriction-ligation procedure as detailed in (*28*).

### Protein production and purification

Protocols used for soluble protein expression and purification are described in (*28*). For membrane protein expression, we followed the same protocol used for soluble proteins. For purification, the cell pellets obtained from 1 liter of cell culture were resuspended in 40 ml of buffer A [50 mM tris-HCl (pH 8), 300 mM NaCl, and 10 mM imidazole]. Resuspended cells were homogenized and supplemented with deoxyribonuclease I (DNase I; 20 μg ml^−1^), lysozyme (50 μg ml^−1^), and 20 mM MgSO_4_. The cell suspension was mixed using stirring bar for 20 min at cold room. Afterward, the resuspension was sonicated for 5 cycles at 50%. Following cell lysis, insoluble material was pelleted by centrifugation at 20,000*g* for 40 min at 4°C in a JA 25.50 rotor. The membrane fraction was then separated from the supernatant by ultracentrifugation using a Ti90 rotor at 200,000*g* for 100 min at 8°C. The pellet was then incubated with buffer A supplemented by 1% octyl glucose neopentyl glycol (OGNG). After 1-hour incubation, the solubilized proteins were isolated by a second ultracentrifugation at 200,000*g* for 100 min at 8°C. The supernatant was then loaded onto a packed 5-ml nickel-nitrilotriacetic acid (Ni-NTA) column (Cytiva) mounted on AKTA pure (Cytiva) and equilibrated in buffer B (buffer A supplemented with 0.1% OGNG). Bound proteins were eluted using buffer C (buffer B supplemented with 250 mM imidazole). After verifying sample purity by SDS-PAGE, fractions containing the target proteins were pooled and further purified by SEC on a Superose 6 10/300 Increase column equilibrated in 50 mM tris-HCl (pH 8), 300 mM NaCl, and 0.1% OGNG. Peak fractions were pooled and stored at −80°C until use.

### SEC-MALS analysis

SEC-MALS analyses were conducted using MiniDAWN TREOS II (Wyatt) as the MALS detector and Optilab T-rEX (Wyatt) as the refractive index detector. Both were mounted on a fast protein liquid chromatography (FPLC) system (AKTA purifier, Cytiva). For soluble proteins, SEC-MALS analyses were performed using Superdex 200 10/100 Increase column (Cytiva) equilibrated on 50 mM tris-HCl and 100 mM NaCl (pH 8). For membrane proteins, analyses were carried on using the Superose 6 10/300 Increase column (Cytiva) using the 50 mM tris-HCl (pH 8), 300 mM NaCl, and 0.1% OGNG.

### Cysteine cross-linking analysis in vitro

After the Tobacco etch virus protease (TEV) cleavage step performed on purified thioredoxin-fused constructs (OrfG_Ext_, Orf13^WT^, or their cysteines variants), the untagged proteins were recovered after a reverse immobilized metal affinity chromatography (IMAC) purification. Afterward, 12 μl of untagged proteins was mixed with 4 μl of Laemmli loading buffer [125 mM tris-HCl (pH 6.8), 0.002% bromophenol blue, 2% SDS, and 10% glycerol] with or without 20 mM DTT. The sample was next heated at 95°C for 10 min before their analysis by 12% SDS-PAGE.

### OPA and OPIA

The OPA and OPIA experiments were performed as previously described (*30*). Briefly, the WT strain, complement, white blood cells, and rabbit sera were prepared individually before the assay as follows. The bacterial culture was diluted in RPMIF (RPMI 1640 medium supplemented with 15% fetal bovine serum) to yield a final concentration of 2 × 10^6^ cells ml^−1^. Lyophilized baby rabbit complement (Cedarlane) was dissolved in RPMIF at 6.7%, absorbed for 60 min at 4°C with the target bacterial strain, and sterilized by filtration before use. Rabbit sera or purified antibodies were diluted in RPMIF at the desired concentrations. White blood cells were freshly prepared from human blood specimens and adjusted to a final concentration of 2 × 10^6^ cells ml^−1^. To overcome the nonspecific binding of preimmunized rabbit sera to *S. thermophilus*, rabbit sera were incubated with an excess of Δ*orfG* strain freshly grown overnight at 37°C in brain heart infusion agar (Carl Roth) and incubated for 60 min with gentle shaking. Then, the mixture was centrifuged and filtered sterile. Last, the supernatant was used in the OPA as described above.

For the OPIA, purified antibodies were mixed with the recombinant OrfG_Ext_ protein. Concentrations ranging from 1.6 to 26.6 μM purified OrfG_Ext_ were incubated overnight at 4°C with an equal volume of the purified antibody at the indicated concentration. After incubation, the mixture of OrfG_Ext_/sera was used as a source of antibodies in the OPA as described above.

The percentage of killing was determined by comparing the surviving colony-forming units in the reaction in the presence of neutrophils to the reaction conducted without neutrophils. Negative controls lacking one, two, or three of the components were included in the assay.

### Whole bacterial cellular ELISA

Immunoreactivity against the WT and Δ*orfG* strains was measured by whole-cell ELISAs using anti-OrfG purified antibodies (Ab-G) following the previously described protocol (*30*). Bacteria were grown on tryptic soy agar plates and incubated overnight at 37°C. Colonies were collected from the plate and inoculated in 50 ml of brain heart infusion broth (Carl Roth) at an OD_650_ = 0.1. Cultures were grown to an OD_650_ = 0.4 and harvested by centrifugation at 7450*g* and 4°C for 10 min. Cell pellets were washed twice with 50 ml of phosphate-buffered saline (PBS), resuspended in 25 ml of 8% PFA (Sigma-Aldrich) in PBS, and incubated at 4°C for 30 min under gentle shaking. Then, cells were washed twice with 25 ml of PBS and lastly resuspended in 10 ml of 0.2 M sodium carbonate/bicarbonate buffer. Nunc-immuno Maxisorp 96-well plates were coated with cell suspension (100 μl per well) and incubated overnight at 4°C. Next, wells were washed three times with 200 μl of PBST (phosphate-buffered saline with 0.05% Tween 20 at pH 7.4). Wells were blocked with 200 μl of blocking buffer (3% BSA in PBS) at room temperature for 2 hours and washed three times with 200 μl of PBST. Sera were adjusted to immunoglobulin G (IgG; 100 μg ml^−1^) in blocking buffer, and twofold serial dilutions were made in the same buffer. After that, 100 μl of serum dilutions were added in triplicate, and plates were incubated for 1 hour. Wells were washed three times with 200 μl of PBST. Later, 100 μl of alkaline phosphatase–conjugated polyclonal antirabbit IgG produced in goat (Sigma-Aldrich) was added to each well at 1:1000, and plates were incubated for 1 hour. Last, wells were washed four times with 200 μl of PBS, and 100 μl of the p-nitrophenyl phosphate substrate (Sigma-Aldrich) at 1 mg ml^−1^ in glycine buffer was added to each well before incubation for 30 min. To stop the reaction, 3 M NaOH (50 μl per well) was added, and the absorbance was measured at 405 nm in an ELISA reader (Synergy H1 Hybrid reader, BioTek). The immunoreactivity with an Ab-G was calculated as the difference of absorbance of the well with Ab-G to the absorbance of the blank.

### Crystallization and structure determination

OrfG_Ext_ and its variant OrfG_Ext_-A204C-A264C were crystallized using the same sitting drop protocol: Drops were prepared with an Oryx 8 robot (Douglas Instruments) by mixing 0.3 μl of the protein solution and 0.3 μl of the crystallization solution, which were deposited in the wells of a 96-well plate. In addition, 50 μl of the crystallization solution was deposited in the corresponding reservoirs. Good crystals were obtained at 20°C when the protein solution contained either OrfG_Ext_ (31 mg ml^−1^) in 100 mM tris-HCl (pH 8.0) or OrfG_Ext_-A204C-A264C (33 mg ml^−1^) in 50 mM tris-HCl (pH 8.0) and 100 mM NaCl. The associated crystallization solution contained 1.26 M ammonium sulfate and 100 mM sodium cacodylate (pH 6.5) (condition Wizard classic 1-2 B1, Rigaku) for the WT protein or 40% (v/v) polyethylene glycol 300 and 100 mM potassium phosphate citrate (pH 4.2) (condition JCSG++ C6, Jena Biosciences) for the mutant. Crystals were fished with a Lytholoop (Molecular Dimension) and flash-frozen in a nitrogen flux at 100 K. No cryoprotection was necessary for the mutant, while a quick immersion in the crystallization solution added of 20% glycerol was necessary for the WT.

Diffraction data were collected on the synchrotron beamline Proxima 2-A at SOLEIL (France) for OrfG_Ext_ and on beamline MASSIF-3/ID30A-3 at ESRF (France) for OrfG_Ext_-A204C-A264C. Images were processed using x-ray detector software (XDS), and datasets were scaled using Aimless Collaborative Computational Project no. 4 (CCP4) (*50*), with a maximum resolution of 1.84 Å for the WT and 2.3 Å for the mutant (see table S1). The structure of OrfG_Ext_ was solved by molecular replacement with MOLREP (*51*), using a two-part phasing model composed of the domain D1 taken from our previous study of this sole domain [PDB code 6zgn; (*28*)] and domain D2 taken from the structure of TcpC [PDB entry 3ub1; (*23*)]. The structure of OrfG_Ext_-A204C-A264C was also solved by molecular replacement, using the structure of the WT. However, this step failed when using the entire structure as the search model, requiring the separation of domains D1 and D2 for their correct positioning and successful phasing. Both structures were rebuilt using Coot (*52*). The WT structure was refined using PHENIX (*53*) while that of the mutant was refined using Refmac5 (*54*) (see table S1).

The structure of OrfG_Ext_ (available at the PDB under the accession code 7pkw) contains one monomer (residues 86 to 329) per asymmetric unit, and the trimer is generated by the threefold axis of the cubic crystals. N-terminal residues 64 to 85, as well as 170 to 173 and 330 to 331 (C terminus), were not observed in the electron density. The structure of the variant OrfG_Ext_-A204C-A264C contains a trimer in the asymmetric unit. In this case, residues 89 to 331 were observed in the electron density, while the structure still misses residues 171 to 172 in chain B. The electron density unambiguously shows the three expected disulfide bridges, observed between Cys^204^ of monomer A and Cys^264^ of monomer C (named Cys204A-Cys264C), Cys204B-Cys264A, and Cys204C-Cys264B. This structure can be retrieved from the PDB with the accession code 8s7l.

### Sequence alignment, structure comparison, and interface analysis

The structures of OrfG_Ext_ (PDB entry 7pkw), its double mutant OrfG_Ext_-A204C-A264C (8s7l), and TcpC (3ub1) were compared after their superimposition performed by mTM-align (*55*) which does not consider the nature of amino acids but their position. The sequence alignment based on the structural superposition of the models was then slightly modified by hand in the regions where mTM-align did not find a concordance (lowercase letters in Fig. 4E), based on sequence alignment. Measurements aimed at comparing the geometry of the different trimers were performed as follows. All possible trimers were considered, either directly observed in the asymmetric unit (one trimer for the mutant A204C-A264C and two trimers for TcpC) or generated by a crystallographic threefold axis (OrfG). From each trimer, the coordinates of the three domains D1 (residues 92 to 204 in OrfG, 104 to 229 in TcpC) and the three domains D2 (228 to 320 in OrfG, 248 to 346 in TcpC) were extracted to get six subunits. Arithmetical means of coordinates were calculated for each of them to determine their center, and then all possible distances were measured between these points and summarized in Fig. 5D (means and RMSD of equivalent distances). To measure the structural difference between two models (or subparts of models), the TM score determined for structures optimally superposed by mTM-align was used. Different models were formed: domains D1 or D2 alone, pairs (D1:D1′) or trimers (D1:D1′:D1″) of D1, concatenations of D1 and D2 from one single monomer (D1:D2), or concatenations of domains D1 and D2 of two adjacent monomers (D1:D2′), as observed in the structures. The values are summarized in table S2 (average TM scores calculated for all possible superpositions of equivalent models in all observed trimers). The lists of the residues that were involved in interactions between monomers in the different trimers were obtained from PISA (*35*) and then reduced on the basis of a distance criterion (4 Å).

### Phylogenetic analysis

Protein sequences were retrieved from the National Center for Biotechnology Information and aligned using the T coffee expresso multiple alignment program (*56*) available online (https://tcoffee.crg.eu/). Phylogenetic trees were constructed with MEGA11 (*57*) using the maximum likelihood model.

### Knockout mutant construction

#### S. thermophilus transformation

WT or its derivatives were grown overnight in LM17 at 42°C. Twenty microliters of these cultures was mixed with 300 ng of pGhost9 or its derivatives and 3 μl of LPYFAGCL synthetic peptide (250 nM) to induce competence. Minimal chemically defined medium was added to reach 200-μl final volume. After incubation at 42°C for 4 to 5 hours, the mixture was plated on suitable agar plates with the adequate antibiotic and incubated overnight at 42°C.

#### Plasmid curing

Strains transformed with pGhost9 or its derivatives were plated on agar plates with erythromycin (5 μg ml^−1^) and incubated overnight at 42°C. The integration of pGhost9 on the obtained clones was confirmed by screen PCR. Four colonies were selected for plasmid curing. First, these colonies were used to inoculate 5 ml of LM17 in the presence of erythromycin (5 μg ml^−1^) and incubated overnight at 42°C. Then, 100 μl from each preculture was mixed with 900 μl of LM17 in the presence of chloramphenicol (5 μg ml^−1^), and the mixture which constituted the 10^−1^ dilution was used to make serial dilutions from 10^−2^ to 10^−6^. All dilutions were incubated at 30°C for 7 to 8 hours to allow plasmid replication. New serial dilutions were started from 10^−3^ or 10^−4^ cultures and incubated at 30°C overnight. Additional serial dilutions were made to allow plasmid curing for at least six generations. From the last freshly prepared serial dilution, 50, 75, and 100 μl from 10^−4^, 10^−5^, and 10^−6^ dilutions, respectively, were spread on LM17 agar plates supplemented with chloramphenicol (5 μg ml^−1^) and incubated overnight at 42°C. One hundred clones from the corresponding plates were spotted on separate LM17 agar plates supplemented with erythromycin (5 μg ml^−1^) or chloramphenicol (5 μg ml^−1^). Colonies that are erythromycin sensitive and chloramphenicol resistant were subjected to PCR screening to identify the mutant strains among those who recovered the WT genotype.

### Immunolocalization of OrfG and its cysteine variants

Subcellular fractionation of *S. thermophilus*, ∆*orfG* strain or ∆*orfG* strain producing OrfG cysteine variants, was performed according to protocol described in (*24*) with some modifications. Five milliliters from 20 ml of overnight cultures was used to inoculate 500 ml of culture cells (1:100 dilution). Cells were grown at 42°C until OD_600_ = 1.8. Cells were centrifuged at 5000*g*, and the corresponding cell pellets were frozen at −80°C. Before lysis, cells were resuspended in 10 ml of lysis buffer [1× PBS buffer, 20 mM MgSO_4_, 2 mM EDTA, lysozyme (50 μg ml^−1^), 20 μg of DNase I, and complete EDTA free (Roche)] and incubated 30 min with agitation at 4°C. Cell lysis was supplemented by a sonication step (3 to 5 cycles of 1-min sonication at 50% power). Unlysed cells were removed by low-speed centrifugation. The soluble and membrane fractions were separated by ultracentrifugation of the supernatant at 50,000 rpm for 1 hour at 4°C using the Ti 70 rotor on ultracentrifuge (OPTIMA XE 90, Beckman). Before Western blot analysis of the different fractions, 20 μl of mutanolysin (50 mg ml^−1^) was added to 100 μl of membrane fraction and incubated for 10 min at 37°C. The immunodetection of OrfG on the total, soluble, and membrane fractions was conducted using purified Ab-G antibodies (Biotem) (1:2000 dilution) and secondary horseradish peroxidase–conjugated antirabbit antibodies (1:5000 dilution) (Bio-Rad).

To analyze the cysteine cross-linked complexes, 15 μl of membrane fractions was mixed with either 2 μl of DTT (20 mM) or 2 μl of 50 mM tris-HCl buffer and incubated for 10 min at room temperature. Subsequently, 5 μl of Laemmli blue loading buffer was added to each sample and heated for 10 min at 95°C. The DTT-treated and DTT-untreated samples were loaded onto 10% SDS-PAGE and blotted onto nitrocellulose membranes before their Western blot analysis using Ab-G antibodies as described above.

### Biparental mating

To allow a tight contact between donor and recipient cells during conjugation, the mixture of donor and recipient cells was encapsulated in alginate beads as described below. The strategy for bacterial encapsulation in alginate beads was adapted from (*58*), with slight modifications. The following solutions for encapsulation were prepared in demineralized water and sterilized at 120°C for 15 min: 6% sodium alginate, 0.4 M calcium chloride dihydrate, physiological saline solution, and EDTA citrate solution (50 mM EDTA, 55 mM sodium citrate, and 150 mM sodium chloride). One hundred fifty microliters from 5 ml of donor and recipient cell precultures was used to inoculate 15 ml of LM17 broth supplemented with appropriate antibiotic. Cultures were incubated at 42°C to reach OD_600_ of 0.4. A total of 12.5 ml of each culture was mixed to obtain cell suspension at OD_600_ of 5. Next, cells were centrifuged at 2694*g* for 10 min at 20°C. The pellet was then resuspended in 1 ml of physiological saline solution. One hundred fifty microliters from bacterial suspension was mixed with of 850 μl of alginate solution. The mixture was collected in a 1-ml pipette tip using a 1000-μl pipette (Eppendorf) and then manually dropped into 13 ml of calcium chloride solution with a constant flow to ensure uniform volumes of alginate beads. Beads were incubated for 10 min at room temperature and rinsed two times with physiological saline solution. The obtained alginate beads were incubated in LM17 (15 ml) for 4 hours at 42°C. Enumeration of entrapped bacteria (donors and recipients) was realized before each in vitro experiment: Beads were chemically disrupted in EDTA citrate solution (15- to 20-min incubation with soft shaking) to liberate bacteria. The mixture was then centrifuged (10 min at 3200*g*) at room temperature, and the pellet was resuspended in 1 ml of physiological saline solution. Next, 10-fold serial dilutions were prepared in physiological saline solution, ranging from 10^−1^ to 10^−6^. The transconjugants were recovered by plating 100 μl from 10^−1^ and 10^−2^ dilutions on an LM17 agar plate supplemented with erythromycin (5 μg ml^−1^) and chloramphenicol (5 μg ml^−1^) and incubated for 24 hours at 42°C. In this experiment, the donor cells are chloramphenicol resistant, whereas the recipient cells are erythromycin resistant.

### Cysteine cross-linking analysis

After the TEV cleavage step had been performed on purified thioredoxin-fused constructs (OrfG_Ext_ or Orf13^WT^ and their cysteine variants, OrfG-A204C, OrfG-A264C, OrfG-A204-A264C, Orf13-V164C, Orf13-Q280C, and Orf13-V164C-Q280C), the untagged proteins were recovered after a reverse IMAC purification. Next, 45 μl of untagged proteins was mixed with 5 μl of 10% SDS. The mixture was heated at 95°C for 10 min. Afterward, 5 μl of DTT (20 mM) was added, and the reaction mixture was incubated at room temperature for 10 min. Last, 12 μl of Laemmli loading buffer [125 mM tris-HCl (pH 6.8), 0.002% bromophenol blue, 2% SDS, and 10% glycerol] was added, and the samples were analyzed by 12% SDS-PAGE. To examine the cysteine cross-linked complexes in the membrane fractions, 75 μl of membrane fractions was mixed with either 10 μl of DTT (20 mM) or 10 μl of 50 mM tris-HCl buffer and incubated for 10 min at room temperature. Subsequently, 25 μl of Laemmli blue loading buffer was added to each sample and heated for 10 min at 95°C. Next, 10 μl of DTT-treated and DTT-untreated samples were loaded onto 10% SDS-PAGE and blotted onto nitrocellulose membranes before their Western blot analysis using Ab-G antibodies as described above.

### Real-time switchSENSE analysis

The switchSENSE DRX technology (Dynamic Biosensors GmbH, Martinsried, Germany) was used to characterize the DNA-protein interaction. The principle of the technology is explained in details in (*39*). To characterize OrfG-DNA interaction through molecular dynamic, switchSENSE experiments were carried out with designed DNA sequences called Overhang (ssOV and ssOV-NS). These DNA sequences are composed both by a DNA sequence originated from ICE*St3* (for ssOV) or a nonspecific DNA sequence (for ssOV-NS) and by a region of single-stranded complementary sequence of the Nanolever (c-NV) fixed to the biochip (fig. S9A). In this experiment, ssDNA (ssOV or ssOV-NS) and dsDNA (ssOV + C-DNA or ssOV-NS + c-DNA-NS) were used. To hybridize the two strands (listed in table S4), 1 μM of each sequence was incubated for 30 min at room temperature in PE40 buffer (pH 7.4). Measurements were carried out in the dynamic mode, where DNA Nanolevers are electrically driven to oscillate at high frequency. An analyte-ligand interaction leads to a slowing down of the oscillation, decreasing the dynamic response and leading to a modification of fluorescence which can be used to determine ligand sizing index. All assays were performed with different OrfG_Ext_ concentrations [30, 60, and 120 μM dilution performed in 20 mM Ttis-HCl (pH 8.0) and 100 mM NaCl] and 200 nM ssDNA or dsDNA at 25°. OrfG_Ext_ sizing index was calculated by comparing the switching dynamics of bound protein with those of bare DNA and with the lollipop biophysical model (*39*). After each assay, a regeneration solution (alkaline solution pH 13) was injected to remove the ligand and initiate new measurement. Result analysis was processed with the switchANALYSIS software from Dynamic Biosensors.

### In vitro chemical cross-linking

Cross-linking experiments between ssDNA and OrfG_Ext_ or BSA were carried out in 20 mM Hepes (pH 8.0) and 100 mM NaCl buffer. The ICE*St3* DNA sequence used is labeled at 5′ end with 6-FAM (6-carboxyfluorescein) called hereafter ssDNA*. The used protocol is described as follows: ssDNA* (0.2 μM) was mixed with increasing quantities of proteins OrfG_Ext_ or BSA from 0 to 0.6 μg and incubated for 15 min at 37°C. To stabilize any formed complex, PFA at 0.5% final concentration was then added to the samples and incubated for 1 hour with the reaction mixture at 37°C. The cross-linking reaction was stopped by adding tris-HCl at a final concentration of 80 mM, and the mixture was incubated for 10 min at room temperature. To examine cross-linked products, 2 μl of 4× DNA loading buffer (25 mg of bromophenol blue, 4 g of sucrose, and the volume was adjusted to 10 ml by adding H_2_O) was added and 18 μl from each reaction was analyzed by 8 and 3% mix native PAGE. The mixed 8 and 3% native PAGE also contained glycerol 2.5% and 0.5× TBE (tris-HCl, borate, EDTA). The 8 to 3% acrylamide gels were cast in two stages: the lower half with 8% acrylamide solution and the upper half with 3% acrylamide solution. The 8% section allowed the visualization of small DNA fragments, whereas the 3% facilitated the detection of larger DNA-protein complexes which have difficulty in penetrating the classical 5% acrylamide gels. The samples were migrated for 1.5 hours at 84 V in TBE buffer (0.5×). Cross-linked products were visualized using ChemiDoc (Bio-Rad).

### Statistical analysis

The statistical significance was performed by the *t* test using GraphPad Prism (nonparametric test, followed by the impaired test). Bars and whiskers denote mean values and SEM; not significant (ns) (*P* ≥ 0.05), *P < 0.05, **P < 0.01, ***P < 0.001, and ****P < 0.0001.

## Supplementary Materials

### This PDF file includes:

Tables S1 to S4 Figs. S1 to S10 References

## References

[1] S. M. Soucy, J. Huang, J. P. Gogarten, Horizontal gene transfer: Building the web of life. Nat. Rev. Genet. 16, 472–482 (2015). 26184597 10.1038/nrg3962

[2] E. Grohmann, P. J. Christie, G. Waksman, S. Backert, Type IV secretion in Gram-negative and Gram-positive bacteria. Mol. Microbiol. 107, 455–471 (2018). 29235173 10.1111/mmi.13896 PMC5796862

[3] X. Bellanger, S. Payot, N. Leblond-Bourget, G. Guedon, Conjugative and mobilizable genomic islands in bacteria: Evolution and diversity. FEMS Microbiol. Rev. 38, 720–760 (2014). 24372381 10.1111/1574-6976.12058

[4] S. R. Partridge, S. M. Kwong, N. Firth, S. O. Jensen, Mobile genetic elements associated with antimicrobial resistance. Clin. Microbiol. Rev. 31, e00088-17 (2018). 30068738 10.1128/CMR.00088-17 PMC6148190

[5] T. R. D. Costa, J. B. Patkowski, K. Macé, P. J. Christie, G. Waksman, Structural and functional diversity of type IV secretion systems. Nat. Rev. Microbiol. 22, 170–185 (2024). 37814112 10.1038/s41579-023-00974-3 PMC11290344

[6] E. Cascales, P. J. Christie, The versatile bacterial type IV secretion systems. Nat. Rev. Microbiol. 1, 137–149 (2003). 15035043 10.1038/nrmicro753 PMC3873781

[7] C. E. Alvarez-Martinez, P. J. Christie, Biological diversity of prokaryotic type IV secretion systems. Microbiol. Mol. Biol. Rev. 73, 775–808 (2009). 19946141 10.1128/MMBR.00023-09 PMC2786583

[8] R. Fronzes, P. J. Christie, G. Waksman, The structural biology of type IV secretion systems. Nat. Rev. Microbiol. 7, 703–714 (2009). 19756009 10.1038/nrmicro2218 PMC3869563

[9] M. Bhatty, J. A. Laverde Gomez, P. J. Christie, The expanding bacterial type IV secretion lexicon. Res. Microbiol. 164, 620–639 (2013). 23542405 10.1016/j.resmic.2013.03.012 PMC3816095

[10] P. J. Christie, The mosaic type IV secretion systems. EcoSal Plus 7, 10.1128/ecosalplus.esp-0020-2015 (2016). 10.1128/ecosalplus.esp-0020-2015 PMC5119655 27735785

[11] T. R. D. Costa, L. Harb, P. Khara, L. Zeng, B. Hu, P. J. Christie, Type IV secretion systems: Advances in structure, function, and activation. Mol. Microbiol. 115, 436–452 (2021). 33326642 10.1111/mmi.14670 PMC8026593

[12] K. Mace, A. K. Vadakkepat, A. Redzej, N. Lukoyanova, C. Oomen, N. Braun, M. Ukleja, F. Lu, T. R. D. Costa, E. V. Orlova, D. Baker, Q. Cong, G. Waksman, Cryo-EM structure of a type IV secretion system. Nature 607, 191–196 (2022). 35732732 10.1038/s41586-022-04859-y PMC9259494

[13] M. J. Sheedlo, M. D. Ohi, D. B. Lacy, T. L. Cover, Molecular architecture of bacterial type IV secretion systems. PLOS Pathog. 18, e1010720 (2022). 35951533 10.1371/journal.ppat.1010720 PMC9371333

[14] D. Chetrit, B. Hu, P. J. Christie, C. R. Roy, J. Liu, A unique cytoplasmic ATPase complex defines the *Legionella pneumophila* type IV secretion channel. Nat. Microbiol. 3, 678–686 (2018). 29784975 10.1038/s41564-018-0165-z PMC5970066

[15] B. Hu, P. Khara, L. Song, A. S. Lin, A. E. Frick-Cheng, M. L. Harvey, T. L. Cover, P. J. Christie, In situ molecular architecture of the *Helicobacter pylori* Cag type IV secretion system. mBio 10, 10.1128/mbio.00849-19 (2019). 10.1128/mBio.00849-19 PMC6520456 31088930

[16] P. Khara, L. Song, P. J. Christie, B. Hu, In situ visualization of the pKM101-encoded type IV secretion system reveals a highly symmetric ATPase energy center. mBio 12, e0246521 (2021). 34634937 10.1128/mBio.02465-21 PMC8510550

[17] X. Liu, P. Khara, M. L. Baker, P. J. Christie, B. Hu, Structure of a type IV secretion system core complex encoded by multi-drug resistance F plasmids. Nat. Commun. 13, 379 (2022). 35046412 10.1038/s41467-022-28058-5 PMC8770708

[18] D. Ghosal, K. C. Jeong, Y. W. Chang, J. Gyore, L. Teng, A. Gardner, J. P. Vogel, G. J. Jensen, Molecular architecture, polar targeting and biogenesis of the *Legionella* Dot/Icm T4SS. Nat. Microbiol. 4, 1173–1182 (2019). 31011165 10.1038/s41564-019-0427-4 PMC6588468

[19] J. Guglielmini, L. Quintais, M. P. Garcillan-Barcia, F. de la Cruz, E. P. Rocha, The repertoire of ICE in prokaryotes underscores the unity, diversity, and ubiquity of conjugation. PLOS Genet. 7, e1002222 (2011). 21876676 10.1371/journal.pgen.1002222 PMC3158045

[20] J. Guglielmini, F. de la Cruz, E. P. Rocha, Evolution of conjugation and type IV secretion systems. Mol. Biol. Evol. 30, 315–331 (2013). 22977114 10.1093/molbev/mss221 PMC3548315

[21] N. Goessweiner-Mohr, K. Arends, W. Keller, E. Grohmann, Conjugative type IV secretion systems in Gram-positive bacteria. Plasmid 70, 289–302 (2013). 24129002 10.1016/j.plasmid.2013.09.005 PMC3913187

[22] F. Jager, A. Lamy, W. S. Sun, N. Guerini, R. P. Berntsson, Structure of the enterococcal T4SS protein PrgL reveals unique dimerization interface in the VirB8 protein family. Structure 30, 876–885.e5 (2022). 35429437 10.1016/j.str.2022.03.013

[23] C. J. Porter, R. Bantwal, T. L. Bannam, C. J. Rosado, M. C. Pearce, V. Adams, D. Lyras, J. C. Whisstock, J. I. Rood, The conjugation protein TcpC from *Clostridium perfringens* is structurally related to the type IV secretion system protein VirB8 from Gram-negative bacteria. Mol. Microbiol. 83, 275–288 (2012). 22150951 10.1111/j.1365-2958.2011.07930.x

[24] N. Goessweiner-Mohr, L. Grumet, K. Arends, T. Pavkov-Keller, C. C. Gruber, K. Gruber, R. Birner-Gruenberger, A. Kropec-Huebner, J. Huebner, E. Grohmann, W. Keller, The 2.5 Å structure of the *Enterococcus* conjugation protein TraM resembles VirB8 type IV secretion proteins. J. Biol. Chem. 288, 2018–2028 (2013). 23188825 10.1074/jbc.M112.428847 PMC3548508

[25] C. Fercher, I. Probst, V. Kohler, N. Goessweiner-Mohr, K. Arends, E. Grohmann, K. Zangger, N. H. Meyer, W. Keller, VirB8-like protein TraH is crucial for DNA transfer in Enterococcus faecalis. Sci. Rep. 6, 24643 (2016). 27103580 10.1038/srep24643 PMC4840375

[26] C. Baron, VirB8: A conserved type IV secretion system assembly factor and drug target. Biochem. Cell Biol. 84, 890–899 (2006). 17215876 10.1139/o06-148

[27] L. Dobson, I. Reményi, G. E. Tusnády, CCTOP: A consensus constrained TOPology prediction web server. Nucleic Acids Res. 43, W408–W412 (2015). 25943549 10.1093/nar/gkv451 PMC4489262

[28] J. Cappele, A. Mohamad Ali, N. Leblond-Bourget, S. Mathiot, T. Dhalleine, S. Payot, M. Savko, C. Didierjean, F. Favier, B. Douzi, Structural and biochemical analysis of OrfG: The VirB8-like component of the conjugative type IV secretion system of ICE *St3* from *Streptococcus thermophilus*. Front. Mol. Biosci. 8, 642606 (2021). 33816557 10.3389/fmolb.2021.642606 PMC8012802

[29] G. Karimova, D. Ladant, Defining membrane protein topology using pho-lac reporter fusions. Methods Mol. Biol. 1615, 129–142 (2017). 28667608 10.1007/978-1-4939-7033-9_10

[30] F. Romero-Saavedra, D. Laverde, E. Kalfopoulou, C. Martini, R. Torelli, D. Martinez-Matamoros, M. Sanguinetti, J. Huebner, Conjugation of different immunogenic enterococcal vaccine target antigens leads to extended strain coverage. J. Infect. Dis. 220, 1589–1598 (2019). 31289829 10.1093/infdis/jiz357 PMC6782101

[31] A. Battesti, E. Bouveret, The bacterial two-hybrid system based on adenylate cyclase reconstitution in *Escherichia coli*. Methods 58, 325–334 (2012). 22841567 10.1016/j.ymeth.2012.07.018

[32] W. P. Russ, D. M. Engelman, TOXCAT: A measure of transmembrane helix association in a biological membrane. Proc. Natl. Acad. Sci. U.S.A. 96, 863–868 (1999). 9927659 10.1073/pnas.96.3.863 PMC15316

[33] L. Holm, C. Sander, Dali: A network tool for protein structure comparison. Trends Biochem. Sci. 20, 478–480 (1995). 8578593 10.1016/s0968-0004(00)89105-7

[34] Y. Zhang, J. Skolnick, TM-align: A protein structure alignment algorithm based on the TM-score. Nucleic Acids Res. 33, 2302–2309 (2005). 15849316 10.1093/nar/gki524 PMC1084323

[35] E. Krissinel, K. Henrick, Inference of macromolecular assemblies from crystalline state. J. Mol. Biol. 372, 774–797 (2007). 17681537 10.1016/j.jmb.2007.05.022

[36] E. Garriga, P. Di Tommaso, C. Magis, I. Erb, L. Mansouri, A. Baltzis, E. Floden, C. Notredame, Multiple sequence alignment computation using the T-coffee regressive algorithm implementation. Methods Mol. Biol. 2231, 89–97 (2021). 33289888 10.1007/978-1-0716-1036-7_6

[37] J. Jumper, R. Evans, A. Pritzel, T. Green, M. Figurnov, O. Ronneberger, K. Tunyasuvunakool, R. Bates, A. Zidek, A. Potapenko, A. Bridgland, C. Meyer, S. A. A. Kohl, A. J. Ballard, A. Cowie, B. Romera-Paredes, S. Nikolov, R. Jain, J. Adler, T. Back, S. Petersen, D. Reiman, E. Clancy, M. Zielinski, M. Steinegger, M. Pacholska, T. Berghammer, S. Bodenstein, D. Silver, O. Vinyals, A. W. Senior, K. Kavukcuoglu, P. Kohli, D. Hassabis, Highly accurate protein structure prediction with AlphaFold. Nature 596, 583–589 (2021). 34265844 10.1038/s41586-021-03819-2 PMC8371605

[38] J. M. Auchtung, N. Aleksanyan, A. Bulku, M. B. Berkmen, Biology of ICE*Bs1*, an integrative and conjugative element in *Bacillus subtilis*. Plasmid 86, 14–25 (2016). 27381852 10.1016/j.plasmid.2016.07.001

[39] A. Langer, P. A. Hampel, W. Kaiser, J. Knezevic, T. Welte, V. Villa, M. Maruyama, M. Svejda, S. Jahner, F. Fischer, R. Strasser, U. Rant, Protein analysis by time-resolved measurements with an electro-switchable DNA chip. Nat. Commun. 4, 2099 (2013). 23839273 10.1038/ncomms3099 PMC3719012

[40] R. Maffo-Woulefack, N. Leblond-Bourget, B. Douzi, A new twin expands the VirB8-like protein family. Structure 30, 790–792 (2022). 35660242 10.1016/j.str.2022.05.004

[41] V. Kohler, I. Probst, A. Aufschnaiter, S. Buttner, L. Schaden, G. N. Rechberger, G. Koraimann, E. Grohmann, W. Keller, Conjugative type IV secretion in Gram-positive pathogens: TraG, a lytic transglycosylase and endopeptidase, interacts with translocation channel protein TraM. Plasmid 91, 9–18 (2017). 28219792 10.1016/j.plasmid.2017.02.002

[42] W. S. Sun, G. Torrens, J. Ter Beek, F. Cava, R.-A. Berntsson, Breaking barriers: pCF10 type 4 secretion system relies on a self-regulating muramidase to modulate the cell wall. mBio 15, e00488-24 (2024). 38940556 10.1128/mbio.00488-24 PMC11323569

[43] E. Cascales, P. J. Christie, Definition of a bacterial type IV secretion pathway for a DNA substrate. Science 304, 1170–1173 (2004). 15155952 10.1126/science.1095211 PMC3882297

[44] F. Li, C. Alvarez-Martinez, Y. Chen, K. J. Choi, H. J. Yeo, P. J. Christie, *Enterococcus faecalis* PrgJ, a VirB4-like ATPase, mediates pCF10 conjugative transfer through substrate binding. J. Bacteriol. 194, 4041–4051 (2012). 22636769 10.1128/JB.00648-12 PMC3416518

[45] K. Macé, G. Waksman, Cryo-EM structure of a conjugative type IV secretion system suggests a molecular switch regulating pilus biogenesis. EMBO J. 43, 3287–3306 (2024). 38886579 10.1038/s44318-024-00135-z PMC11294453

[46] T. DeWitt, A. D. Grossman, The bifunctional cell wall hydrolase CwlT is needed for conjugation of the integrative and conjugative element ICEBs1 in Bacillus subtilis andB. anthracis. J. Bacteriol. 196, 1588–1596 (2014). 24532767 10.1128/JB.00012-14 PMC3993365

[47] R. Bantwal, T. L. Bannam, C. J. Porter, N. S. Quinsey, D. Lyras, V. Adams, J. I. Rood, The peptidoglycan hydrolase TcpG is required for efficient conjugative transfer of pCW3 in *Clostridium perfringens*. Plasmid 67, 139–147 (2012). 22244927 10.1016/j.plasmid.2011.12.016

[48] C. T. Leonetti, M. A. Hamada, S. J. Laurer, M. P. Broulidakis, K. J. Swerdlow, C. A. Lee, A. D. Grossman, M. B. Berkmen, Critical components of the conjugation machinery of the integrative and conjugative element ICEBs1 of *Bacillus subtilis*. J. Bacteriol. 197, 2558–2567 (2015). 26013486 10.1128/JB.00142-15 PMC4518827

[49] A. Zoued, J. P. Duneau, E. Durand, A. P. Espana, L. Journet, F. Guerlesquin, E. Cascales, Tryptophan-mediated dimerization of the TssL transmembrane anchor is required for type VI secretion system activity. J. Mol. Biol. 430, 987–1003 (2018). 29458124 10.1016/j.jmb.2018.02.008

[50] J. Agirre, M. Atanasova, H. Bagdonas, C. B. Ballard, A. Basle, J. Beilsten-Edmands, R. J. Borges, D. G. Brown, J. J. Burgos-Marmol, J. M. Berrisford, P. S. Bond, I. Caballero, L. Catapano, G. Chojnowski, A. G. Cook, K. D. Cowtan, T. I. Croll, J. E. Debreczeni, N. E. Devenish, E. J. Dodson, T. R. Drevon, P. Emsley, G. Evans, P. R. Evans, M. Fando, J. Foadi, L. Fuentes-Montero, E. F. Garman, M. Gerstel, R. J. Gildea, K. Hatti, M. L. Hekkelman, P. Heuser, S. W. Hoh, M. A. Hough, H. T. Jenkins, E. Jimenez, R. P. Joosten, R. M. Keegan, N. Keep, E. B. Krissinel, P. Kolenko, O. Kovalevskiy, V. S. Lamzin, D. M. Lawson, A. A. Lebedev, A. G. W. Leslie, B. Lohkamp, F. Long, M. Maly, A. J. McCoy, S. J. McNicholas, A. Medina, C. Millan, J. W. Murray, G. N. Murshudov, R. A. Nicholls, M. E. M. Noble, R. Oeffner, N. S. Pannu, J. M. Parkhurst, N. Pearce, J. Pereira, A. Perrakis, H. R. Powell, R. J. Read, D. J. Rigden, W. Rochira, M. Sammito, F. Sanchez Rodriguez, G. M. Sheldrick, K. L. Shelley, F. Simkovic, A. J. Simpkin, P. Skubak, E. Sobolev, R. A. Steiner, K. Stevenson, I. Tews, J. M. H. Thomas, A. Thorn, J. T. Valls, V. Uski, I. Uson, A. Vagin, S. Velankar, M. Vollmar, H. Walden, D. Waterman, K. S. Wilson, M. D. Winn, G. Winter, M. Wojdyr, K. Yamashita, The CCP4 suite: Integrative software for macromolecular crystallography. Acta Crystallogr. D Struct. Biol. 79, 449–461 (2023). 37259835 10.1107/S2059798323003595 PMC10233625

[51] A. Vagin, A. Teplyakov, Molecular replacement with MOLREP. Acta Crystallogr. D Biol. Crystallogr. 66, 22–25 (2010). 20057045 10.1107/S0907444909042589

[52] P. Emsley, B. Lohkamp, W. G. Scott, K. Cowtan, Features and development of Coot. Acta Crystallogr. D Biol. Crystallogr. 66, 486–501 (2010). 20383002 10.1107/S0907444910007493 PMC2852313

[53] D. Liebschner, P. V. Afonine, M. L. Baker, G. Bunkoczi, V. B. Chen, T. I. Croll, B. Hintze, L. W. Hung, S. Jain, A. J. McCoy, N. W. Moriarty, R. D. Oeffner, B. K. Poon, M. G. Prisant, R. J. Read, J. S. Richardson, D. C. Richardson, M. D. Sammito, O. V. Sobolev, D. H. Stockwell, T. C. Terwilliger, A. G. Urzhumtsev, L. L. Videau, C. J. Williams, P. D. Adams, Macromolecular structure determination using x-rays, neutrons and electrons: Recent developments in Phenix. Acta Crystallogr. D Struct. Biol. 75, 861–877 (2019). 31588918 10.1107/S2059798319011471 PMC6778852

[54] M. D. Winn, G. N. Murshudov, M. Z. Papiz, Macromolecular TLS refinement in REFMAC at moderate resolutions. Methods Enzymol. 374, 300–321 (2003). 14696379 10.1016/S0076-6879(03)74014-2

[55] R. Dong, S. Pan, Z. Peng, Y. Zhang, J. Yang, mTM-align: A server for fast protein structure database search and multiple protein structure alignment. Nucleic Acids Res. 46, W380–W386 (2018). 29788129 10.1093/nar/gky430 PMC6030909

[56] C. Magis, J. F. Taly, G. Bussotti, J. M. Chang, P. Di Tommaso, I. Erb, J. Espinosa-Carrasco, C. Notredame, T-Coffee: Tree-based consistency objective function for alignment evaluation. Methods Mol. Biol. 1079, 117–129 (2014). 24170398 10.1007/978-1-62703-646-7_7

[57] K. Tamura, G. Stecher, S. Kumar, MEGA11: Molecular evolutionary genetics analysis version 11. Mol. Biol. Evol. 38, 3022–3027 (2021). 33892491 10.1093/molbev/msab120 PMC8233496

[58] P. Herviou, A. Balvay, D. Bellet, S. Bobet, C. Maudet, J. Staub, M. Alric, N. Leblond-Bourget, C. Delorme, S. Rabot, S. Denis, S. Payot, Transfer of the integrative and conjugative element ICE*St3* of *Streptococcus thermophilus* in physiological conditions mimicking the human digestive ecosystem. Microbiol. Spectr. 11, e0466722 (2023). 36995244 10.1128/spectrum.04667-22 PMC10269554

[59] B. Douzi, N. T. T. Trinh, S. Michel-Souzy, A. Desmyter, G. Ball, P. Barbier, A. Kosta, E. Durand, K. T. Forest, C. Cambillau, A. Roussel, R. Voulhoux, Unraveling the self-assembly of the *Pseudomonas aeruginosa* XcpQ secretin periplasmic domain provides new molecular insights into type II secretion system secreton architecture and dynamics. mBio 8, e01185-17 (2017). 29042493 10.1128/mBio.01185-17 PMC5646246

[60] X. Wang, C. Pineau, S. Gu, N. Guschinskaya, R. W. Pickersgill, V. E. Shevchik, Cysteine scanning mutagenesis and disulfide mapping analysis of arrangement of GspC and GspD protomers within the type 2 secretion system. J. Biol. Chem. 287, 19082–19093 (2012). 22523076 10.1074/jbc.M112.346338 PMC3365941

[61] T. Inoue, M. Forgac, Cysteine-mediated cross-linking indicates that subunit C of the V-ATPase is in close proximity to subunits E and G of the V1 domain and subunit a of the V0 domain. J. Biol. Chem. 280, 27896–27903 (2005). 15951435 10.1074/jbc.M504890200

[62] R. Van der Meeren, Y. Wen, P. Van Gelder, J. Tommassen, B. Devreese, S. N. Savvides, New insights into the assembly of bacterial secretins: Structural studies of the periplasmic domain of XcpQ from Pseudomonas aeruginosa. J. Biol. Chem. 288, 1214–1225 (2013). 23188826 10.1074/jbc.M112.432096 PMC3543004

[63] I. Guilvout, F. Samsudin, R. G. Huber, P. J. Bond, B. Bardiaux, O. Francetic, Membrane platform protein PulF of the Klebsiella type II secretion system forms a trimeric ion channel essential for endopilus assembly and protein secretion. mBio 15, e0142323 (2024). 38063437 10.1128/mbio.01423-23 PMC10790770

[64] G. E. Crooks, G. Hon, J. M. Chandonia, S. E. Brenner, WebLogo: A sequence logo generator. Genome Res. 14, 1188–1190 (2004). 15173120 10.1101/gr.849004 PMC419797

[65] S. Weber, C. Ramirez, W. Doerfler, Signal hotspot mutations in SARS-CoV-2 genomes evolve as the virus spreads and actively replicates in different parts of the world. Virus Res. 289, 198170 (2020). 32979477 10.1016/j.virusres.2020.198170 PMC7513834

[66] N. Dautin, G. Karimova, A. Ullmann, D. Ladant, Sensitive genetic screen for protease activity based on a cyclic AMP signaling cascade in Escherichia coli. J. Bacteriol. 182, 7060–7066 (2000). 11092869 10.1128/jb.182.24.7060-7066.2000 PMC94834

[67] N. A. Treptow, H. A. Shuman, Genetic evidence for substrate and periplasmic-binding-protein recognition by the MalF and MalG proteins, cytoplasmic membrane components of the Escherichia coli maltose transport system. J. Bacteriol. 163, 654–660 (1985). 3894331 10.1128/jb.163.2.654-660.1985 PMC219172

[68] E. Maguin, P. Duwat, T. Hege, D. Ehrlich, A. Gruss, New thermosensitive plasmid for gram-positive bacteria. J. Bacteriol. 174, 5633–5638 (1992). 1324906 10.1128/jb.174.17.5633-5638.1992 PMC206509

[69] L. J. McGuffin, K. Bryson, D. T. Jones, The PSIPRED protein structure prediction server. Bioinformatics 16, 404–405 (2000). 10869041 10.1093/bioinformatics/16.4.404

[70] A. Drozdetskiy, C. Cole, J. Procter, G. J. Barton, JPred4: A protein secondary structure prediction server. Nucleic Acids Res. 43, W389–W394 (2015). 25883141 10.1093/nar/gkv332 PMC4489285

