# Supplementary material for: Elucidating assembly and function of VirB8 cell wall subunits refines the DNA translocation model in Gram-positive T4SSs
Source: Sci Adv. 2025 Jan 22;11(4):eadq5975. doi: 10.1126/sciadv.adq5975 (PMC11753425; doi:10.1126/sciadv.adq5975)
Supplement: Supplementary file 1 — Tables S1 to S4 Figs. S1 to S10 References [file sciadv.adq5975_sm.pdf]

Science Advances
AAAS

Supplementary Materials for

Elucidating assembly and function of VirB8 cell wall subunits refines the DNA translocation model in Gram-positive T4SSs

Robine Maffo-Woulefack *et al.*

Corresponding author: Frédérique Favier, frederique.favier@univ-lorraine.fr;
Nathalie Leblond-Bourget, nathalie.leblond-bourget@univ-lorraine.fr;
Badreddine Douzi, badreddine.douzi@inrae.fr

Sci. Adv. **11**, eadq5975 (2025)
DOI: 10.1126/sciadv.adq5975

This PDF file includes:

Tables S1 to S4
Figs. S1 to S10
References

Supplementary Materials

Table S1. Statistics of data collection and refinement of $OrfG_{Ext}$ and $OrfG_{Ext}$-A204C-A264C models.

	**$OrfG_{Ext}$**	**$OrfG_{Ext}$-A204C-A264C**
**Data collection**		
Space group	$P4_132$	C2
Beamline	Proxima-2A, SOLEIL	MASSIF-3, ESRF
Unit cell parameters (Å, °)	124.9 124.9 124.9 90. 90. 90.	152.7 89.2 98.4 90. 112.3 90.
Wavelength (Å)	0.9801	0.9677
Resolution (Å) [a]	44.17-1.84 (1.88-1.84)	91.05-2.60 (2.72-2.60)
Observations [a]	2,298,975 (155,745)	115,652 (14,913)
Unique reflections [a]	29,747 (2,127)	37,215 (4,577)
R_{merge} [a]	0.093 (1.753)	0.062 (0.665)
R_{meas} [a]	0.094 (1.765)	0.087 (0.933)
$CC_{1/2}$ [a]	1.0 (0.858)	0.995 (0.682)
Mean I/σ [a]	46.2 (3.5)	10.6 (1.8)
Completeness (%) [a]	99.9 (98.7)	98.8 (99.8)
Multiplicity [a]	77.3 (73.2)	3.1 (3.3)
**Model refinement**		
Resolution (Å) [a]	44.17-1.835 (1.835-1.883)	91.05-2.60 (2.668-2.600)
No. of protein residues	240 (1 monomer)	719 (3 monomers)
No. of non-hydrogen prot. atoms	1967	5912
No. of water molecules	214	-
R_{work} [a]	0.183 (0.339)	0.202 (0.343)
R_{free} [a,b]	0.215 (0.337)	0.256 (0.371)
RMSD bonds (Å)	0.017	0.012
RMSD angles (°)	1.712	2.562
Ramachadran plot [c]		
Favored (%)	97.0	93.4
Outliers (%)	0.0	0.14
Rotamer outliers (%)	0.43	6.15
Clashscore (PR) [c]	3.61 (98)	9.68 (98)
MolProbity score (PR)	1.32 (98)	2.52 (81)
Average B (Å^2)	31.5	
Protein	30.7	87.7
Water	39.0	-
PDB code	7pkw	8s7l

[a] Values for the outer shell of data are given in parentheses.
[b] Five percent test set.
[c] From Molprobity. The percentile ranks (PR) for Clashscore and MolProbity score are given in parentheses.

A

Average distance (Å)	OrfG$_{Ext}$	OrfG$_{Ext}$ A204C-A264C	TcpC
D1:D1’	30.0	32.8 ± 0.3	25.4 ± 0.2
D2:D2’	62.7	61.8 ± 0.3	55.8 ± 0.4
D1:D2	52.1	53.7 ± 0.2	46.1 ± 0.4
D1:D2’	23.4	23.6 ± 0.1	22.4 ± 0.2
D1:D2’’	42.5	44.2 ± 0.3	38.1 ± 0.3

B

		OrfG$_{Ext}$-A204C-A264C	TcpC
D1	OrfG$_{Ext}$	0.97 ± 0.01	0.77 ± 0.00
	OrfG$_{Ext}$-A204C-A264C	0.98 ± 0.02	0.77 ± 0.01
	TcpC		0.99 ± 0.01
D1:D1’	OrfG$_{Ext}$	0.87 ± 0.01	0.74 ± 0.00
	OrfG$_{Ext}$-A204C-A264C	0.98 ± 0.00	0.63 ± 0.01
	TcpC		0.99 ± 0.01
D1:D1’:D1’’	OrfG$_{Ext}$	0.88 ± 0.00	0.76 ± 0.00
	OrfG$_{Ext}$-A204C-A264C	0.97 ± 0.00	0.66 ± 0.00
	TcpC		0.99 ± 0.00
D2	OrfG$_{Ext}$	0.99 ± 0.00	0.85 ± 0.00
	OrfG$_{Ext}$-A204C-A264C	0.99 ± 0.00	0.85 ± 0.01
	TcpC		0.99 ± 0.01
D1:D2	OrfG$_{Ext}$	0.89 ± 0.02	0.71 ± 0.03
	OrfG$_{Ext}$-A204C-A264C	0.98 ± 0.01	0.66 ± 0.02
	TcpC		0.98 ± 0.02
D1:D2’	OrfG$_{Ext}$	0.99 ± 0.00	0.85 ± 0.00
	OrfG$_{Ext}$-A204C-A264C	0.99 ± 0.01	0.85 ± 0.01
	TcpC		0.99 ± 0.00

Table S2: Statistics on the structural comparison of the trimers of OrfG$_{Ext}$, OrfG$_{Ext}$-A204C-A264C and TcpC. **(A)**: Distances between domains. **(B)**: TM-scores after domain superimposition by mTM-align [55]. D1 and D2 refer to the domains of the same monomer, a prime is used to distinguish a domain from a neighboring monomer and a double prime for the other monomer. The values are given as the mean and rmsd of all possible equivalent distances, or equivalent TM-scores. Since a crystallographic axis generates the trimer of OrfG, distances are determined as unique values (no rmsd), and no internal TM-score can be calculated. See Methods section for more details.

Table S3. Strains and plasmids

**Strains**		
**Strain**	**Description**	**Reference**
Escherichia coli K12		
DH5α	*F-, Δ(argF-lacZ)U169, phoA, supE44, Δ(lacZ)M15, relA, endA, thi, hsdR*	New England Biolabs
DHT1	F^- *glnV44 recA1 endA1 gyrA96(Nal*^{r}*) thi-1 hsdR17 spoT1 rfbD1 cya-854 ilv-691::Tn10*	[66]
NT326	*araDI39, ΔlacUl69, rpsL, thi, ΔmalE444, recA1*	[67]
BL21 (DE3)	F^- *ompT gal dcm lon hsdS*$_B$(r_B^- m_B^-) λ(DE3)	Invitrogen
BL21 (DE3)-pET28-*trx-orfG*$_{Ext}$	BL21 strain producing OrfG$_{Ext}$	[28]
BL21 (DE3)-pET28-*trx-orfG-A204C*	BL21 strain producing OrfG-A204C	This study
BL21 (DE3)-pET28-*trx-orfG-A264C*	BL21 strain producing OrfG-A264C	This study
BL21 (DE3)-pET28-*trx-orfG-A204C-A264C*	BL21 strain producing OrfG-A204C-264C	This study
BL21 (DE3)-pET28-*trx-orf13*^{WT}	BL21 strain producing Orf13 soluble domain (71-304)	This study
BL21 (DE3)-pET28-*trx-orf13-V164C*	BL21 strain producing Orf13-V164C	This study
BL21 (DE3)-pET28-*trx-orf13-Q280C*	BL21 strain producing Orf13-Q280C	This study
BL21 (DE3)-pET28-*trx-orf13-V164C-Q280C*	BL21 strain producing Orf13-V164C-V280C	This study
Streptococcus thermophilus		
LMG18311	*Streptococcus thermophilus* LMG18311 strain. used as receptor in mating experiments, erythromycin resistance	Laboratory collection
LMG18311-ICE*St3*	*Streptococcus thermophilus* harboring ICE*St3.* Used as a donor strain in mating experiments, chloramphenicol resistance	Laboratory collection
LMG18311-ICESt3*ΔorfG*	LMG18311-ICE*St3* strain deleted for *orfG*	This study
LMG18311-ICESt3::*orfG*^{A204C}	LMG18311-ICE*St3* strain producing OrfG-A204C	This study
LMG18311-ICESt3::*orfG*$^{A204C\text{-}A264C}$	LMG18311-ICE*St3* strain producing OrfG-A264C	This study
LMG18311-ICESt3::*orfG*$^{A204C\text{-}A264C}$	LMG18311-ICE*St3* strain producing OrfG-A204C-A264C	This study
**Plasmids**		
pET28a+	Expression vector, T7 promoter, Kanamycin resistance	[68]
pET28a-*trx*	6His tag-TEV protease cleavage site sequences inserted into pET28a+	Laboratory collection
pET28-*trx::orfG*$_{Ext}$	*orfG*$_{Ext}$ cloned in frame with 6His-TEV in pET28-TRX	[28]
pET28-*trx::orfG-A204C*	*orfG-A204C* cloned in frame with 6His-TEV in pET28-TRX	This study
pET28-*trx::orfG-A264C*	*orfG-A264C* cloned in frame with 6His-TEV in pET28-TRX	This study
pET28-*trx::orfG-A204C-A264C*	*orfG-A204C-A264C* cloned in frame with 6His-TEV in pET28-TRX	This study
pET28-*trx::orf13*	*orf13* region encoding the soluble domain cloned in frame with 6His-TEV in pET28-TRX	This study

pET28-*trx:: orf13-V164C*	*orfG-A264C* cloned in frame with *6His-TEV* in pET28-TRX	This study
pET28-*trx::orf13-Q280C*	*orfG-A204C-A264C* cloned in frame with *6His-TEV* in pET28-TRX	This study
pET28-*trx::orf13-V164C-Q280C*	*Orf13* region encoding the soluble domain cloned in frame with *6His-TEV* in pET28-TRX	This study
pET28::*Up-Dn*	1024-943 bp Up and Dn products of *orfG* cloned in pET28 using XhoI-SacI for Up and NheI-XbaI for Dn	This study
pET28::*Up-orfG-Dn*	*orfG* inserted in pET28-Up-Dn using SacI-NheI	This study
pGHost9	Thermosensitive Ori of pGK12*,* erythromycin resistance	Laboratory collection
pGhost9::*Up-Dn*	*Up-Dn* products of *orfG* subcloned from pET28-*Up-Dn* to pGhost9	This study
pGhost9::*Up-orfG-Dn*	Up-*orfG*-Dn subcloned from pET28-*Up-orfG-Dn* to pGhost9 using ApaI-NotI	This study
pKTop	For membrane topolgy study, expresses PhoA(22-472)-LacZ(4-40) fusion	This study
pKTop::$orfG_{2-37}$	Sequence of *orfG* corresponding to region 1-37 cloned in pKTop in frame with p*hoA-lacZ* using PCR overlap	This study
pKTop::orf_{2-57}	Sequence of *orfG* corresponding to region 1-57 cloned in pKTop in frame with *phoA-lacZ* using PCR overlap	This study
pKTop::$orfG_{2-331}$	Sequence of *orfG* cloned in pKTop in frame with p*hoA-lacZ* using PCR overlap	This study
pccKAN	ToxR-dependent expression of CAT	
pccKAN::$orfG_{2-63}$	pccKAN plasmid encoding for OrfG N-terminal extension including the TMD sequence (2-63) inserted between ToxR and M	This study
pccKAN::$orfG_{17-63}$	pccKAN plasmid encoding for OrfG N-terminal extension excluding the predicted helix alpha including the TMD sequence (17-63) inserted between ToxR and M	This study
pccKAN::$orfG_{35-63}$	pccKAN plasmid encoding for OrfG TMD sequence (35-63) inserted between ToxR and M	This study
18-Pal	*pal* cloned downstream T18 into pEB355	[31]
25-TolB	*tolB* cloned upstream T25 into pEB354	[31]
pUT18C	High copy number vector that encodes the T18 fragment that is fused upstream of the MCS. This vector allows creating in-frame fusions at the C-terminal end of T18. Ampicillin resistance.	Euromedex
pUT18	High copy number vector that encodes the T18 fragment that is fused downstream of the MCS. This vector allows creating in-frame fusions at the N-terminal end of T18. Ampicillin resistance.	Euromedex
pKT25	Low copy number vector that encodes the T25 fragment that is fused upstream of the MCS. This vector allows creating in-frame fusions at the C-terminal end of T25. Ampicillin resistance.	Euromedex
pKTN25	Low copy number vector that encodes the T25 fragment that is fused downstream of the MCS. This vector allows creating in-frame fusions at the N-terminal end of T25. Ampicillin resistance.	Euromedex
pUT18C-*orfG*	*orfG* cloned downstream the region encoding for T18 in pUT18C	This study
pKT25-*orfG*	*orfG* cloned downstream the region encoding for T25 in pKT25	This study
pUT18C-$orfG_{Ext}$	$orfG_{Ext}$ cloned downstream the region encoding for T18 in pUT18C	This study
pUT18-$orfG_{Ext}$	$orfG_{Ext}$ cloned upstream the region encoding for T18 in pUT18C	This study
pKT25-$orfG_{Ext}$	$orfG_{Ext}$ cloned downstream the region encoding for T26 in pUT18C	This study
pKTN25-$orfG_{Ext}$	$orfG_{Ext}$ cloned upstream the region encoding for T25 in pUT18C	This study
pUT18C-$orfG_{D1}$	$orfG_{D1}$ cloned downstream the region encoding for T18 in pUT18C	This study
pUT18-$orfG_{D1}$	$orfG_{D1}$ cloned upstream the region encoding for T18 in pUT18C	This study

pKT25-*orfG*$_{D1}$	*orfG*$_{D1}$ cloned downstream the region encoding for T25 in pUT18C	This study
pKTN25-*orfG*$_{D1}$	*orfG*$_{D1}$ cloned upstream the region encoding for T25 in pUT18C	This study
pUT18C-*orfG*$_{D2}$	*orfG*$_{D2}$ cloned downstream the region encoding for T18 in pUT18C	This study
pUT18-*orfG*$_{D2}$	*orfG*$_{D2}$ cloned upstream the region encoding for T18 in pUT18C	This study
pKT25-*orfG*$_{D2}$	*orfG*$_{D2}$ cloned downstream the region encoding for T25 in pUT18C	This study
pKTN25-*orfG*$_{D2}$	*orfG*$_{D2}$ cloned upstream the region encoding for T25 in pUT18C	This study

Table S4. List of the oligonucleotides and DNA sequences used in this study

Name	Sequence	Description
**Topology assay**		
OrfG-pKTOP-F	ATTACGCCAAGCTTGCATGCCATAAAAGCTAAACAATACTTAC	Forward primer to amplify the sequence encoding for OrfG
OrfG-37-R	ATCCTCTAGAGTCGACCTGCAGGTCTTTTTAGTAGTGACTTT	Reverse primer to amplify the sequence encoding for OrfG1-37
OrfG-63-R	ATCCTCTAGAGTCGACCTGCAGAGCGTGATAGCACGGATA	Reverse primer to amplify the sequence encoding for OrfG1-63
OrfG-331-R	ATCCTCTAGAGTCGACCTGCAATCTTTATCGTTTGCGTAG	Reverse primer to amplify the sequence encoding for OrfG1-331 (full-length)
**BACTH assay**		
T18/T25-OrfG-F	CCCCCTGCAGCTATAAAAGCTAAACAATACTTAC	Forward primer to amplify the sequence encoding for full-length OrfG
T18/T25-OrfG-R	CCCCGGATCCCTAATCTTTATCGTTTGCGTAG	Reverse primer to amplify the sequence encoding for full-length OrfG
T18/T25-OrfG$_{Ext}$-F	CCCCCTGCAGCTTCAAATAAAGTGGGAACACTAC	Forward primer to amplify the sequence encoding for OrfG$_{Ext}$
T18/T25-OrfG$_{Ext}$-R	CCCCGGATCCCTAATCTTTATCGTTTGCGTAG	Reverse primer to amplify the sequence encoding for OrfG$_{Ext}$
OrfG$_{Ext}$-T18/T25-F	CCCCAAGCTTGTCAAATAAAGTGGGAACACTAC	Forward primer to amplify the sequence encoding for OrfG$_{Ext}$
OrfG$_{Ext}$-T18/T25-R	CCCCGGATCCGCATCTTTATCGTTTGCGTAGTTG	Reverse primer to amplify the sequence encoding for OrfG$_{Ext}$
OrfG$_{D1}$-T18/T25-F	CCCCCTGCAGTTCAAATAAAGTGGGAACACTAC	Forward primer to amplify the sequence encoding for OrfG$_{D1}$
OrfG$_{D1}$-T18/T25-R	CCCCCCCGGGGTTCGCTAAAGTATCCAGC	Reverse primer to amplify the sequence encoding for OrfG$_{D1}$
T18/T25-OrfG$_{D2}$-F	CCCCCTGCAGTGCCAACGACCACGTCTCAG	Forward primer to amplify the sequence encoding for OrfG$_{D2}$
T18/T25-OrfG$_{D2}$-R	CCCCCCCGGGGATCTTTATCGTTTGCGTAGTTG	Reverse primer to amplify the sequence encoding for OrfG$_{D2}$
**TOXCAT assay**		
OrfG$_{2\text{-}63}$-TOXCAT-F	GGTGGTGCTAGCATAAAAGCTAAACAATACTTACTA	Forward primer to amplify the sequence encoding for OrfG N-terminal extension variants
OrfG$_{17\text{-}63}$-T-F	GGTGGTGCTAGCAATTTTAAAAAGGTGGATAAAAAAGG	Forward primer to amplify the sequence encoding for OrfG$_{17\text{-}63}$
OrfG$_{35\text{-}63}$-TOXCAT-F	GGTGGTGCTAGCAAAAAGACCGTCAACATAGC	Forward primer to amplify the sequence encoding for OrfG$_{35\text{-}63}$
OrfG$_{2\text{-}63}$-TOXCAT-R	GGTGGTGGATCCCGAGCGTGATAGCACGGATAG	Reverse primer to amplify the sequence encoding for OrfG N-terminal extension variants
***orfG* knock-out mutant**		
ΔG1-F	GGGCGGCCGCAACTCTCAACCAGTCCACAG	Forward primer to amplify the OrfG Up region for cloning into pGhost9
ΔG2-R	GCTCTAAAAGGAGGACCTATG	Reverse primer to amplify the OrfG Up region for cloning into pGhost9

ΔG3-F	GCTCTAAAAGGAGGACCTATGAATACAGAC AATCTACGTACCTTCC	Forward primer to amplify the OrfG Dn region for cloning into pGhost9
ΔG4-R	GAACTGGGAGAAGAACTCCTGGGCCCCG	Reverse primer to amplify the OrfG Dn region for cloning into pGhost9
**Insertion of *orfG* variant to native locus of *orfG* in *ΔorfG* strain**		
Sther-G-Up-F	AATTCTCGAGAACTCTCAACCAGTCCACAG	Forward primer to amplify the OrfG Up region for cloning into pET28
Sther-G-Up-R	AATTGAGCTCTCCTCCTTTTAGAGACTAGCG	Reverse primer to amplify the OrfG Up region for cloning into pET28
Sther-G-Dn-F	AATTGCTAGCATGAATACAGACAATCTACGT ACC	Forward primer to amplify the OrfG Up region for cloning into pET28
Sther-G-Dn-R	AATTTCTAGAAGGAGTTCTTCTCCCAGTTC	Reverse primer to amplify the OrfG Up region for cloning into pET28
Sther-G-F	AATTGAGCTCATGATAAAAGCTAAACAATAC TTAC	Forward primer to amplify *orfG* or *orfG* cysteine variants for cloning into pET28::Up-Dn
Sther-G-R	AATTGCTAGCAGTTCTCCTAATCTTTATCGTT TGCGTAG	Reverse primer to amplify *orfG* or *orfG* cysteine variants for cloning into pET28::Up-Dn
Sther-GΔTM -F	AATTGAGCTCATGTCAAATAAAGTGGGAAC ACTAC	Forward primer to amplify *orfG* region encoding for *OrfG*ΔTM (residues 64-331)
**Cysteine mutants**		
K90C-F	AATCGCTGCTATGACTACAAAC	Forward primer used for the K90C variant construction
K90C-R	GTCATAGCAGCGATTAGCGTCT	Reverse primer used for the K90C variant construction
Y91C-F	CGCAAGTGTGACTACAAACTCC	Forward primer used for the Y91C variant construction
Y91C-R	GTAGTCACACTTGCGATTAGCG	Reverse primer used for the Y91C variant construction
K134C-F	GACGTTTGTGCACAAGGTCAAG	Forward primer used for the K134C variant construction
K134C-R	TTGTGCACAAACGTCTGGAACG	Reverse primer used for the K134C variant construction
V153C-F	GACTCACAATTAGTCACGTGTGAAGGTAAG GTAGCTACC	Forward primer used for the V154C variant construction
V153-R	GGTAGCTACCTTACCTTCACACGTGACTAAT TGTGAGTC	Reverse primer used for the V154C variant construction
E154C-F2	ACGGTATGTGGTAAGGTAGCTAC	Forward primer used for the E154C variant construction
E154C-R2	CTTACCACATACCGTGACTAATTG	Reverse primer used for the E154C variant construction
K193C-F	CCTTTGATGAAAAAGAGGGGTGCTACTACG TTTCTGGACTTCC	Forward primer used for the K193C variant construction
K193C-R	GGAAGTCCAGAAACGTAGTAGCACCCCTCT TTTTCATCAAAGG	Reverse primer used for the K193C variant construction
A204C-F	TTCTCATGTATTGAGTCTTCAC	Forward primer used for the A204C variant construction
A204C-R	CTCAATACATGAGAACCAAGGA	Reverse primer used for the A204C variant construction
A210C-F	GCGATTGAGTCTTCACAATGTGGATACTTTA GCGAAGAC	Forward primer used for the A210C variant construction
A210C-R	GTCTTCGCTAAAGTATCCACATTGTGAAGAC TCAATCGC	Reverse primer used for the A210C variant construction

A264C-F	GCTAAGAACGTTGCTATCGTATGCAATACCACCTTTAAAACGATTGAC	Forward primer used for the A264C variant construction
A264C-R	GTCAATCGTTTTAAAGGTGGTATTGCATACGATAGCAACGTTCTTAGC	Reverse primer used for the A264C variant construction
D272C-F	CCAATACCACCTTTAAAACGATTTGCTACACCTATCTTAAGAAAGATAGG	Forward primer used for the D272C variant construction
D272C-R	CCTATCTTTCTTAAGATAGGTGTAGCAAATCGTTTTAAAGGTGGTATTGG	Reverse primer used for the D272C variant construction
**switchSENSE**		
ssOV	GAGACTTCAACCCCCGATTTCTAATAGGGGGGTTACATTTTCAAGATTTAGAAAGTGTGTCACTTTGGTCCAAAAAGTGTGTCACTTAGTCAAAAGGAGATAT**ATCAGCGTTCGATGCTTCCGACTAATCAGCCATATCAGCTTACGACTA**	Oligonucleotide composed by the complementary sequence of the Nanolever (in bold) called c-NV and the single strand DNA sequence originated from ICE*St3* (underlined) called ssDNA.
c-DNA	CTCCTTTTGACTAAGTGACACACTTTTTGGACCAAAGTGACACACTTTCTAAATCTTGAAAATGTAACCCCCCTATTAGAAATCGGGGGTTGAAGTCTC	Overhang complementary sequence to ssOV (sequence underlined in ssOV) originated from ICESt3 in order to create a double stranded Overhang
ssOV-NS	ATGCGTAGCTGTACACGGTATGATCCAGTTCCGTAGGTACGTCATTCGTCGTATCATGTAGCATGTCACGAGTCTCTATGGTGGATCACTGGTCATGTTATAT**ATCAGCGTTCGATGCTTCCGACTAATCAGCCATATCAGCTTACGACTA**	Control oligonucleotide composed by the complementary sequence of the Nanolever (in bold) called C-NV and the non-specific (NS) Overhang DNA sequence (underlined) called ssDNA-NS
c-DNA-NS	AACATGACCAGTGATCCACCATAGAGACTCGTGACATGCTACATGATACGACGAATGACGTACCTACGGAACTGGATCATACCGTGTACAGCTACGCAT	Non-specific overhang complementary sequence in order to create non-specific dsOverhang
***In vitro* chemical crosslinking**		
ssDNA*	GAGACTTCAACCCCCGATTTCTAATAGGGGGGTTACATTTTCAAGATTTAGAAAGTGTGTCACTTTGGTCCAAAAAGTGTGTCACTTAGTCAAAAGGAG	Sequence corresponding to the ssDNA fragment used for switchSENSE experiment labeled with 6-FAM at 5' end.

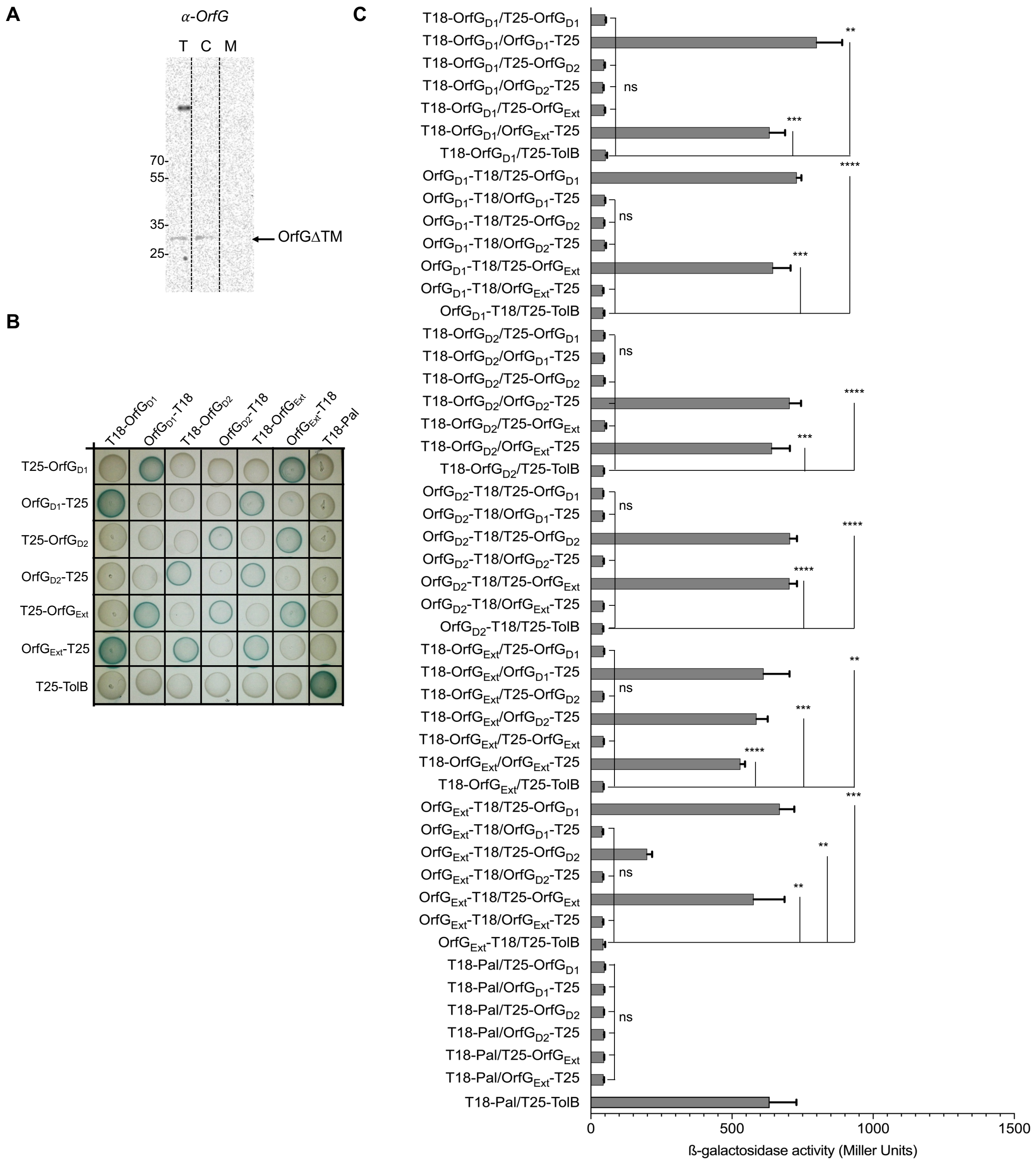

A
α-OrfG
T C M
70-
55-
35-
25-
OrfGΔTM
B
T18-OrfGD1
OrfGD1-T18
T18-OrfGD2
OrfGD2-T18
T18-OrfGExt
OrfGExt-T18
T18-Pal
T25-OrfGD1
OrfGD1-T25
T25-OrfGD2
OrfGD2-T25
T25-OrfGExt
OrfGExt-T25
T25-TolB
C
T18-OrfGD1/T25-OrfGD1
T18-OrfGD1/OrfGD1-T25
T18-OrfGD1/T25-OrfGD2
T18-OrfGD1/OrfGD2-T25
T18-OrfGD1/T25-OrfGExt
T18-OrfGD1/OrfGExt-T25
T18-OrfGD1/T25-TolB
OrfGD1-T18/T25-OrfGD1
OrfGD1-T18/OrfGD1-T25
OrfGD1-T18/T25-OrfGD2
OrfGD1-T18/OrfGD2-T25
OrfGD1-T18/T25-OrfGExt
OrfGD1-T18/OrfGExt-T25
OrfGD1-T18/T25-TolB
T18-OrfGD2/T25-OrfGD1
T18-OrfGD2/OrfGD1-T25
T18-OrfGD2/T25-OrfGD2
T18-OrfGD2/OrfGD2-T25
T18-OrfGD2/T25-OrfGExt
T18-OrfGD2/OrfGExt-T25
T18-OrfGD2/T25-TolB
OrfGD2-T18/T25-OrfGD1
OrfGD2-T18/OrfGD1-T25
OrfGD2-T18/T25-OrfGD2
OrfGD2-T18/OrfGD2-T25
OrfGD2-T18/T25-OrfGExt
OrfGD2-T18/OrfGExt-T25
OrfGD2-T18/T25-TolB
T18-OrfGExt/T25-OrfGD1
T18-OrfGExt/OrfGD1-T25
T18-OrfGExt/T25-OrfGD2
T18-OrfGExt/OrfGD2-T25
T18-OrfGExt/T25-OrfGExt
T18-OrfGExt/OrfGExt-T25
T18-OrfGExt/T25-TolB
OrfGExt-T18/T25-OrfGD1
OrfGExt-T18/OrfGD1-T25
OrfGExt-T18/T25-OrfGD2
OrfGExt-T18/OrfGD2-T25
OrfGExt-T18/T25-OrfGExt
OrfGExt-T18/OrfGExt-T25
OrfGExt-T18/T25-TolB
T18-Pal/T25-OrfGD1
T18-Pal/OrfGD1-T25
T18-Pal/T25-OrfGD2
T18-Pal/OrfGD2-T25
T18-Pal/T25-OrfGExt
T18-Pal/OrfGExt-T25
T18-Pal/T25-TolB
ns
**
***
****
0
500
1000
1500
ß-galactosidase activity (Miller Units)

D

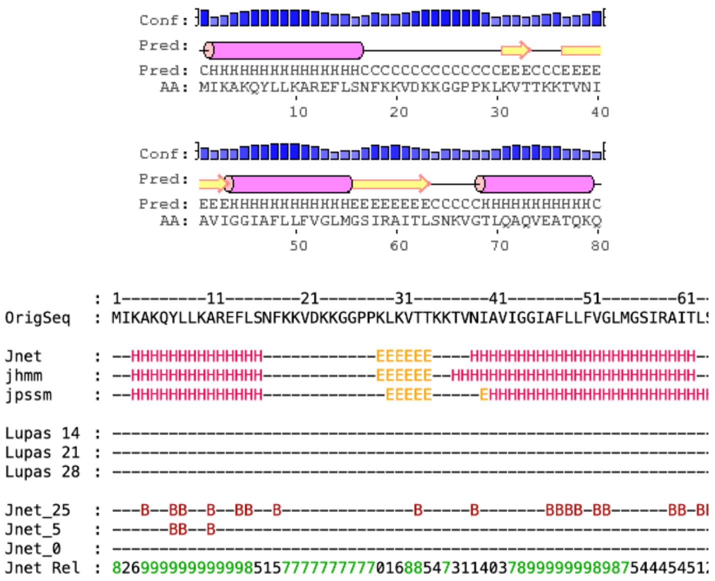

E

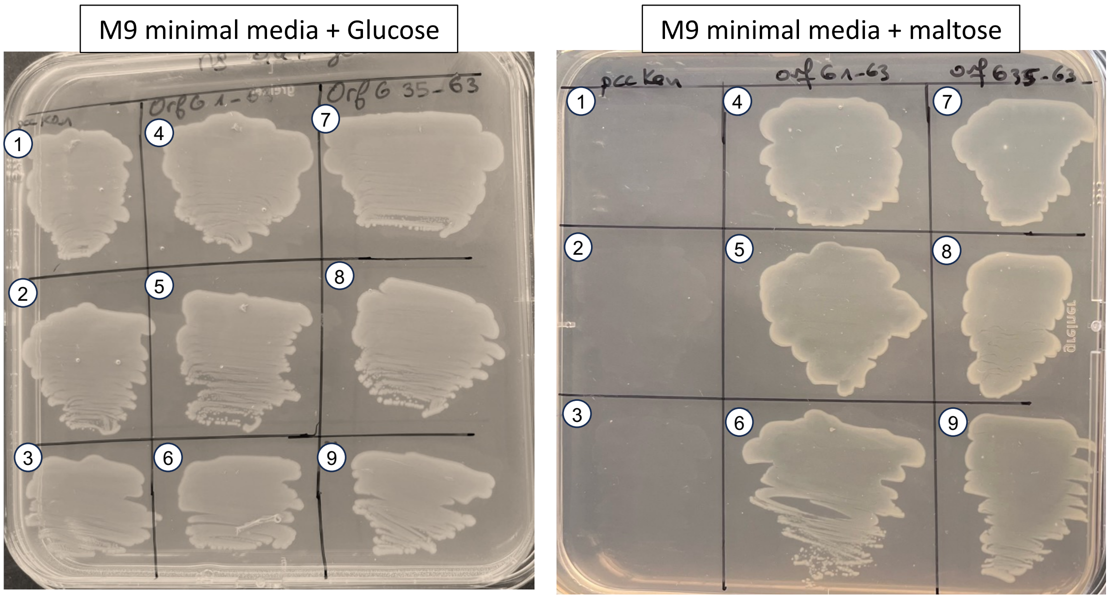

Figure S1: Exploration of the cellular localization of OrfG and its self-assembly. (A) Analysis of the cellular localization of OrfGΔTM in *S. thermophilus*. Immunoblotting of the total (T), cytoplasmic (C) and membrane (M) fractions of the strain producing OrfGΔTM using polyclonal Ab-G. Molecular mass markers (kDa) are indicated on the left. **(B,C)** BACTH analysis of OrfG interacting domains. Functional complementation resulting from the interaction between the indicated hybrid proteins was monitored by measuring phenotypic assay using the chromogenic substrate X-Gal **(B)** or by monitoring the β-galactosidase activity **(C)**. **(B)** Phenotypic analysis of OrfG interacting domains using LB plates supplemented with the chromogenic substrate X-Gal. Relevant positive interactions along with selected negative controls were extracted from the plate and presented in Figure 3A, right panel. **(C)** β-galactosidase activities represent the analyzed binary interactions between D1, D2 and

$OrfG_{Ext}$. The binary interacting partners are mentioned at the left. The β-galactosidase activities are presented in histograms. The statistical analyses were conducted by comparing interactions of interest to internal negative controls. Bars and whiskers denote mean values and standard errors of the mean; ns, not significant ($P \geq 0.05$), **P* <0 .05, ***P* < 0.01, *** *P* < 0.001 and **** P<0.0001. **(D)** Secondary structure predictions of OrfG N-terminal region including the cytoplasmic extension and the TMD of OrfG using Psipred [69] (top panel) and JPred [70] (bottom panel). **(E)** NT326 reporter cells producing ToxR-X-MalE fusions (X corresponds to the region encoded by the empty vector (pccKAN), OrfG1-63 or $OrfG_{35-63}$) were streaked on M9 minimal medium supplemented with glucose (right panel) or maltose (left panel) as sole carbon source. Colonies 1-3, 4-6 and 7-9 corresponds to three different sets of colonies: colonies containing the empty vector, colonies expressing the $orfG_{1-63}$ variant and colonies expressing the $orfG_{35-63}$ variant, respectively.

A

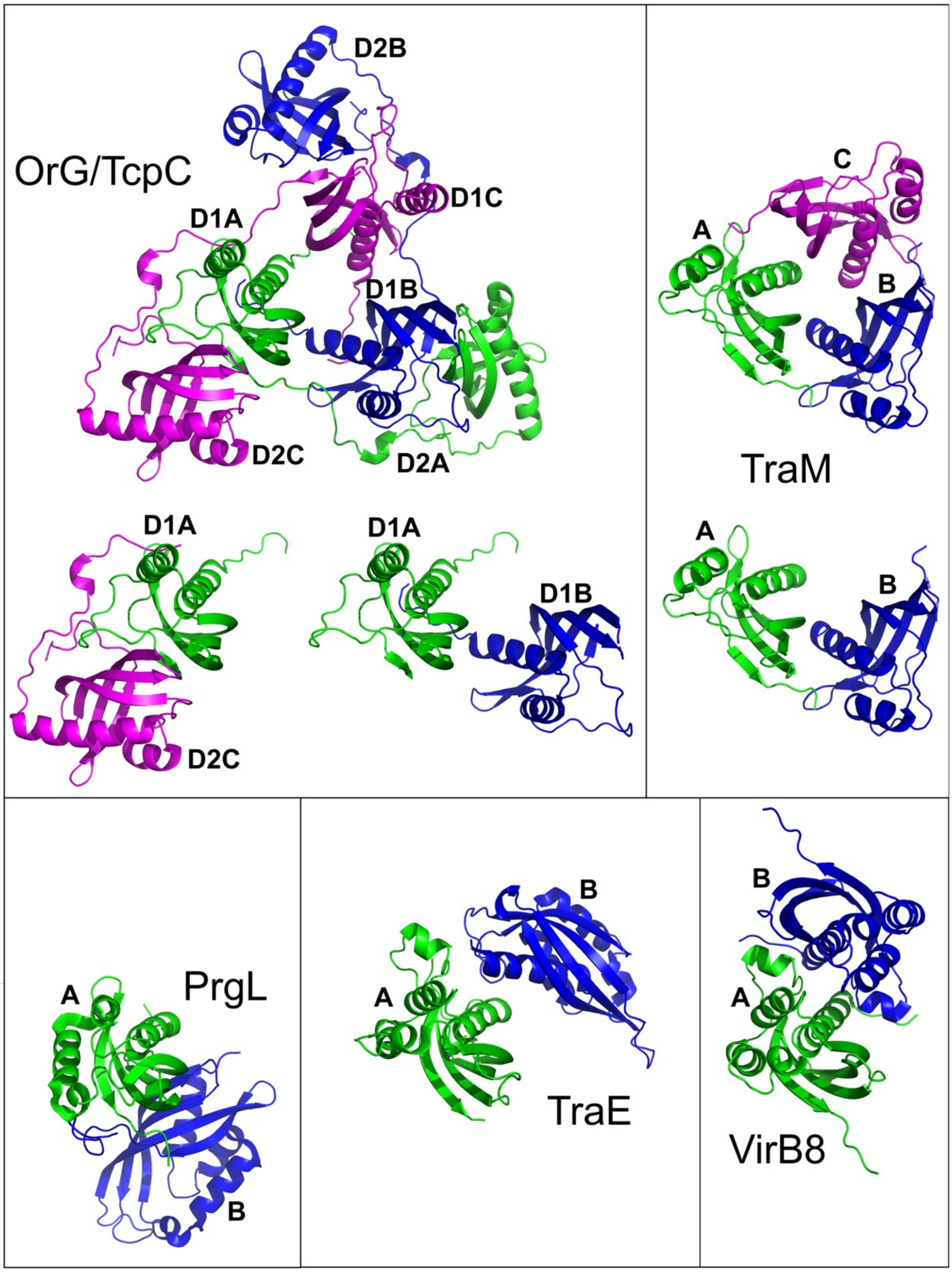

OrG/TcpC
D2B
D1C
D1A
D1B
D2C
D2A
D1A
D2C
D1A
D1B
TraM
C
A
B
A
B
PrgL
A
B
TraE
A
B
VirB8
B
A

B

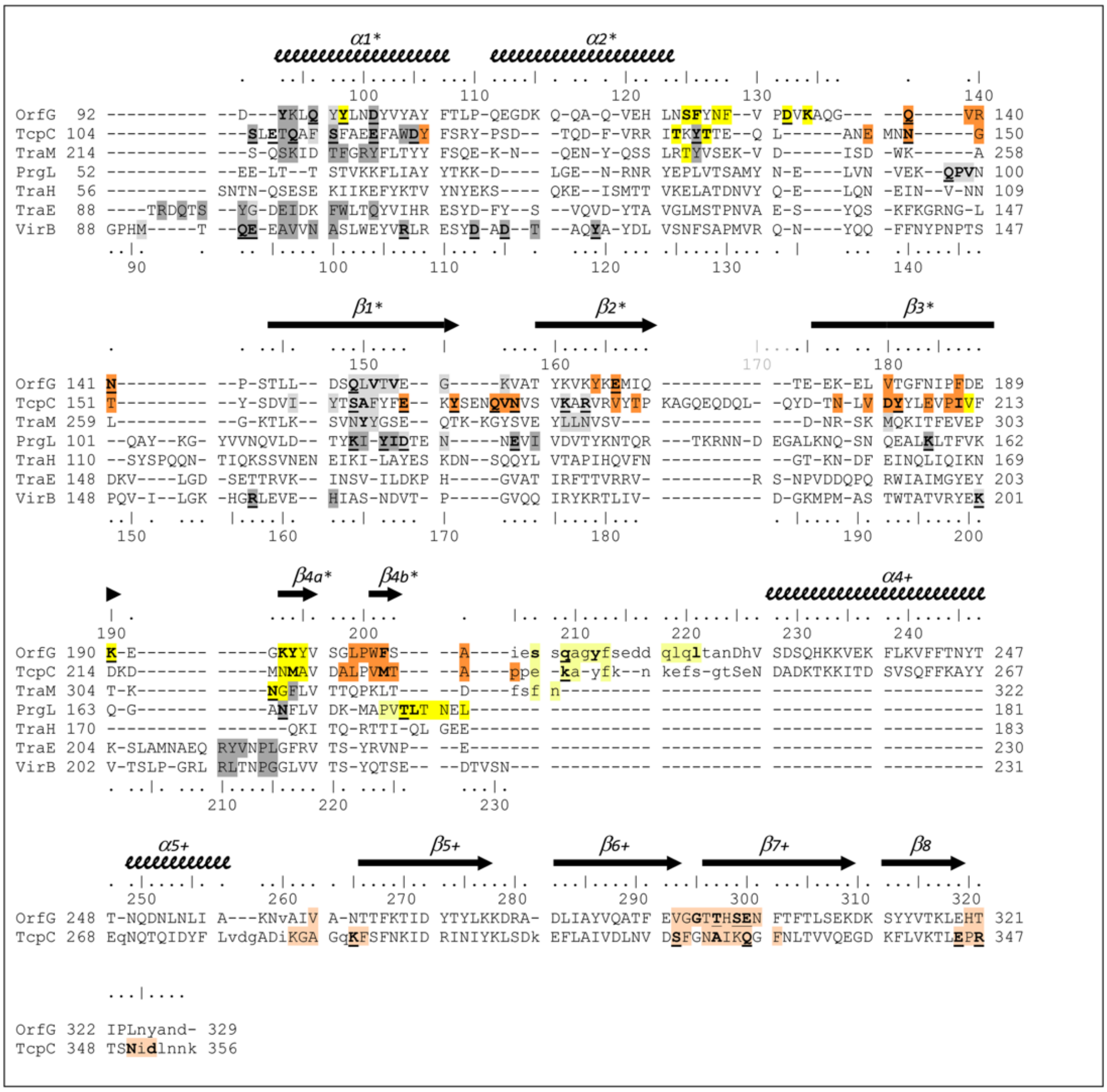

Figure S2: Structural comparison of Gram-positive VirB8-like and Gram-negative VirB8 structures. (A) Cartoon views to highlight the diversity of oligomerization interfaces. Top left: trimer of OrfG from *S. thermophilus* ICE*St3* (pdb 7pkw) and TcpC from *C. perfringens* (pdb 3ub1), two types of interfaces D1:D2' (i.e. D1A:D2C or D1B:D2A or D1C:D2B) and D1:D1' (i.e. D1A-B1B or D1A-D1C or D1B-D1C); Top right: trimer of TraM from *Enterococcus faecalis* pIP501 (pdb 4ec6), one type of interface A-A' (i.e. A:B or A:C or B:C); Bottom left: dimer of PrgL from *E. faecalis* pCF10 (pdb 7aed); Bottom center: dimer of Gram-negative TraE from *E. coli* pKM101 (pdb 5i97); Bottom right: dimer of Gram-negative VirB8 from *A. tumefaciens* (2cc3). **(B)** Structure-based sequence alignment of Gram-positive OrfG, TcpC, TraM, PrgL, TraH from *E. faecalis* pIP501 (pdb 5aiw), TraE and VirB8 generated by mTM-align [55] and manually modified to add the mTM-alignment of domains D2 of both OrfG and TcpC. mTM-align does not show residues that are not superimposed in the structure (for instance the C-ter residues 182-189 of PrgL). Lower case letters correspond to the residues of the D1-D2 linkers that do not superimpose in OrfG and TcpC. Numbering above the sequences corresponds to OrfG, under the sequences to VirB8 from *A. tumefaciens*. Secondary structures of OrfG are

represented by arrows (β-strands) and squiggles (α-helices). Their names are followed by a star (or a cross) when they belong to the domain D1 (or D2, respectively). Residues found at a distance of less than 4 Å in trimeric assemblies are highlighted: grey corresponds to the D1-D1 interface (dark grey for D1A, light grey in the neighboring D1B or equivalent), yellow is for the interactions of the linker of monomer A (light yellow) with domain D1B or equivalent (dark yellow) and orange corresponds to the interface formed between D1A (dark orange) with D2C (light orange) or equivalent. Interface residues that form hydrogen bonds are shown in bold type, and are underlined if their side chain is involved. Interactions mediated through water molecules were not considered. No interactions are shown for TraH since the structure was determined by NMR.

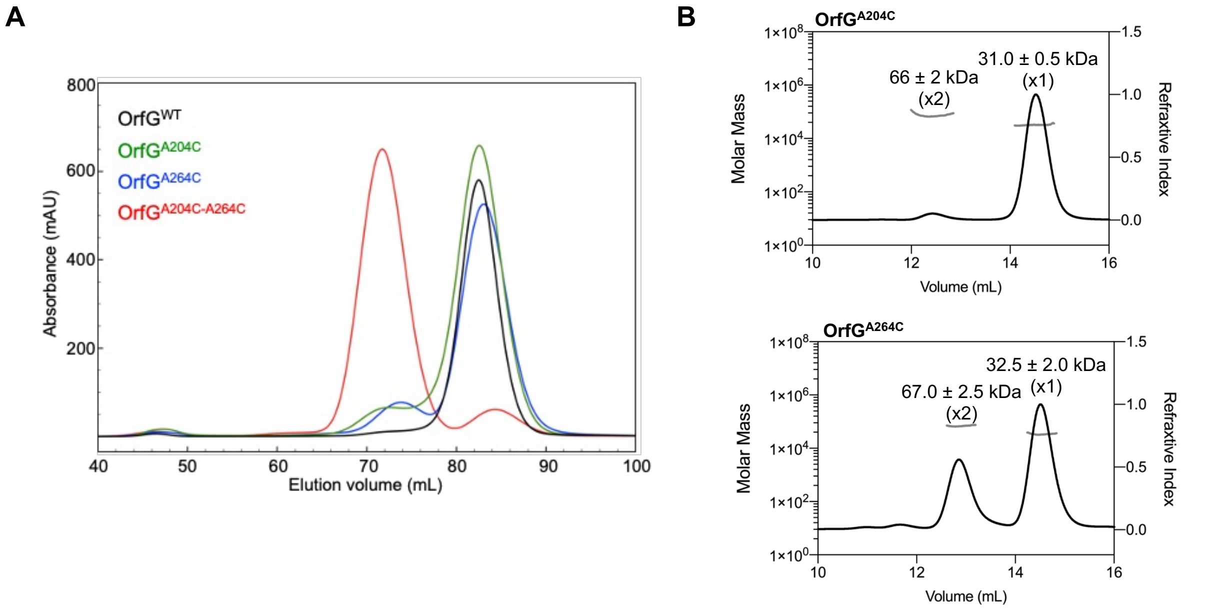

Figure S3: Biochemical analysis OrfG^{A204C} (OrfG$_{Ext}$-A204C) and OrfG^{A264C} (OrfG$_{Ext}$-A264C). (A) Superposition of size exclusion chromatograms of OrfG^{WT} (OrfG$_{Ext,}$ in black), OrfG-A204C (OrfG^{A204C} in green) and OrfG-A264C (OrfG^{A264C} in blue) and OrfG$_{Ext}$-A204-A264C (OrfG$^{A204C-A264C}$ in red). **(B)** SEC-MALS analysis of OrfG$_{Ext}$-A204C and OrfG$_{Ext}$-A264C. The elution profile (black line) is shown with the molecular weight calculated by MALS (gray lines). The elution volume (mL) is plotted on the first y-axis and the refractive index is plotted on the second y-axis. All molecular weights indicated at the top of each peak represent the average molecular weight and standard deviation from 3 independent measurements.

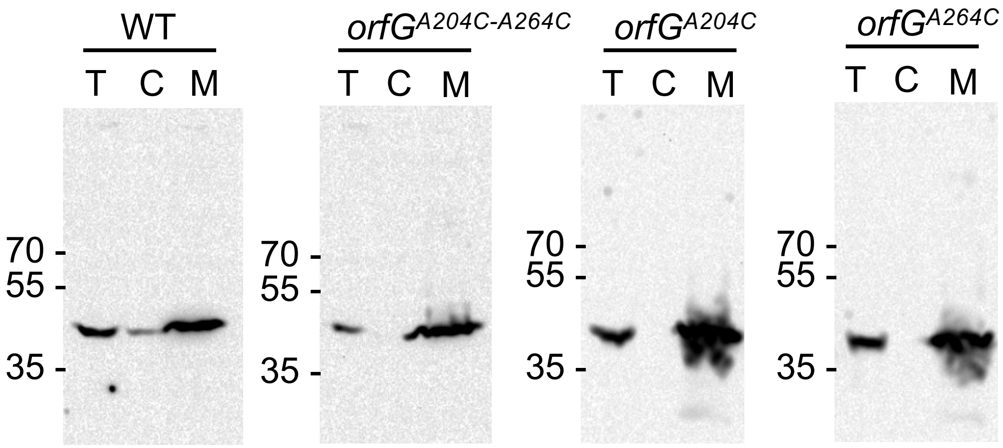

Figure S4: **Analysis of the cellular localization of OrfG and OrfG cysteine variants in *S. thermophilus*.** Immunoblotting of the total (T), cytoplasmic (C) and membrane (M) fractions of WT strain and *ΔorfG* strain producing OrfG-A204C-A264C (*orfG*$^{A204C-A264C}$), OrfG-A204C (*orfG*^{A204C}) and OrfG-A264C (*orfG*^{A264C}) using polyclonal Ab-G. Molecular mass markers (kDa) are indicated on the left.

VirB8-like protein	WP013904640.1	WP155124617.1	CAC67546.1	WP247938026.1	WP003034679.1	WP011285001.1	WP011285001.1	WPA76983.1	WP061574046.1	ABF47327.1	PZT49213.1	ADX78817.1	AAK17951.1	CBG92860.1	ACW84394.1	QKS89574.1	AAB60020.1	ACC59235.1
WP013904640.1_ICESparas15912rpmG *Streptococcus parasanguinis*	100	97	88	73	50	30	30	14	13	12	13	15	15	11	14	13	14	14
WP155124617.1_Conjugative element *Streptococcus parasanguinis*	97	100	88	75	50	29	29	14	13	13	13	14	14	11	14	13	14	14
CAC67546.1_ICESt3_*Streptococcus thermophilus*	88	88	100	73	52	28	28	14	13	12	12	16	16	11	14	13	14	14
WP247938026.1_Conjugative_element_*Streptococcus gordonii*	73	75	73	100	49	29	29	17	15	13	13	14	13	12	13	13	13	13
WP003034679.1_ICE SanC238 tRNAleu *Streptococcus anginosus*	50	50	52	49	100	24	24	15	15	11	11	14	14	13	12	12	12	12
WP011285001.1_ICESpy6180tRNAthr_*Streptococcus pyogenes*	30	29	28	29	24	100	100	19	18	10	10	15	15	15	18	18	18	18
WP011285001.1_ICESdy2713tRNAthr_*Streptococcus dygalactiae*	30	29	28	29	24	100	100	19	18	10	10	15	15	15	18	18	18	18
WPA76983.1_ICEBs1_*Bacillus subtilis*	14	14	14	17	15	19	19	100	72	14	14	15	15	17	16	15	16	16
WP061574046.1_Conjugative_element_*Bacillus amyloliquefaciens*	13	13	13	15	15	18	18	72	100	14	14	17	17	16	17	16	17	17
ABF47327.1_pCW3_*Clostridium perfringens*	12	13	12	13	11	10	10	14	14	100	98	17	17	16	17	17	17	17
PZT49213.1_Conjugative_element_*Clostridium perfringens*	13	13	12	13	11	10	10	14	14	98	100	17	17	16	17	17	17	17
ADX78817.1_Conjugative element *Enterococcus faecalis*	15	14	16	14	14	15	15	15	17	17	17	100	98	47	52	50	52	52
AAK17951.1_CW459tet_M_*Clostridium perfringens*	15	14	16	13	14	15	15	15	17	17	17	98	100	47	52	50	52	52
CBG92860.1_Tn6000_*Enterococcus casseliflavus*	11	11	11	12	13	15	15	17	16	16	16	47	47	100	45	43	45	45
ACW84394.1_Tn5251_*Streptococcus pneumoniae*	14	14	14	13	12	18	18	16	17	17	17	52	52	45	100	95	100	100
QKS89574.1_Conjugative_element_*Streptococcus pneumoniae*	13	13	13	13	12	18	18	15	16	17	17	50	50	43	95	100	95	95
AAB60020.1_Tn916_*Enterococcus faecalis*	14	14	14	13	12	18	18	16	17	17	17	52	52	45	100	95	100	100
ACC59235.1_Tn5253_*Streptococcus pneumoniae*	14	14	14	13	12	18	18	16	17	17	17	52	52	45	100	95	100	100

Figure S5: Analysis of sequence identity among VirB8-like proteins. The table displays the percentage of sequence identity among multiple VirB8-like proteins from β-class allowing their classification into five distinct families. Each family is represented by a different color in the table. Different colors are attributed to each family.

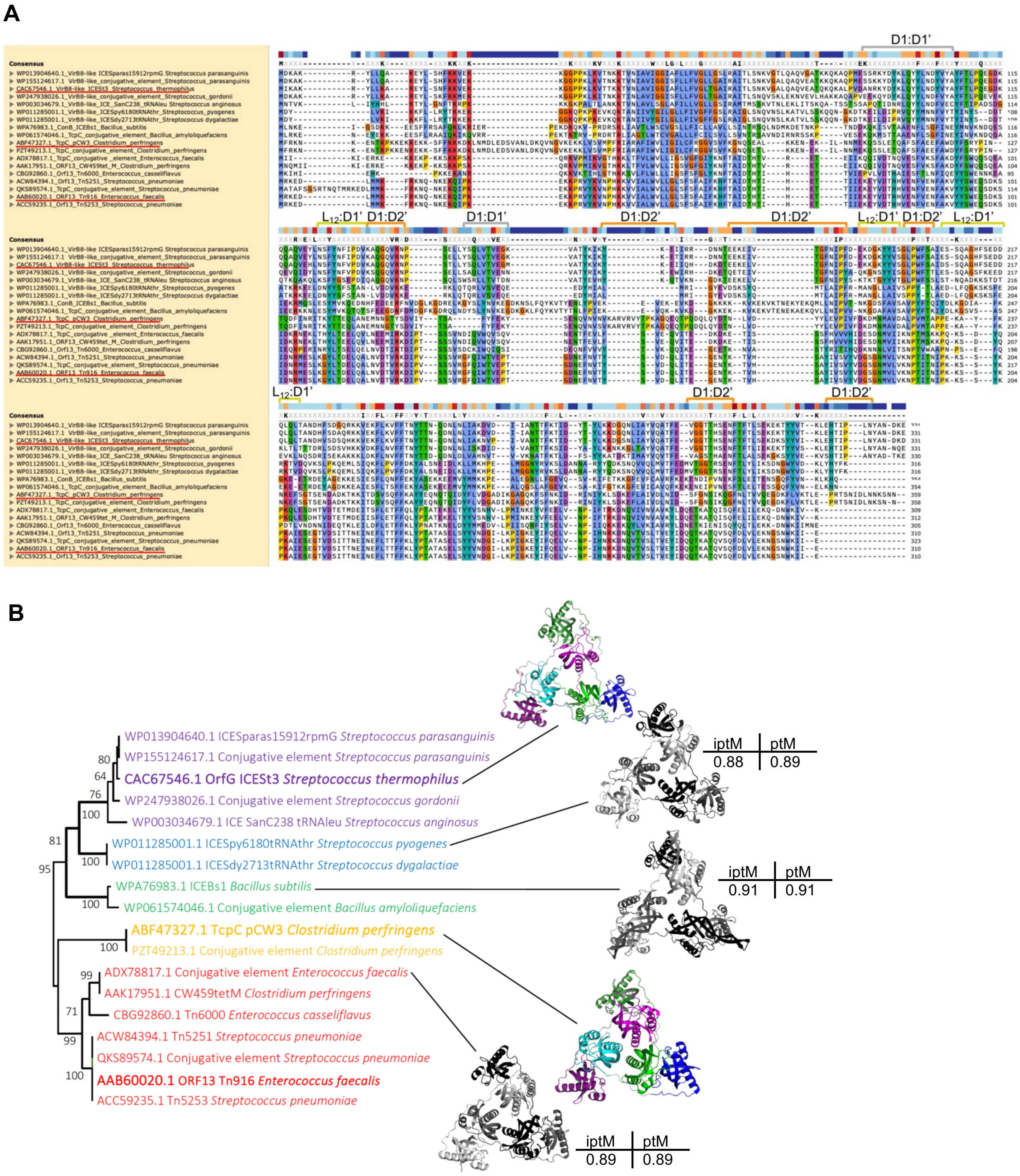

Figure S6: VirB8-like proteins display high sequence diversity but share a common assembly pattern. (A) Multiple sequence alignment of VirB8-like proteins from β-class. Snapgene [65] was used to visualize the multiple sequence alignment. The sequences corresponding to OrfG, TcpC and Orf13 are underlined. The regions of OrfG and TcpC corresponding to the interfaces D1:D1', L_{12}:D1' or D1:D2' in the structures of the trimers are delimited by brackets colored according to Figure 4E. When conflicts exist (a same zone involved in interactions of different types), the given information corresponds to OrfG. The residues are colored according to the default color scheme used for ClustalX. In blue, hydrophobic residues: A, I, L, M, F, W, V. In red, positively charged residues: K, R. In magenta, the negatively charged residues: E, D. In green, polar residues: N, Q, S, T. In orange, G. In yellow, P. In cyan, H and Y and in white, non-conserved residues. **(B)** The AlphaFold 3 models of representative proteins from each identified family of VirB8-like proteins (the phylogenetic tree shown here is the one used in

Figure 7A). The experimentally determined structure of OrfG and TcpC are colored. The AlphaFold 3 models are displayed in gray. Confidence scores, including the predicted TM score (pTM) and the interface pTM score (ipTM) provided by Alphafold-multimer for the top ranked model, are listed alongside the corresponding model**.**

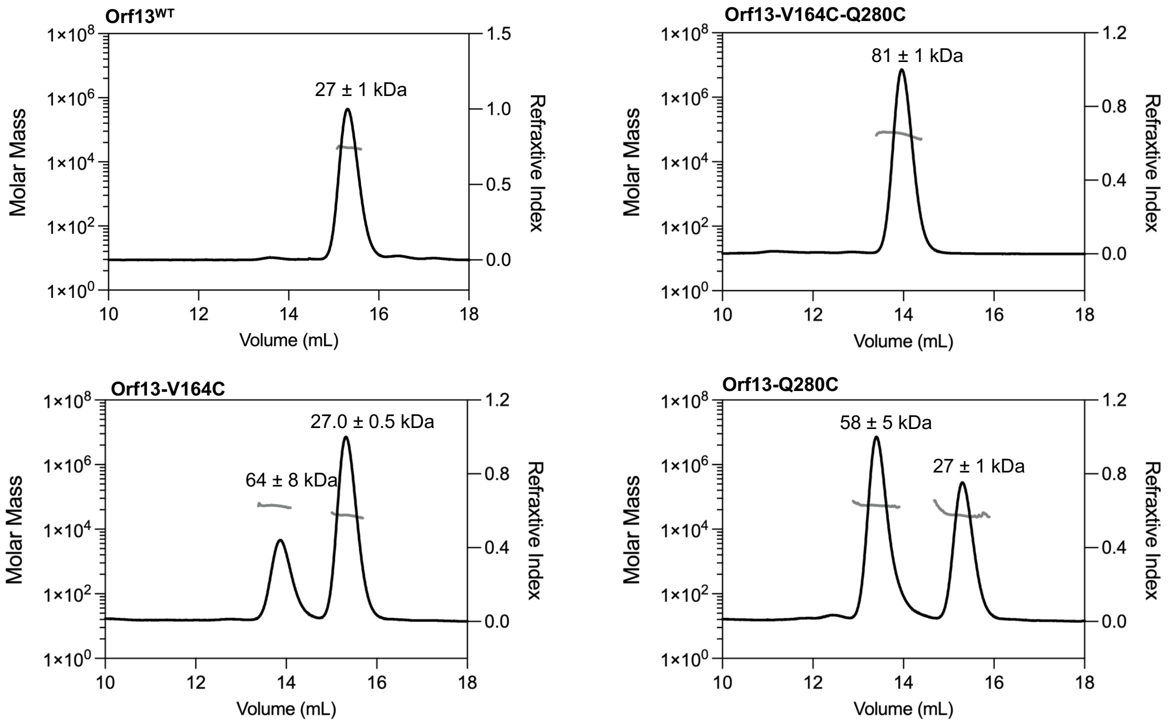

Figure S7: SEC-MALS analysis of Orf13^{WT} and the simple and double cysteine variants. The elution profile (black line) is shown with the molecular weight calculated by MALS (gray lines). The elution volume (mL) is plotted on the first y-axis and the refractive index is plotted on the second y-axis. All molecular weights indicated at the top of each peak represent the average molecular weight and standard deviation from 3 independent measurements.

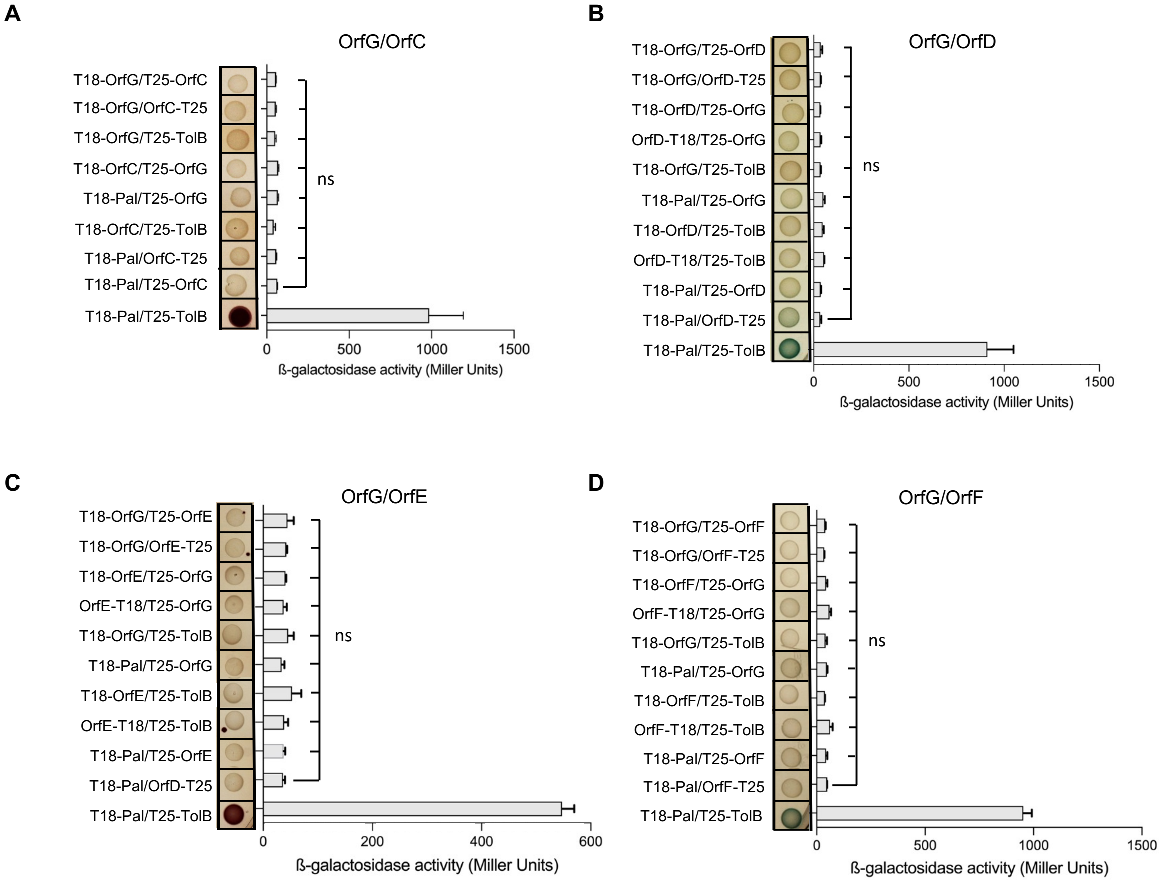

Figure S8: Mapping the interaction of OrfG with T4SS channel subunits. Monitoring of the interactions between OrfG and OrfC **(A)**, OrfD **(B)**, OrfE **(C)** and OrfF **(D)** using BACTH. Functional complementation resulting from the interaction between the indicated hybrid proteins was examined by measuring the β-galactosidase activity and phenotypic assays using LB agar supplemented with X-Gal or using MacConkey agar plates. The β-galactosidase activities are presented in histograms. The statistical analyses were conducted by comparing interactions of interest to internal negative controls. Bars and whiskers denote mean values and standard errors of the mean; ns, not significant.

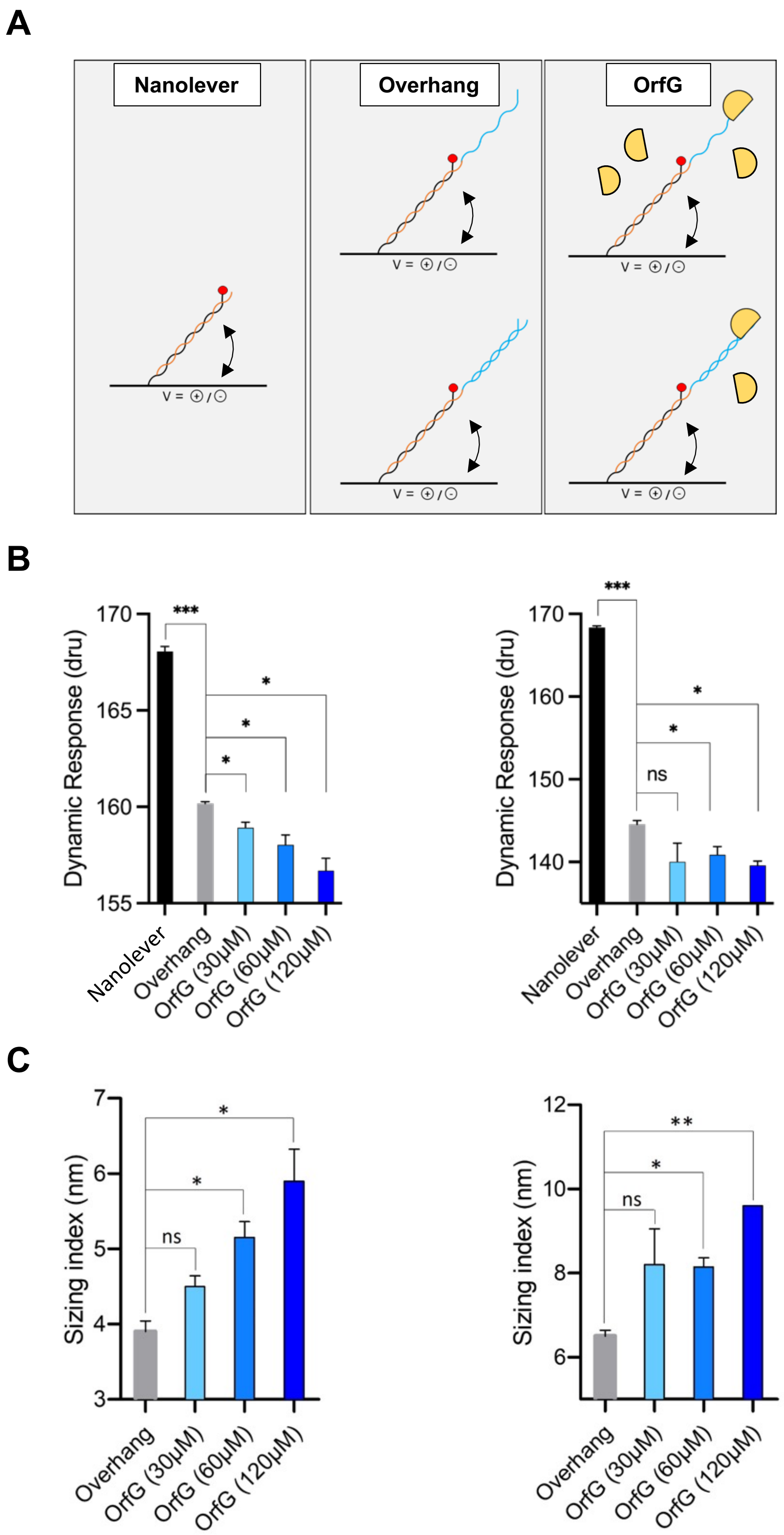

Figure S9: Investigation of OrfG-DNA interaction using non-specific DNA sequence. (A) Schematic representation of the molecular dynamics system. The overhangs, contains a ss- or dsDNA region of ICE*St3* (blue) (listed in Table S4), and a ssDNA sequence (orange) that is complementary to the tethered Nanolever (black). The Nanolever has a fluorophore linked at its end (red circle). Alternating the electric potentials applied to the gold microchip surface will either attract or repel the negatively charged dsDNA. Real-time monitoring of the fluorescence emission of the dye attached to one of the DNA strands depends on its proximity to the gold

surface and allowing the accurate measurement of the oscillating orientation change (between + and -) of the DNA in dynamic response, over a time interval (between 0 and 10 μs). So, the ligand (DNA) and/or the analyte (protein) binding to the DNA strand complementary to the tethered DNA which carrying the fluorophore, potentially influences the dynamic response and it can be deduced in two ways: conformational change analysis, which provides the dynamic response unit (dru), and the florescence restoration time or sizing analysis, which provides the apparent hydrodynamic diameter (sizing index). Conformation **(B)** and **(C)** sizing analysis of OrfG binding to nonspecific DNA. ssDNA in left panels and dsDNA in right panels. The results are represented by plotting the used analyte (purified recombined $OrfG_{Ext}$ (OrfG) or controls (Nanolevers or/and Overhang)) in the x-axis and the dynamic response unit and sizing index in y-axis.

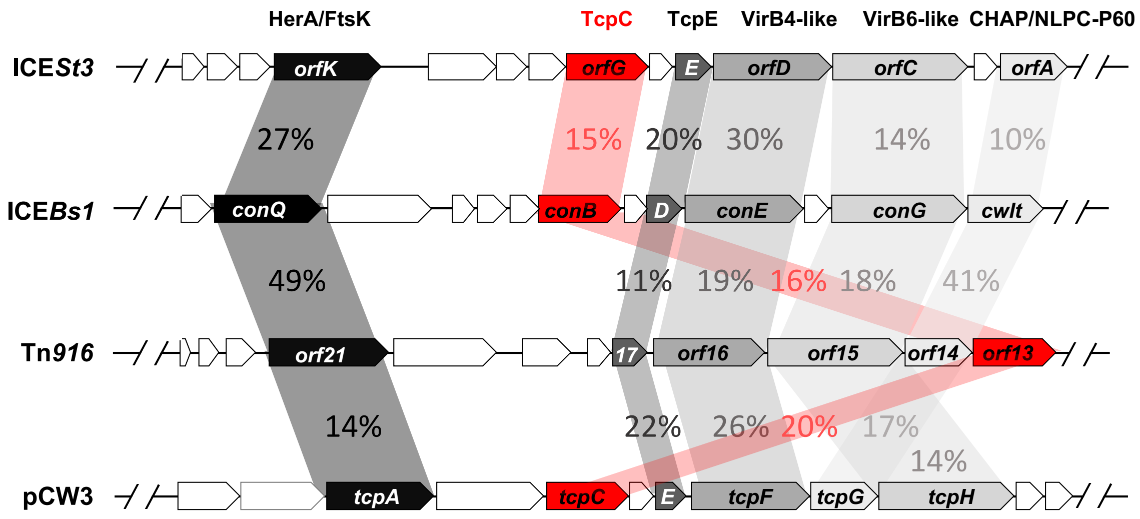

Figure S10: Sequence conservation across conserved proteins within MPF$_{FA}$ clade. The corresponding protein family names for each T4SS are indicated at the top. Percentage of sequence identity between two analyzed sequences is indicated. The colors used correspond to those used in Figure 1.

REFERENCES AND NOTES

1. S. M. Soucy, J. Huang, J. P. Gogarten, Horizontal gene transfer: Building the web of life. *Nat. Rev. Genet.* **16**, 472–82 (2015).

2. E. Grohmann, P. J. Christie, G. Waksman, S. Backert, Type IV secretion in Gram-negative and Gram-positive bacteria. *Mol. Microbiol.* **107**, 455–471 (2018).

3. X. Bellanger, S. Payot, N. Leblond-Bourget, G. Guedon, Conjugative and mobilizable genomic islands in bacteria: Evolution and diversity. *FEMS Microbiol. Rev.* **38**, 720–760 (2014).

4. S. R. Partridge, S. M. Kwong, N. Firth, S. O. Jensen, Mobile genetic elements associated with antimicrobial resistance. *Clin. Microbiol. Rev.* **31**, e00088-17 (2018).

5. T. R. D. Costa, J. B. Patkowski, K. Macé, P. J. Christie, G. Waksman, Structural and functional diversity of type IV secretion systems. *Nat. Rev. Microbiol.* **22**, 170–185 (2024).

6. E. Cascales, P. J. Christie, The versatile bacterial type IV secretion systems. *Nat. Rev. Microbiol.* **1**, 137–149 (2003).

7. C. E. Alvarez-Martinez, P. J. Christie, Biological diversity of prokaryotic type IV secretion systems. *Microbiol. Mol. Biol. Rev.* **73**, 775–808 (2009).

8. R. Fronzes, P. J. Christie, G. Waksman, The structural biology of type IV secretion systems. *Nat. Rev. Microbiol.* **7**, 703–714 (2009).

9. M. Bhatty, J. A. Laverde Gomez, P. J. Christie, The expanding bacterial type IV secretion lexicon. *Res. Microbiol.* **164**, 620–639 (2013).

10. P. J. Christie, The mosaic type IV secretion systems. *EcoSal Plus* **7**, 10.1128/ecosalplus.esp-0020-2015 (2016).

11. T. R. D. Costa, L. Harb, P. Khara, L. Zeng, B. Hu, P. J. Christie, Type IV secretion systems: Advances in structure, function, and activation. *Mol. Microbiol.* **115**, 436–452 (2021).

12. K. Mace, A. K. Vadakkepat, A. Redzej, N. Lukoyanova, C. Oomen, N. Braun, M. Ukleja, F. Lu, T. R. D. Costa, E. V. Orlova, D. Baker, Q. Cong, G. Waksman, Cryo-EM structure of a type IV secretion system. *Nature* **607**, 191–196 (2022).

13. M. J. Sheedlo, M. D. Ohi, D. B. Lacy, T. L. Cover, Molecular architecture of bacterial type IV secretion systems. *PLOS Pathog.* **18**, e1010720 (2022).

14. D. Chetrit, B. Hu, P. J. Christie, C. R. Roy, J. Liu, A unique cytoplasmic ATPase complex defines the *Legionella pneumophila* type IV secretion channel. *Nat. Microbiol.* **3**, 678–686 (2018).

15. B. Hu, P. Khara, L. Song, A. S. Lin, A. E. Frick-Cheng, M. L. Harvey, T. L. Cover, P. J. Christie, In situ molecular architecture of the *Helicobacter pylori* Cag type IV secretion system. *mBio* **10**, 10.1128/mbio.00849-19 (2019).

16. P. Khara, L. Song, P. J. Christie, B. Hu, In situ visualization of the pKM101-encoded type IV secretion system reveals a highly symmetric ATPase energy center. *mBio* **12**, e0246521 (2021).

17. X. Liu, P. Khara, M. L. Baker, P. J. Christie, B. Hu, Structure of a type IV secretion system core complex encoded by multi-drug resistance F plasmids. *Nat. Commun.* **13**, 379 (2022).

18. D. Ghosal, K. C. Jeong, Y. W. Chang, J. Gyore, L. Teng, A. Gardner, J. P. Vogel, G. J. Jensen, Molecular architecture, polar targeting and biogenesis of the *Legionella* Dot/Icm T4SS. *Nat. Microbiol.* **4**, 1173–1182 (2019).

19. J. Guglielmini, L. Quintais, M. P. Garcillan-Barcia, F. de la Cruz, E. P. Rocha, The repertoire of ICE in prokaryotes underscores the unity, diversity, and ubiquity of conjugation. *PLOS Genet.* **7**, e1002222 (2011).

20. J. Guglielmini, F. de la Cruz, E. P. Rocha, Evolution of conjugation and type IV secretion systems. *Mol. Biol. Evol.* **30**, 315–331 (2013).

21. N. Goessweiner-Mohr, K. Arends, W. Keller, E. Grohmann, Conjugative type IV secretion systems in Gram-positive bacteria. *Plasmid* **70**, 289–302 (2013).

22. F. Jager, A. Lamy, W. S. Sun, N. Guerini, R. P. Berntsson, Structure of the enterococcal T4SS protein PrgL reveals unique dimerization interface in the VirB8 protein family. *Structure* **30**, 876–885.e5 (2022).

23. C. J. Porter, R. Bantwal, T. L. Bannam, C. J. Rosado, M. C. Pearce, V. Adams, D. Lyras, J. C. Whisstock, J. I. Rood, The conjugation protein TcpC from *Clostridium perfringens* is structurally related to the type IV secretion system protein VirB8 from Gram-negative bacteria. *Mol. Microbiol.* **83**, 275–88 (2012).

24. N. Goessweiner-Mohr, L. Grumet, K. Arends, T. Pavkov-Keller, C. C. Gruber, K. Gruber, R. Birner-Gruenberger, A. Kropec-Huebner, J. Huebner, E. Grohmann, W. Keller, The 2.5 Å structure of the *Enterococcus* conjugation protein TraM resembles VirB8 type IV secretion proteins. *J. Biol. Chem.* **288**, 2018–2028 (2013).

25. C. Fercher, I. Probst, V. Kohler, N. Goessweiner-Mohr, K. Arends, E. Grohmann, K. Zangger, N. H. Meyer, W. Keller, VirB8-like protein TraH is crucial for DNA transfer in Enterococcus faecalis. *Sci. Rep.* **6**, 24643 (2016).

26. C. Baron, VirB8: A conserved type IV secretion system assembly factor and drug target. *Biochem. Cell Biol.* **84**, 890–899 (2006).

27. L. Dobson, I. Reményi, G. E. Tusnády, CCTOP: A consensus constrained TOPology prediction web server. *Nucleic Acids Res.* **43**, W408–W412 (2015).

28. J. Cappele, A. Mohamad Ali, N. Leblond-Bourget, S. Mathiot, T. Dhalleine, S. Payot, M. Savko, C. Didierjean, F. Favier, B. Douzi, Structural and biochemical analysis of OrfG: The VirB8-like component of the conjugative type IV secretion system of ICE *St3* from *Streptococcus thermophilus*. *Front. Mol. Biosci.* **8**, 642606 (2021).

29. G. Karimova, D. Ladant, Defining membrane protein topology using pho-lac reporter fusions. *Methods Mol. Biol.* **1615**, 129–142 (2017).

30. F. Romero-Saavedra, D. Laverde, E. Kalfopoulou, C. Martini, R. Torelli, D. Martinez-Matamoros, M. Sanguinetti, J. Huebner, Conjugation of different immunogenic enterococcal vaccine target antigens leads to extended strain coverage. *J. Infect. Dis.* **220**, 1589–1598 (2019).

31. A. Battesti, E. Bouveret, The bacterial two-hybrid system based on adenylate cyclase reconstitution in *Escherichia coli*. *Methods* **58**, 325–334 (2012).

32. W. P. Russ, D. M. Engelman, TOXCAT: A measure of transmembrane helix association in a biological membrane. *Proc. Natl. Acad. Sci. U.S.A.* **96**, 863–868 (1999).

33. L. Holm, C. Sander, Dali: A network tool for protein structure comparison. *Trends Biochem. Sci.* **20**, 478–480 (1995).

34. Y. Zhang, J. Skolnick, TM-align: A protein structure alignment algorithm based on the TM-score. *Nucleic Acids Res.* **33**, 2302–2309 (2005).

35. E. Krissinel, K. Henrick, Inference of macromolecular assemblies from crystalline state. *J. Mol. Biol.* **372**, 774–797 (2007).

36. E. Garriga, P. Di Tommaso, C. Magis, I. Erb, L. Mansouri, A. Baltzis, E. Floden, C. Notredame, Multiple sequence alignment computation using the T-coffee regressive algorithm implementation. *Methods Mol. Biol.* **2231**, 89–97 (2021).

37. J. Jumper, R. Evans, A. Pritzel, T. Green, M. Figurnov, O. Ronneberger, K. Tunyasuvunakool, R. Bates, A. Zidek, A. Potapenko, A. Bridgland, C. Meyer, S. A. A. Kohl, A. J. Ballard, A. Cowie, B. Romera-Paredes, S. Nikolov, R. Jain, J. Adler, T. Back, S. Petersen, D. Reiman, E. Clancy, M. Zielinski, M. Steinegger, M. Pacholska, T. Berghammer, S. Bodenstein, D. Silver, O. Vinyals, A. W. Senior, K. Kavukcuoglu, P. Kohli, D. Hassabis, Highly accurate protein structure prediction with AlphaFold. *Nature* **596**, 583–589 (2021).

38. J. M. Auchtung, N. Aleksanyan, A. Bulku, M. B. Berkmen, Biology of ICE*Bs1*, an integrative and conjugative element in *Bacillus subtilis*. *Plasmid* **86**, 14–25 (2016).

39. A. Langer, P. A. Hampel, W. Kaiser, J. Knezevic, T. Welte, V. Villa, M. Maruyama, M. Svejda, S. Jahner, F. Fischer, R. Strasser, U. Rant, Protein analysis by time-resolved measurements with an electro-switchable DNA chip. *Nat. Commun.* **4**, 2099 (2013).

40. R. Maffo-Woulefack, N. Leblond-Bourget, B. Douzi, A new twin expands the VirB8-like protein family. *Structure* **30**, 790–792 (2022).

41. V. Kohler, I. Probst, A. Aufschnaiter, S. Buttner, L. Schaden, G. N. Rechberger, G. Koraimann, E. Grohmann, W. Keller, Conjugative type IV secretion in Gram-positive pathogens: TraG, a lytic transglycosylase and endopeptidase, interacts with translocation channel protein TraM. *Plasmid* **91**, 9–18 (2017).

42. W. S. Sun, G. Torrens, J. Ter Beek, F. Cava, R.-A. Berntsson, Breaking barriers: pCF10 type 4 secretion system relies on a self-regulating muramidase to modulate the cell wall. *mBio* **15**, e00488-24 (2024).

43. E. Cascales, P. J. Christie, Definition of a bacterial type IV secretion pathway for a DNA substrate. *Science* **304**, 1170–1173 (2004).

44. F. Li, C. Alvarez-Martinez, Y. Chen, K. J. Choi, H. J. Yeo, P. J. Christie, *Enterococcus faecalis* PrgJ, a VirB4-like ATPase, mediates pCF10 conjugative transfer through substrate binding. *J. Bacteriol.* **194**, 4041–4051 (2012).

45. K. Macé, G. Waksman, Cryo-EM structure of a conjugative type IV secretion system suggests a molecular switch regulating pilus biogenesis. *EMBO J.* **43**, 3287–3306 (2024).

46. T. DeWitt, A. D. Grossman, The bifunctional cell wall hydrolase CwlT is needed for conjugation of the integrative and conjugative element ICEBs1 in Bacillus subtilis and B. anthracis. *J. Bacteriol.* **196**, 1588–1596 (2014).

47. R. Bantwal, T. L. Bannam, C. J. Porter, N. S. Quinsey, D. Lyras, V. Adams, J. I. Rood, The peptidoglycan hydrolase TcpG is required for efficient conjugative transfer of pCW3 in *Clostridium perfringens*. *Plasmid* **67**, 139–147 (2012).

48. C. T. Leonetti, M. A. Hamada, S. J. Laurer, M. P. Broulidakis, K. J. Swerdlow, C. A. Lee, A. D. Grossman , M. B. Berkmen, Critical components of the conjugation machinery of the integrative and conjugative element ICEBs1 of *Bacillus subtilis*. *J. Bacteriol.* **197**, 2558–2567 (2015).

49. A. Zoued, J. P. Duneau, E. Durand, A. P. Espana, L. Journet, F. Guerlesquin, E. Cascales, Tryptophan-mediated dimerization of the TssL transmembrane anchor is required for type VI secretion system activity. *J. Mol. Biol.* **430**, 987–1003 (2018).

50. J. Agirre, M. Atanasova, H. Bagdonas, C. B. Ballard, A. Basle, J. Beilsten-Edmands, R. J. Borges, D. G. Brown, J. J. Burgos-Marmol, J. M. Berrisford, P. S. Bond, I. Caballero, L. Catapano, G. Chojnowski, A. G. Cook, K. D. Cowtan, T. I. Croll, J. E. Debreczeni, N. E. Devenish, E. J. Dodson, T. R. Drevon, P. Emsley, G. Evans, P. R. Evans, M. Fando, J. Foadi, L. Fuentes-Montero, E. F. Garman, M. Gerstel, R. J. Gildea, K. Hatti, M. L. Hekkelman, P. Heuser, S. W. Hoh, M. A. Hough, H. T. Jenkins, E. Jimenez, R. P. Joosten, R. M. Keegan, N. Keep, E. B. Krissinel, P. Kolenko, O. Kovalevskiy, V. S. Lamzin, D. M. Lawson, A. A. Lebedev, A. G. W. Leslie, B. Lohkamp, F. Long, M. Maly, A. J. McCoy, S. J. McNicholas, A. Medina, C. Millan, J. W. Murray, G. N. Murshudov, R. A. Nicholls, M. E. M. Noble, R. Oeffner, N. S. Pannu, J. M. Parkhurst, N. Pearce, J. Pereira, A. Perrakis, H. R. Powell, R. J. Read, D. J. Rigden, W. Rochira, M. Sammito, F. Sanchez Rodriguez, G. M. Sheldrick, K. L. Shelley, F. Simkovic, A. J. Simpkin, P. Skubak, E. Sobolev, R. A. Steiner, K. Stevenson, I. Tews, J. M. H. Thomas, A. Thorn, J. T. Valls, V. Uski, I. Uson, A. Vagin, S. Velankar, M. Vollmar, H. Walden, D. Waterman, K. S. Wilson, M. D. Winn, G. Winter, M. Wojdyr, K. Yamashita, The CCP4 suite: Integrative software for macromolecular crystallography. *Acta Crystallogr. D Struct. Biol.* **79**, 449–461 (2023).

51. A. Vagin, A. Teplyakov, Molecular replacement with MOLREP. *Acta Crystallogr. D Biol. Crystallogr.* **66**, 22–25 (2010).

52. P. Emsley, B. Lohkamp, W. G. Scott, K. Cowtan, Features and development of Coot. *Acta Crystallogr. D Biol. Crystallogr.* **66**, 486–501 (2010).

53. D. Liebschner, P. V. Afonine, M. L. Baker, G. Bunkoczi, V. B. Chen, T. I. Croll, B. Hintze, L. W. Hung, S. Jain, A. J. McCoy, N. W. Moriarty, R. D. Oeffner, B. K. Poon, M. G. Prisant, R. J.

Read, J. S. Richardson, D. C. Richardson, M. D. Sammito, O. V. Sobolev, D. H. Stockwell, T. C. Terwilliger, A. G. Urzhumtsev, L. L. Videau, C. J. Williams, P. D. Adams, Macromolecular structure determination using x-rays, neutrons and electrons: Recent developments in Phenix. *Acta Crystallogr. D Struct. Biol.* **75**, 861–877 (2019).

54. M. D. Winn, G. N. Murshudov, M. Z. Papiz, Macromolecular TLS refinement in REFMAC at moderate resolutions. *Methods Enzymol.* **374**, 300–321 (2003).

55. R. Dong, S. Pan, Z. Peng, Y. Zhang, J. Yang, mTM-align: A server for fast protein structure database search and multiple protein structure alignment. *Nucleic Acids Res.* **46**, W380–W386 (2018).

56. C. Magis, J. F. Taly, G. Bussotti, J. M. Chang, P. Di Tommaso, I. Erb, J. Espinosa-Carrasco, C. Notredame, T-Coffee: Tree-based consistency objective function for alignment evaluation. *Methods Mol. Biol.* **1079**, 117–129 (2014).

57. K. Tamura, G. Stecher, S. Kumar, MEGA11: Molecular evolutionary genetics analysis version 11. *Mol. Biol. Evol.* **38**, 3022–3027 (2021).

58. P. Herviou, A. Balvay, D. Bellet, S. Bobet, C. Maudet, J. Staub, M. Alric, N. Leblond-Bourget, C. Delorme, S. Rabot, S. Denis, S. Payot, Transfer of the integrative and conjugative element ICE*St3* of *Streptococcus thermophilus* in physiological conditions mimicking the human digestive ecosystem. *Microbiol. Spectr.* **11**, e0466722 (2023).

59. Douzi, B., Trinh, N. T. T., Michel-Souzy, S., Desmyter, A., Ball, G., Barbier, P., Kosta, A., Durand, E., Forest, K. T., Cambillau, C., Roussel, A., Voulhoux, R., Unraveling the self-assembly of the *Pseudomonas aeruginosa* XcpQ secretin periplasmic domain provides new molecular insights into type II secretion system secreton architecture and dynamics. *MBio* **8**, e01185-17 (2017)

60. X. Wang, C. Pineau, S. Gu, N. Guschinskaya, R. W. Pickersgill, V. E. Shevchik, Cysteine scanning mutagenesis and disulfide mapping analysis of arrangement of GspC and GspD protomers within the type 2 secretion system. *J. Biol. Chem.* **287**, 19082–19093 (2012).

61. T. Inoue, M. Forgac, Cysteine-mediated cross-linking indicates that subunit C of the V-ATPase is in close proximity to subunits E and G of the V1 domain and subunit a of the V0 domain. *J Biol Chem.* **280**, 27896-27903 (2005).

62. R. Van der Meeren, Y. Wen, P. Van Gelder, J. Tommassen, B. Devreese, S. N. Savvides, New insights into the assembly of bacterial secretins: Structural studies of the periplasmic domain of XcpQ from Pseudomonas aeruginosa. *J. Biol. Chem.* **288**, 1214–1225 (2013).

63. I. Guilvout, F. Samsudin, R. G. Huber, P. J. Bond, B. Bardiaux, O. Francetic, Membrane platform protein PulF of the Klebsiella type II secretion system forms a trimeric ion channel essential for endopilus assembly and protein secretion. *mBio* **15**, e0142323 (2024).

64. G. E. Crooks, G. Hon, J. M. Chandonia, S. E. Brenner, WebLogo: A sequence logo generator. *Genome Res.* **14**, 1188–1190 (2004).

65. S. Weber, C. Ramirez, W. Doerfler, Signal hotspot mutations in SARS-CoV-2 genomes evolve as the virus spreads and actively replicates in different parts of the world. *Virus Res.* **289**, 198170 (2020).

66. N. Dautin, G. Karimova, A. Ullmann, D. Ladant, Sensitive genetic screen for protease activity based on a cyclic AMP signaling cascade in Escherichia coli. *J. Bacteriol.* **182**, 7060–7066 (2000).

67. N. A. Treptow, H. A. Shuman, Genetic evidence for substrate and periplasmic-binding-protein recognition by the MalF and MalG proteins, cytoplasmic membrane components of the Escherichia coli maltose transport system. *J. Bacteriol.* **163**, 654–660 (1985).

68. E. Maguin, P. Duwat, T. Hege, D. Ehrlich, A. Gruss, New thermosensitive plasmid for gram-positive bacteria. *J. Bacteriol.* **174**, 5633–5638 (1992).

69. L. J. McGuffin, K. Bryson, D. T. Jones, The PSIPRED protein structure prediction server. *Bioinformatics* **16**, 404–405 (2000).

70. A. Drozdetskiy, C. Cole, J. Procter, G. J. Barton, JPred4: A protein secondary structure prediction server. *Nucleic Acids Res.* **43**, W389–W394 (2015).
